# Proportions and trends of global adolescent knowledge and attitudes toward tobacco smoking from 1999 to 2019

**DOI:** 10.3389/fpubh.2025.1546867

**Published:** 2025-08-18

**Authors:** Jintang Xie, Chuanwei Ma, Hui Yang, Zhuo Gong, Min Zhao, Costan G. Magnussen, Bo Xi

**Affiliations:** ^1^Department of Epidemiology, School of Public Health/Qilu Hospital, Cheeloo College of Medicine, Shandong University, Jinan, China; ^2^Department of Epidemiology and Health Statistics, School of Public Health, Guangdong Medical University, Dongguan, China; ^3^School of Public Health, Changsha Medical University, Changsha, China; ^4^Department of Nutrition and Food Hygiene, School of Public Health, Cheeloo College of Medicine, Shandong University, Jinan, China; ^5^Baker Heart and Diabetes Institute, Melbourne, VIC, Australia; ^6^Research Centre of Applied and Preventive Cardiovascular Medicine, University of Turku, Turku, Finland; ^7^Centre for Population Health Research, University of Turku and Turku University Hospital, Turku, Finland

**Keywords:** knowledge, attitude, tobacco smoking, adolescent, trends

## Abstract

**Objective:**

We aimed to assess the recent levels of knowledge and attitudes toward tobacco smoking among adolescents aged 12–16 years in 2010–2019, and to examine trends from 1999 to 2019.

**Methods:**

We used the most recent data from 145 countries/territories (hereafter “countries”) that conducted at least one Global Youth Tobacco Survey (GYTS) between 2010 and 2019 to assess the current levels of knowledge and attitudes toward tobacco smoking among adolescents aged 12–16 years. And 112 countries that conducted at least three GYTS surveys from 1999 to 2019 were used to assess the trends among adolescents aged 12–16 years over time.

**Results:**

Among 570,492 adolescents from 145 countries, 13.9% (95% *CI*, 11.9%−15.8%) incorrectly believed that tobacco smoking was not harmful, and 16.1% (95% *CI*, 15.2%−16.9%) believed that exposure to secondhand smoke was not harmful. A substantial proportion believed that quitting smoking was easy (42.5%; 95% *CI*, 36.9%−48.0%) or that short-term smoking was safe if followed by quitting (40.2%; 95% *CI*, 39.1%−41.3%). Additionally, 25.8% (95% *CI*, 24.8%−26.8%) believed that tobacco smoking helps young people feel more comfortable, 26.4% (95% *CI*, 24.8%−28.0%) believed that it helps them make more friends, and 15.8% (95% *CI*, 14.6%−17.0%) believed that it makes them appear more attractive. Among 1,734,258 adolescents from 112 countries, 67.9% of countries showed increasing or stable trends in the belief that smoking is not harmful, 75.9% for the belief that secondhand smoke exposure is not harmful, 38.4% for short-term smoking being safe, 32.1% for quitting being easy, and 69.6, 43.8, and 44.6% for the beliefs that smoking helps with comfort, making friends, and appearing attractive, respectively, from 1999 to 2019.

**Conclusions:**

Incorrect beliefs and positive attitudes toward tobacco smoking were prevalent among adolescents worldwide. Moreover, these beliefs and attitudes toward tobacco smoking have either persisted or increased in most included countries over time. Targeted interventions and policies are needed to reduce these and promote accurate knowledge about tobacco use and its harmful effects.

## Introduction

Tobacco use is a global public health issue associated with major chronic diseases (1–3). In 2019, it was estimated to account for 200 million disability-adjusted life-years (DALYs), and nearly eight million deaths due to smoking (4). Because the adolescent brain is not fully developed and nicotine affects synaptic function in the prefrontal cortex (5), adolescents are more vulnerable than adults to developing addiction to tobacco products. Therefore, it is crucial to implement preventive measures targeting tobacco use among adolescents. Recent statistics highlight the severity of this issue. According to the eighth WHO report on the global tobacco epidemic, over 24 million adolescents aged 13–15 years were current smokers in 2019 (6). Moreover, a review of data from the Global Youth Tobacco Survey (GYTS) conducted between 2010 and 2018 found that the prevalence of any tobacco use (on at least 1 day during the past 30 days) among adolescents aged 13–15 years was 17.9% for boys and 11.5% for girls (7). Using GYTS data from 2012 to 2019, Sun et al. (8) reported that 9.2% of adolescents aged 12–16 years had used e-cigarettes across the 67 countries and territories (hereafter “countries”) included in the study. Additionally, 62.9% of adolescents aged 12–16 years reported exposure to secondhand smoke on at least 1 day during the past 7 days in 142 countries (9). These findings underscore the widespread and ongoing nature of adolescent tobacco use and emphasize the urgent need for effective prevention strategies targeting adolescent tobacco use.

One approach to prevention involves enhancing knowledge and fostering health-conscious attitudes toward the risks associated with tobacco use. A more comprehensive understanding of the harmful effects of smoking and secondhand smoke exposure has been shown not only to reduce the likelihood of smoking initiation but also to increase the intention to quit among current smokers (10, 11). Lam et al. (12) reported that adolescents with greater awareness of the dangers of tobacco use were less likely to become smokers. Similarly, high school students who perceived smoking as less harmful or believed it had more advantages were more likely to initiate smoking over a 2-year period (13). Adolescents with lower perceptions of harm and addictiveness of tobacco products were associated with susceptibility to use tobacco and patterns of tobacco product use (14). Tapera et al. (15) found that adolescents with positive attitudes toward smoking were more likely to smoke. These findings are consistent with other studies identifying incorrect beliefs about smoking as significant predictors of adolescent tobacco and nicotine product use (16–18). However, existing evidence on adolescents' awareness of tobacco-related harms and their attitudes toward smoking is largely limited to local or regional studies. In Nigeria, 21.9% of non-smoking adolescents held incorrect beliefs about the harms of tobacco use, while 21.4% had positive attitudes toward smoking (19). Additionally, in Vietnam, ~12% of adolescents aged 13–15 years have inaccurate knowledge and attitudes toward tobacco use (20). Knowledge and attitudes toward tobacco smoking play a crucial role in controlling tobacco use among adolescents and in evaluating the effectiveness of tobacco control policies across countries. However, no previous studies have reported the levels and trends in knowledge and attitudes toward tobacco use among adolescents at the global level. Therefore, using data from the GYTS during 2010–2019 collected in 145 countries, we assessed the most recent levels of knowledge and attitudes toward tobacco smoking among adolescents aged 12–16 years. In addition, we assessed the trends in knowledge and attitudes toward tobacco use among adolescents in 112 countries from 1999 to 2019.

## Methods

### Study design and participants

The GYTS is a self-administered, school-based cross-sectional survey focusing on tobacco use and its associated factors among adolescents aged 12–16 years. A two-stage sampling procedure was used to obtain a typical random sample in each participating country. In the first stage, the probability proportional to size approach was employed to identify schools randomly based on enrollment size. In the second stage, classes within each selected school were chosen randomly. All adolescents in the selected classes were eligible to voluntarily participate in the survey. The standardized questionnaire was translated into the local language and then back-translated to ensure accuracy. The GYTS protocol has been authorized by the WHO and the US Centers for Disease Control and Prevention (CDC). The ethical boards in each country approved each GYTS. The GYTS does not contain any information that can be used to identify participants. Information on the GYTS is detailed on the website (21).

We analyzed the most recent data from 145 countries that conducted at least one survey between 2010 and 2019 to assess knowledge and attitudes toward tobacco smoking among adolescents. Additionally, data from 112 countries that conducted three or more surveys between 1999 and 2019 were used to assess the trends in knowledge and attitudes toward tobacco smoking. The inclusion and exclusion of participating countries are shown in Supplementary Figure S1. Participating adolescents with complete data on age and sex, as well as available data on knowledge and attitudes toward tobacco smoking, were included in this study.

### Definitions of knowledge and attitudes toward tobacco smoking

The GYTS assesses adolescents' knowledge and attitudes toward tobacco smoking through a set of standardized core questions across participating countries. Over time, some questions that were initially part of the core module became optional, allowing countries to adapt the survey to their specific national contexts. Knowledge was assessed using four questions (20, 22). Participants were classified as holding incorrect beliefs about tobacco use if they answered “*definitely not*” or “*probably not*” to any of the following questions: “*Do you think cigarette (tobacco) smoking is harmful to your health?*”; “*Do you think the smoke from other people's tobacco smoking is harmful to your health?*”; and “*Do you think that once someone has started smoking, it would be difficult for them to quit?*” Those who answered “*definitely yes*” or “*probably yes*” to the question “*Do you think it is safe to smoke for only one or two years as long as you quit after that?*” were also categorized as having incorrect beliefs about tobacco use.

Attitudes toward tobacco smoking were assessed using three questions: “*Do you think smoking tobacco helps people feel more comfortable or less comfortable at celebrations, parties, or other social gatherings?*”; “*Do you think young people who smoke tobacco have more or less friends?*”; and “*Do you think smoking tobacco makes young people look more or less attractive?*” Prior to 2012, the survey question about whether smoking helps young people make more friends or appear more attractive was divided into two separate queries: “*Whether smoking makes boys more friends and makes girls more friends*” and “*Whether smoking makes boys more attractive, and whether smoking makes girls more attractive*.” For our analysis, a positive attitude was defined as a response indicating that smoking tobacco helps young people feel more comfortable, enables them to make more friends, or makes them appear more attractive. Each question offered three response options: “*more*”, “*less*”, or “*no different*”. A response of “*more*” indicates a positive attitude toward tobacco smoking.

### Definitions of current cigarette use, any tobacco use, and secondhand smoke exposure

Current cigarette use was defined as having smoked cigarettes on at least 1 day during the past 30 days. Current any tobacco use was defined as having used cigarettes or other tobacco products (including chewing tobacco, snuff, dip, cigars, mini cigars, cigarillos, and pipes) on at least 1 day during the past 30 days (7). Secondhand exposure was defined as being exposed to secondhand smoke in any place (either at home or in public places) on at least 1 day during the past 7 days (9). All variables were assessed through self-reported responses from adolescents participating in school-based surveys.

### Additional survey variables

The income classification of each country was based on the World Bank categorization corresponding to the most recent GYTS survey year included in this study (23). The year of ratification of the Framework Convention on Tobacco Control (FCTC) for each country was obtained from the 2019 WHO Report on the Global Tobacco Epidemic (24). Additionally, the years in which countries achieved the highest level of implementation for policies related to monitoring tobacco use, warning about the dangers of tobacco, and banning tobacco advertising, as recommended by the FCTC, were extracted from the same WHO report (24).

### Statistical analyses

Proportion estimates and 95% confidence intervals (*CI*s) for knowledge and attitudes toward tobacco smoking in each country were calculated in consideration of original sampling weights, strata, and primary sampling units based on the GYTS approach employed in each country. The re-scaled weights in consideration of the sample size of each country were used to calculate recent levels of knowledge and attitudes toward tobacco between 2010 and 2019. Subgroup analyses were performed to assess potential factors influencing knowledge and attitudes toward tobacco smoking. These factors included sex, age group, WHO region, World Bank income category, current cigarette smoking, current use of any tobacco product, exposure to secondhand smoke, FCTC ratification status, and whether a country had implemented the highest level of policies related to monitoring tobacco use, warning about its dangers, and enforcing advertising bans. Differences across subgroups were assessed using chi-square tests.

A total of 112 countries that conducted three or more surveys from 1999 to 2019 were included to examine trends in knowledge and attitudes toward tobacco smoking. Modified Poisson regression analyses with robust error variance adjusted for survey year, sex, and age (continuous) were conducted to examine trends in knowledge and attitudes toward tobacco smoking (dichotomous) among adolescents for each country. Data from each country were assumed to follow a linear trend, and all available surveys in each country over time (1999–2019) were considered (7, 9). We also examined the trends (per five calendar years increase) in the levels of knowledge and positive attitudes toward tobacco smoking among adolescents. *P*-values of < 0.05 were considered statistically significant. All analyses were performed using R 4.4.2 with the “survey” package (version 4.4-2).

## Results

Our study analyzed the most recent data from 767,816 adolescents (boys: 48.3%, girls:51.7%) aged 12–16 years from 145 countries that had conducted at least one survey between Jan 1, 2010 and Dec 31, 2019, which were used to assess the latest levels of knowledge and attitudes toward tobacco smoking among adolescents aged 12–16 years. Among the 145 countries, 24 (16.6%) were in the African region, 30 (20.7%) in the American region, 24 (16.6%) in the Eastern Mediterranean region, 33 (22.8%) in the European region, nine (6.2%) in the South-East Asian region, and 25 (17.2%) in the Western Pacific region (Supplementary Table S1).

**Figure 1:**
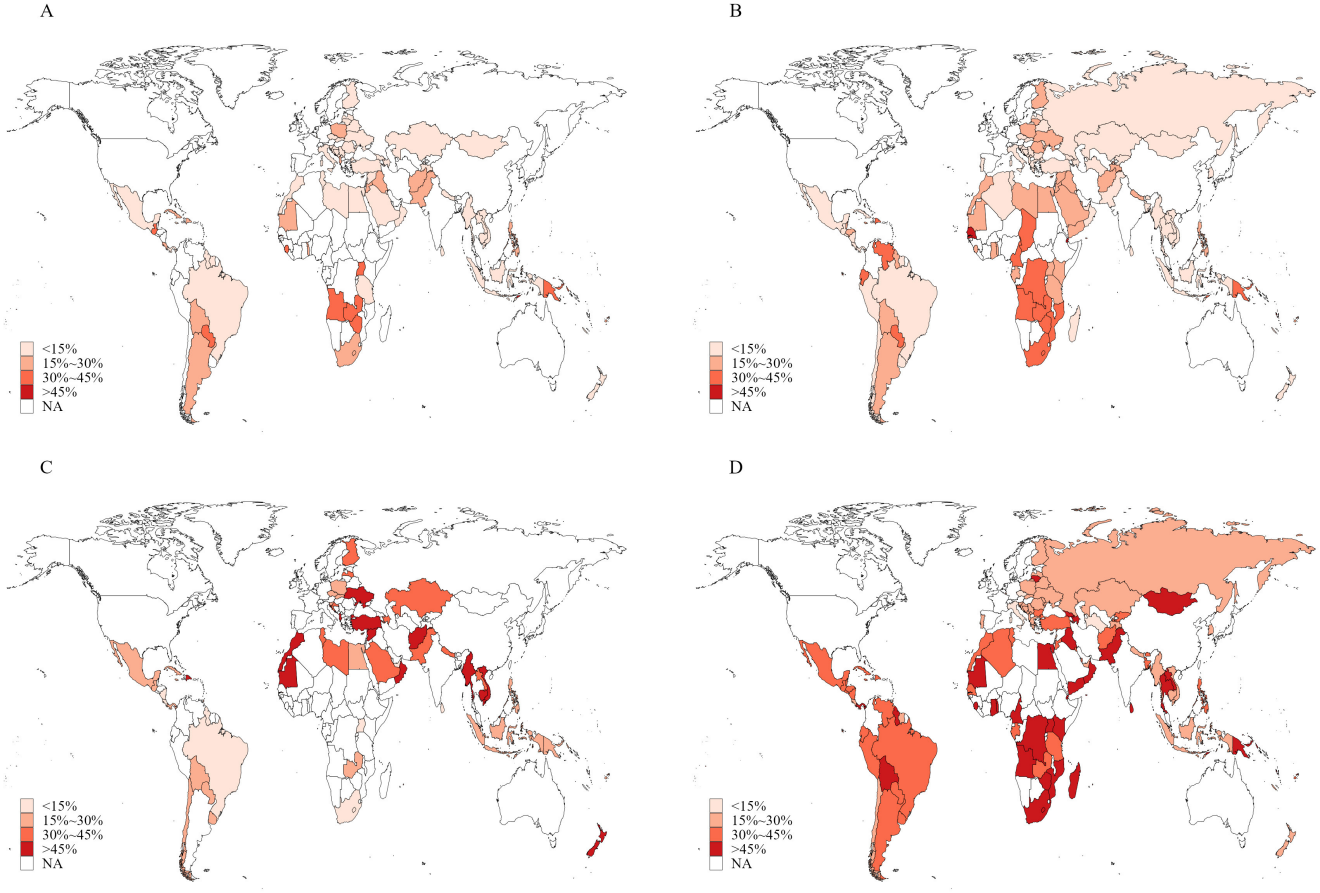
Proportions of adolescents aged 12–16 years with inaccurate knowledge about tobacco use in 145 countries/territories in 2010–2019. **(A)** Smoking tobacco is not harmful; **(B)** secondhand smoke exposure is not harmful; **(C)** it is safe to smoke for 1 or 2 years and then quit; **(D)** it is easy to quit smoking once someone started smoking.

Across all countries studied, 16.1% (95% *CI*: 15.2–16.9) of adolescents believed that secondhand smoke exposure was not harmful, and 13.9% (95% *CI*: 11.9–15.8) believed that tobacco smoking was not harmful. Additionally, 42.5% (95% *CI*: 36.9–48.0) believed that quitting smoking was easy; and 40.2% (95% *CI*: 39.1–41.3) believed that short-term smoking was safe if followed by quitting (Table 1). Incorrect beliefs about tobacco use were more prevalent among boys than girls. Adolescents aged 12–14 years were more likely to believe that smoking was safe if followed by quitting. Considerable variations were observed across regions and income levels. The proportions of adolescents who believed that tobacco smoking and secondhand smoke exposure were not harmful were highest in the African region. In contrast, the proportions who believed that quitting smoking was easy and that short-term smoking was safe were higher in the region of Americas and the Eastern Mediterranean region. Incorrect beliefs were more common in upper-middle-income countries and less prevalent in high-income countries (Table 1). Current cigarette smokers were more likely to believe that secondhand smoke exposure was not harmful. Similarly, current users of any tobacco products were more likely to believe that both secondhand smoke exposure and short-term smoking were not harmful. Paradoxically, adolescents who were not exposed to secondhand smoke were more likely to believe that tobacco use and secondhand smoke exposure were not harmful and that quitting smoking was easy. Adolescents in countries that had ratified the FCTC were less likely to believe that quitting smoking was easy or that short-term smoking was safe. Countries that had implemented the highest level of tobacco control policies had lower proportions of adolescents who held inaccurate knowledge about tobacco use. In these countries, adolescents were less likely to believe that secondhand smoke exposure was not harmful and that short-term smoking was safe. In contrast, adolescents in countries with the most stringent advertising bans were more likely to believe that quitting smoking was easy but less likely to believe that short-term smoking was safe. Levels of incorrect beliefs about tobacco use among adolescents varied across countries (Figure 1 and Supplementary Table S2).

**Table 1:** Proportions of adolescents aged 12–16 years with inaccurate knowledge about tobacco use.

	**No. of countries**	**Tobacco smoking is not harmful**	**No. of countries**	**Secondhand smoke exposure is not harmful**	**No. of countries**	**It is safe to smoke for 1 or 2 years and then quit**	**No. of countries**	**Once someone smokes, it is easy to quit**
Total	97	13.9 (11.9–15.8)	145	16.1 (15.2–16.9)	73	42.5 (36.9–48.0)	140	40.2 (39.1–41.3)
**Sex**								
Boys	97	17.1 (14.9–19.2)	145	18.1 (17.0–19.2)	73	41.4 (37.2–45.7)	140	44.1 (42.7–45.4)
Girls	97	11.0 (9.0–12.9)	145	13.9 (13.0–14.9)	73	43.4 (36.5–50.2)	140	36.1 (34.6–37.6)
*P*-value		<0.001		<0.001		0.24		<0.001
**Age group**								
12–14 years	97	14.2 (11.7–16.7)	145	15.6 (14.7–16.6)	73	45.2 (38.5–51.8)	140	39.8 (38.4–41.1)
15–16 years	97	13.2 (12.2–14.2)	145	16.9 (15.7–18.0)	73	36.1 (33.2–38.9)	140	41.0 (39.7–42.2)
*P*-value		0.34		0.04		0.001		0.11
**WHO region**								
Africa	12	23.2 (20.9–25.5)	24	24.6 (22.0–27.1)	7	13.4 (11.3–15.6)	23	48.8 (47.1–50.5)
Americas	19	16.2 (12.1–20.3)	30	18.8 (16.9–20.6)	19	51.1 (42.5–59.6)	30	38.0 (35.9–40.1)
Eastern Mediterranean	14	13.1 (11.6–14.6)	24	17.3 (15.1–19.5)	10	38.6 (34.8–42.4)	21	50.7 (46.4–54.9)
Europe	25	7.7 (6.9–8.5)	33	11.8 (11.0–12.6)	14	49.8 (47.9–51.6)	32	23.8 (22.2–25.4)
South-East Asia	6	8.8 (7.8–9.9)	9	9.8 (8.4–11.1)	4	29.6 (27.8–31.3)	9	38.1 (36.1–40.1)
Western Pacific	21	13.6 (12.5–14.8)	25	10.2 (9.5–10.9)	19	46.1 (42.2–49.9)	25	32.9 (31.7–34.2)
*P*-value		<0.001		<0.001		<0.001		<0.001
**World Bank income category**								
Low income	10	16.0 (14.3–17.8)	22	14.5 (12.9–16.2)	5	29.1 (25.8–32.4)	21	42.6 (40.9–44.4)
Lower-middle income	26	12.3 (11.4–13.3)	41	16.4 (14.6–18.3)	24	35.6 (33.5–37.8)	40	43.4 (41.3–45.6)
Upper-middle income	36	15.8 (12.2–19.5)	47	17.8 (16.4–19.1)	22	51.9 (44.7–59.0)	46	39.8 (38.1–41.5)
High income	25	6.8 (6.0–7.6)	35	12.2 (11.4–13.0)	22	32.4 (30.9–33.9)	33	19.0 (17.8–20.2)
*P*-value		0.001		0.002		<0.001		<0.001
**Current cigarette use^#^**								
Yes	96	16.1 (13.5–18.8)	145	19.3 (17.8–20.8)	73	46.8 (42.1–51.5)	140	37.5 (34.6–40.4)
No	96	13.6 (11.4–15.8)	145	15.7 (14.8–16.7)	73	42.1 (36.0–48.1)	140	40.4 (39.3–41.5)
*P-*value		0.16		<0.001		0.22		0.05
Current any tobacco use^*^								
Yes	95	17.2 (13.7–20.7)	144	20.4 (19.1–21.8)	72	53.1 (48.9–57.2)	139	39.1 (36.2–41.9)
No	95	12.7 (10.2–15.2)	144	15.0 (14.1–16.0)	72	41.4 (34.6–48.2)	139	40.0 (38.9–41.2)
*P*-value		0.07		<0.001		0.003		0.5
**Secondhand smoke exposure^∫^**								
Yes	96	12.1 (11.2–13.1)	144	13.5 (12.5–14.4)	72	41.2 (36.4–46.0)	139	36.0 (34.7–37.3)
No	96	16.4 (12.6–20.3)	144	20.2 (19.0–21.4)	72	44.2 (37.5–50.9)	139	47.1 (45.7–48.5)
*P*-value		0.005		<0.001		0.08		<0.001
**FCTC ratification**								
Yes	88	13.6 (12.7–14.5)	129	16.0 (15.1–16.9)	67	34.6 (32.9–36.3)	125	41.3 (40.2–42.5)
No	9	14.5 (9.1–19.8)	16	16.3 (13.0–19.7)	6	55.4 (48.4–62.3)	15	30.1 (26.7–33.4)
*P*-value		0.74		0.86		<0.001		<0.001
**Monitoring tobacco use**								
Yes	27	10.6 (9.5–11.7)	41	10.8 (9.6–12.0)	19	23.9 (21.1–26.7)	41	34.8 (32.8–36.8)
No	70	15.1 (12.6–17.6)	104	18.6 (17.5–19.6)	54	48.5 (43.1–53.8)	99	43.0 (41.7–44.2)
*P*-value		0.001		<0.001		<0.001		<0.001
**Warning about the dangers of tobacco**								
Yes	22	12.5 (10.8–14.2)	41	13.9 (12.7–15.2)	17	24.3 (21.6–26.9)	40	40.0 (38.1–41.9)
No	75	14.6 (11.9–17.3)	104	17.5 (16.4–18.7)	56	49.1 (43.9–54.4)	100	40.3 (39.2–41.4)
*P*-value		0.18		<0.001		<0.001		0.80
**Enforcing tobacco advertising bans**								
Yes	14	14.3 (11.8–16.8)	28	17.8 (16.1–19.5)	9	31.1 (29.2–33.1)	26	48.3 (46.4–50.3)
No	83	13.9 (11.9–15.9)	117	15.8 (14.9–16.8)	64	42.6 (37.1–48.2)	114	39.1 (38.0–40.3)
*P*-value		0.80		0.05		<0.001		<0.001

Data are presented as proportion (95% CI).

WHO, World Health Organization.

# Current cigarette use was defined as smoking a cigarette on at least 1 day during the past 30 days.

* Current any tobacco use was defined as using either cigarettes or other tobacco products (e.g., chewing tobacco, snuff, dip, cigars, cigarillos, pipe, e-cigarettes) on at least 1 day during the past 30 days.

∫ Current secondhand smoke exposure was defined as exposure to secondhand smoke in any place (at home or in public places) on at least 1 day during the past 7 days.

**Figure 2:**
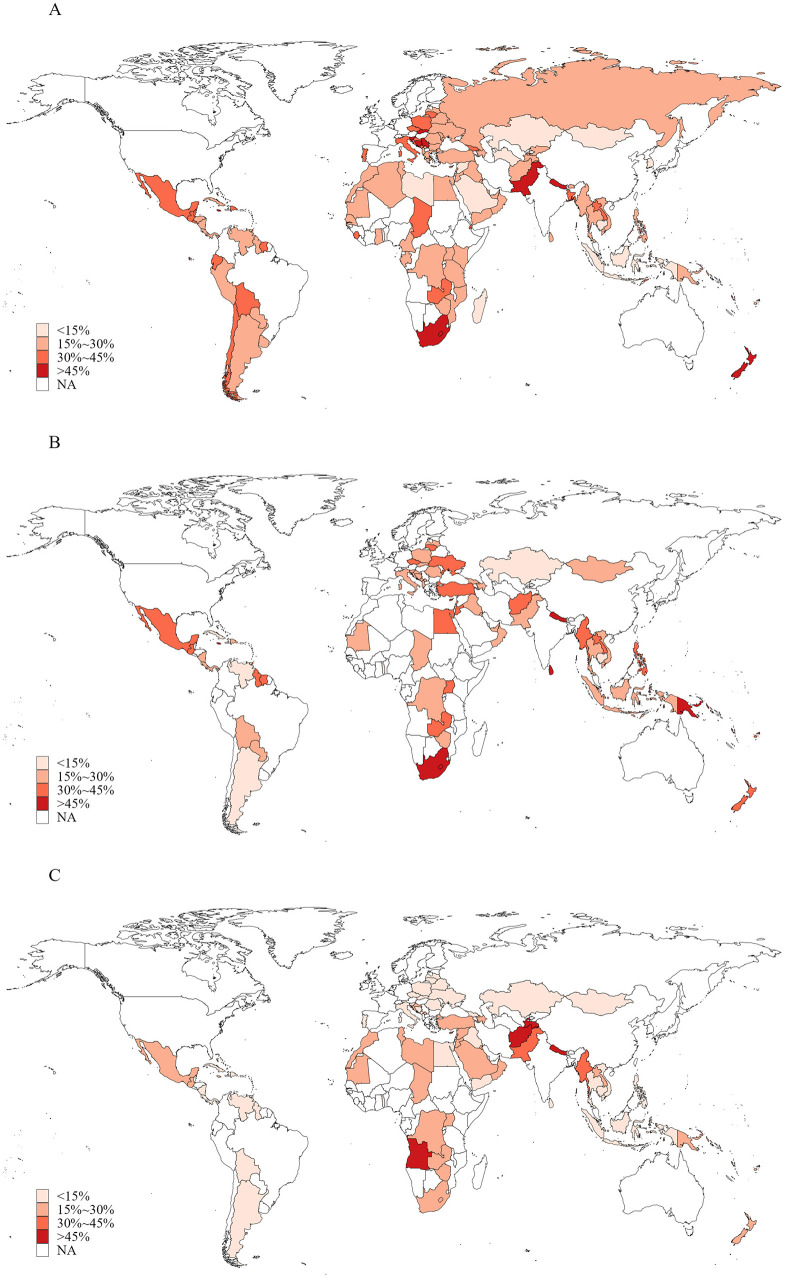
Proportions of adolescents aged 12–16 years with positive attitudes toward tobacco smoking in 145 countries/territories in 2010–2019. **(A)** Tobacco smoking is helpful for people feeling more comfortable; **(B)** smoking tobacco is helpful for making more friends; **(C)** smoking tobacco is helpful for being more attractive.

Overall, 25.8% (95% *CI*: 24.8%−26.8%) of included adolescents believed that tobacco smoking helped young people feel more comfortable, 26.4% (24.8–28.0) believed that it enabled them to make more friends, and 15.8% (14.6–17.0) believed that it made them appear more attractive (Table 2). These positive attitudes were more prevalent among boys compared to girls and among those aged 15–16 years compared to those aged 12–14 years. Regional variations were also observed. Adolescents in the European region reported the highest proportion who believed that tobacco smoking made them feel more comfortable, while the lowest proportion was found in the Western Pacific region. The African region had the highest proportion of adolescents who believed that tobacco smoking helped them make more friends, whereas the lowest was in the American region. The belief that tobacco smoking made adolescents appear more attractive was most common in the South-East Asian region and least common in the American region. Positive attitudes were most prevalent in low-income countries, while high-income countries had the highest proportion who believed that smoking helped young people feel more comfortable. Moreover, adolescents who were current smokers or were exposed to secondhand smoke were more likely to hold positive attitudes toward tobacco smoking. The proportions of adolescents with positive attitudes were also higher in countries that had ratified the FCTC and those with the highest level of policies warning about the dangers of tobacco use. As expected, these proportions were lower in countries with the highest level of policies monitoring tobacco use (Table 2). The proportions of these positive attitudes varied considerably across countries, as shown in Figure 2 and Supplementary Table S2.

**Table 2:** Proportions of adolescents aged 12–16 years with positive attitudes toward tobacco smoking.

	**No. of countries**	**Tobacco smoking helps young people feel more comfortable**	**No. of countries**	**Tobacco smoking makes more friends**	**No. of countries**	**Tobacco smoking is more attractive**
Total	141	25.8 (24.8–26.8)	89	26.4 (24.8–28.0)	96	15.8 (14.6–17.1)
**Sex**						
Boys	141	27.7 (26.3–29.1)	89	27.2 (25.1–29.2)	96	18.5 (17.1–19.9)
Girls	141	23.8 (22.5–25.0)	89	25.7 (23.6–27.8)	96	13.3 (11.7–14.8)
*P*-value		<0.001		0.30		<0.001
**Age group**						
12–14 years	141	24.9 (23.7–26.2)	89	24.6 (22.6–26.7)	96	14.2 (12.8–15.6)
15–16 years	141	27.4 (26.4–28.5)	89	30.8 (28.8–32.8)	96	19.7 (18.3–21.1)
*P*-value		<0.001		<0.001		<0.001
**WHO region**						
Africa	23	21.3 (20.2–22.4)	11	37.5 (34.9–40.0)	13	20.9 (19.2–22.6)
Americas	29	27.7 (26.2–29.3)	19	21.7 (20.1–23.3)	18	10.9 (9.2–12.6)
Eastern Mediterranean	24	21.2 (19.0–23.5)	14	30.7 (24.9–36.5)	17	18.9 (17.1–20.7)
Europe	32	33.1 (31.7–34.6)	20	22.7 (21.4–24.1)	23	14.8 (13.7–15.8)
South-East Asia	9	30.3 (27.3–33.2)	6	33.7 (30.8–36.6)	6	23.4 (20.7–26.0)
Western Pacific	24	17.3 (16.4–18.2)	19	26.5 (25.0–28.1)	19	13.1 (12.0–14.1)
*P*-value		<0.001		<0.001		<0.001
**World Bank income category**						
Low income	22	27.9 (24.9–30.9)	12	45.6 (42.0–49.2)	10	41.6 (39.1–44.2)
Lower-middle income	40	21.3 (19.9–22.7)	24	28.4 (25.2–31.6)	30	15.3 (14.1–16.5)
Upper-middle income	46	27.3 (26.1–28.5)	31	22.9 (21.5–24.3)	31	12.1 (10.7–13.5)
High income	33	32.6 (30.9–34.3)	22	22.7 (21.0–24.3)	25	15.5 (14.1–17.0)
*P*-value		<0.001		<0.001		<0.001
**Current cigarette use^#^**						
Yes	141	36.0 (33.4–38.6)	89	30.9 (27.4–34.3)	96	21.6 (17.5–25.7)
No	141	24.8 (23.8–25.9)	89	25.9 (24.3–27.5)	96	15.2 (14.1–16.3)
*P-*value		<0.001		0.002		<0.001
Current any tobacco use^*^						
Yes	140	36.1 (33.9–38.3)	87	30.7 (26.9–34.4)	94	20.4 (17.4–23.4)
No	140	24.6 (23.5–25.7)	87	25.8 (24.0–27.6)	94	15.0 (13.6–16.3)
*P*-value		<0.001		0.002		<0.001
**Secondhand smoke exposure^∫^**						
Yes	141	27.4 (26.1–28.7)	88	29.0 (27.2–30.8)	95	16.8 (15.4–18.1)
No	141	23.1 (22.1–24.2)	88	23.6 (20.4–26.8)	95	15.2 (12.9–17.5)
*P*-value		<0.001		0.008		0.24
**FCTC ratification**						
Yes	126	26.5 (25.5–27.6)	77	28.9 (27.3–30.6)	85	18.6 (17.5–19.6)
No	15	19.0 (16.7–21.3)	12	21.4 (19.2–23.6)	11	9.5 (7.4–11.7)
*P*-value		<0.001		<0.001		<0.001
**Monitoring tobacco use**						
Yes	40	23.4 (21.9–24.8)	23	26.9 (23.8–30.0)	24	12.8 (11.3–14.3)
No	101	26.9 (25.7–28.2)	66	26.2 (24.4–28.0)	72	16.8 (15.0–18.5)
*P*-value		0.001		0.68		0.001
**Warning about the dangers of tobacco**						
Yes	40	26.0 (24.5–27.4)	22	25.1 (22.9–27.4)	21	12.7 (11.4–13.9)
No	101	25.7 (24.3–27.0)	67	27.3 (24.9–29.8)	75	17.6 (15.4–19.7)
*P*-value		0.77		0.18		<0.001
**Enforcing tobacco advertising bans**						
Yes	27	15.9 (14.9–17.0)	13	23.7 (22.3–25.1)	13	15.5 (13.8–17.2)
No	114	27.1 (25.9–28.2)	76	26.5 (24.8–28.1)	83	15.8 (14.6–17.1)
*P*-value		<0.001		0.02		0.74

Data are presented as proportion (95% CI).

WHO, World Health Organization.

# Current cigarette use was defined as smoking a cigarette on at least 1 day during the past 30 days.

* Current any tobacco use was defined as using either cigarettes or other tobacco products (e.g., chewing tobacco, snuff, dip, cigars, cigarillos, pipe, e-cigarettes) on at least 1 day during the past 30 days.

∫ Current secondhand smoke exposure was defined as exposure to secondhand smoke in any place (at home or in public places) on at least 1 day during the past 7 days.

The proportions of knowledge and attitudes toward tobacco smoking among adolescents stratified by current tobacco use status are shown in Supplementary Tables S3, S4 (by country) and Supplementary Tables S5, S6 (by other subgroups). Generally, a higher proportion of current adolescent smokers had incorrect beliefs about tobacco use and held positive attitudes toward tobacco smoking compared to their non-smoking peers. Similarly, Supplementary Tables S7–S10 present corresponding data stratified by current exposure to secondhand smoke. Overall, a lower proportion of adolescents exposed to secondhand smoke held incorrect beliefs on tobacco use. Furthermore, they were more likely to hold positive attitudes toward tobacco smoking compared to those not exposed to secondhand smoke.

**Figure 3:**
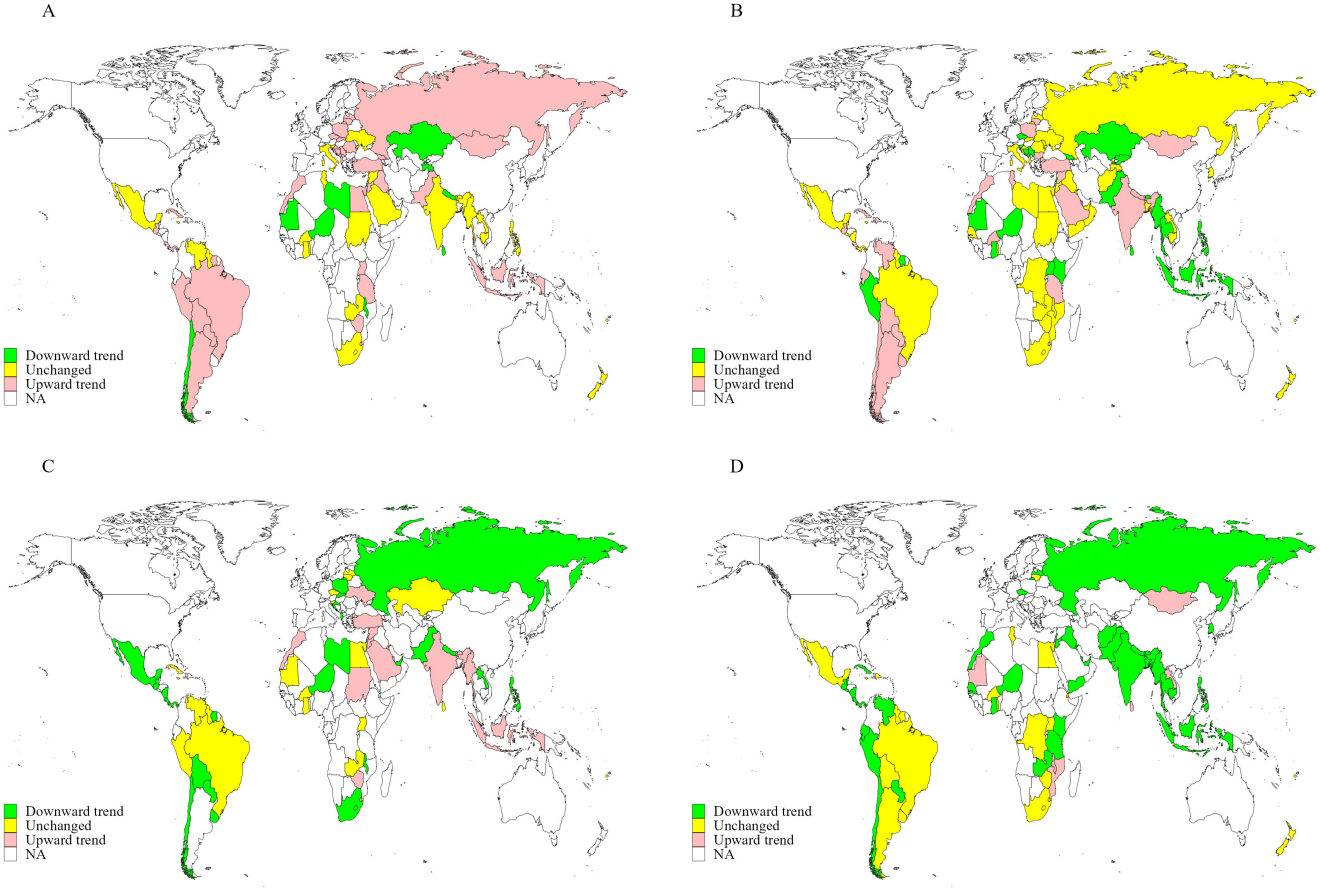
Trends in the proportions of adolescents aged 12–16 years with incorrect knowledge about tobacco smoking in 155 countries/territories from 1999 to 2019. **(A)** Smoking tobacco is not harmful; **(B)** secondhand smoke exposure is not harmful; **(C)** it is safe to smoke for 1 or 2 years and then quit; **(D)** it is easy to quit smoking once someone started smoking.

**Figure 4:**
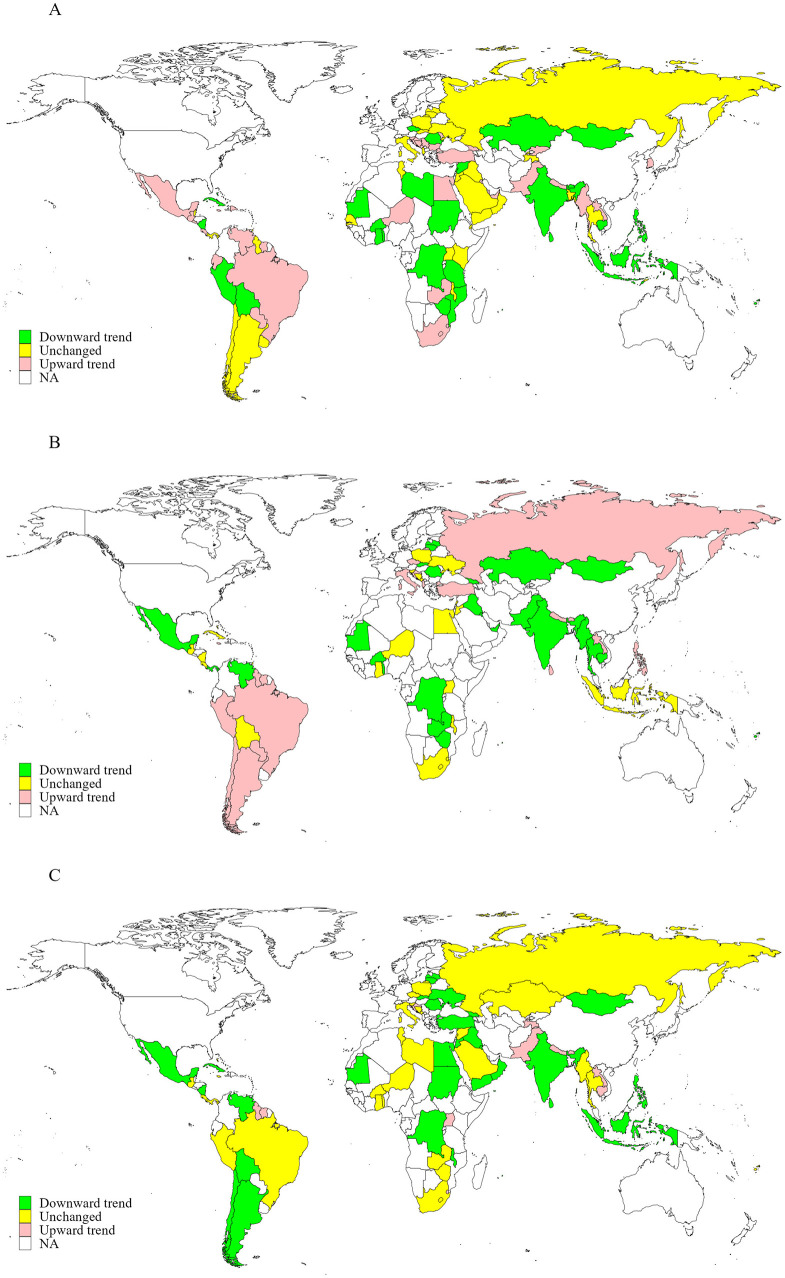
Trends in the proportions of adolescents aged 12–16 years with positive attitudes toward tobacco smoking in 155 countries/territories from 1999 to 2019. **(A)** Tobacco smoking is helpful for people feeling more comfortable; **(B)** smoking tobacco is helpful for making more friends; **(C)** smoking tobacco is helpful for being more attractive.

A total of 112 countries conducted three or more rounds of GYTS between Jan 1, 1999, and Dec 31, 2019. Data from 1,734,258 adolescents aged 12–16 years were used to assess trends in incorrect beliefs and positive attitudes toward tobacco smoking. As shown in Tables 3, 4, the proportions of adolescents with incorrect beliefs about tobacco use and positive attitudes toward tobacco smoking increased in ~6.9%−49.4% of countries from 1999 to 2019 [43 countries (49.4%) believed that tobacco smoking was not harmful, 32 (28.8%) believed that secondhand smoke exposure was not harmful, 18 (26.1%) believed that short-term smoking was safe, 6 (6.9%) believed that quitting smoking was easy, and 40 (37.0%), 23 (29.5%), and 12 (14.0%), respectively, believed that tobacco smoking was helpful for people feeling more comfortable, making more friends, and being more attractive]. Meanwhile, the proportions of adolescents holding these incorrect beliefs and positive attitudes leveled off in ~33.3%−47.7% of countries from 1999 to 2019 [33 countries (37.9%) believed that tobacco smoking was not harmful, 53 (47.7%) believed that secondhand smoke exposure was not harmful, 25 (36.2%) believed that short-term smoking was safe, 30 (34.5%) believed that quitting smoking was easy, and 38 (35.2%), 26 (33.3%) and 38 (44.2%), respectively, believed that tobacco smoking was helpful for people feeling more comfortable, making more friends, and being more attractive]. Trends in inaccurate knowledge and positive attitudes varied by subgroups including age, sex, WHO region, current cigarette use, current any tobacco use, current secondhand smoke exposure, ratification status of the FCTC, and whether countries had implemented the highest level of policies to monitor tobacco use, warn about the dangers of tobacco use, and enforce tobacco advertising bans (Table 3). The proportions and changes (per five calendar-years increase) between the first and last survey in knowledge and attitudes toward tobacco smoking in each country from 1999 to 2019 are presented in Figures 3, 4 and Supplementary Tables S11–S14.

**Table 3:** Proportions of countries with upward, downward, and unchanged trends in inaccurate knowledge about smoking tobacco among adolescents aged 12–16 years from 1999 to 2019.

**Group**	**Tobacco smoking is not harmful**				**Secondhand smoke exposure is not harmful**				**It is safe to smoke for 1 or 2 years and then quit**				**Once someone smokes, it is easy to quit**			
	**No. of countries**	**Down** ^β^	**Up** ^β^	**Unchanged** ^θ^	**No. of countries**	**Down** ^β^	**Up** ^β^	**Unchanged** ^θ^	**No. of countries**	**Down** ^β^	**Up** ^β^	**Unchanged** ^θ^	**No. of countries**	**Down** ^β^	**Up** ^β^	**Unchanged** ^θ^
Total	87	12.6	49.4	37.9	111	23.4	28.8	47.7	69	37.7	26.1	36.2	87	58.6	6.9	34.5
**Age group**																
12–14 years	87	10.3	47.1	42.5	111	18.9	31.5	49.5	69	37.7	27.5	34.8	87	46.0	11.5	42.5
15–16 years	86	11.6	33.7	54.7	110	28.2	19.1	52.7	68	39.7	22.1	38.2	87	60.9	5.7	33.3
**Sex**																
Boys	87	11.5	36.8	51.7	111	21.6	20.7	57.7	69	37.7	26.1	36.2	87	54.0	8.0	37.9
Girls	87	10.3	44.8	44.8	111	16.2	27.9	55.9	69	34.8	24.6	40.6	87	49.4	9.2	41.4
**WHO region**																
Africa	13	23.1	30.8	46.2	18	27.8	16.7	55.6	11	27.3	18.2	54.5	18	44.4	16.7	38.9
Americas	23	8.7	56.5	34.8	29	13.8	44.8	41.4	24	41.7	12.5	45.8	29	51.7	0.0	48.3
Eastern Mediterranean	13	7.7	46.2	46.2	20	10.0	40.0	50.0	11	27.3	63.6	9.1	15	73.3	0.0	26.7
Europe	21	9.5	76.2	14.3	24	33.3	20.8	45.8	12	41.7	16.7	41.7	4	75.0	0.0	25.0
South-East Asia	8	37.5	25.0	37.5	10	60.0	20.0	20.0	6	16.7	66.7	16.7	10	90.0	10.0	0.0
Western Pacific	9	0	22.2	77.8	10	10.0	10.0	80.0	5	80.0	0.0	20.0	11	45.5	18.2	36.4
**World Bank income category**																
Low income	9	44.4	33.3	22.2	15	20.0	26.7	53.3	6	50.0	16.7	33.3	14	50.0	14.3	35.7
Lower-middle income	25	8.0	36.0	56.0	33	33.3	27.3	39.4	20	30.0	45.0	25.0	27	63.0	14.8	22.2
Upper-middle income	34	11.8	55.9	32.4	39	23.1	23.1	53.8	25	40.0	12.0	48.0	28	60.7	0.0	39.3
High income	19	5.3	63.2	31.6	24	12.5	41.7	45.8	18	38.9	27.8	33.3	18	55.6	0.0	44.4
**Current cigarette use^#^**																
Yes	87	4.6	42.5	52.9	110	10.9	20.9	68.2	69	18.8	14.5	66.7	87	26.4	5.7	67.8
No	87	11.5	44.8	43.7	111	23.4	27.9	48.6	69	37.7	24.6	37.7	87	58.6	5.7	35.6
Current any tobacco use^*^																
Yes	85	3.5	44.7	51.8	108	12.0	24.1	63.9	67	23.9	16.4	59.7	85	29.4	10.6	60.0
No	85	9.4	41.2	49.4	108	26.9	29.6	43.5	67	37.3	23.9	38.8	85	58.8	7.1	34.1
**Secondhand smoke exposure^∫^**																
Yes	79	6.3	46.8	46.8	105	23.8	23.8	52.4	63	36.5	23.8	39.7	87	57.5	8.0	34.5
No	79	10.1	39.2	50.6	105	18.1	35.2	46.7	63	28.6	23.8	47.6	87	55.2	9.2	35.6
**FCTC ratification**																
Yes	80	11.2	48.8	40.0	103	24.3	28.2	47.6	62	35.5	24.2	40.3	79	60.8	6.3	32.9
No	7	28.6	57.1	14.3	8	12.5	37.5	50.0	7	57.1	42.9	0.0	8	37.5	12.5	50.0
**Monitoring tobacco use**																
Yes	26	7.7	57.7	34.6	36	30.6	25.0	44.4	18	44.4	22.2	33.3	24	70.8	8.3	20.8
No	61	14.8	45.9	39.3	75	20.0	30.7	49.3	51	35.3	27.5	37.3	63	54.0	6.3	39.7
**Warning about the dangers of tobacco**																
Yes	21	9.5	52.4	38.1	33	18.2	33.3	48.5	14	42.9	14.3	42.9	27	48.1	7.4	44.4
No	66	13.6	48.5	37.9	78	25.6	26.9	47.4	55	36.4	29.1	34.5	60	63.3	6.7	30.0
**Enforcing tobacco advertising bans**																
Yes	11	27.3	54.5	18.2	22	22.7	18.2	59.1	6	66.7	0.0	33.3	18	61.1	5.6	33.3
No	76	10.5	48.7	40.8	89	23.6	31.5	44.9	63	34.9	28.6	36.5	69	58.0	7.2	34.8

Data are presented as proportion.

WHO, World Health Organization.

# Current cigarette use was defined as smoking a cigarette on at least 1 day during the past 30 days.

* Current any tobacco use was defined as using either cigarettes or other tobacco products (e.g., chewing tobacco, snuff, dip, cigars, cigarillos, pipe, e-cigarettes) on at least 1 day during the past 30 days.

∫ Current secondhand smoke exposure was defined as exposure to secondhand smoke in any place (at home or in public places) on at least 1 day during the past 7 days.

Proportions of countries with upward, downward, and unchanged trends were based on Poisson regression analysis used to examine the trend across all survey years in each country and territory (^β^P for trend < 0.05; ^θ^P for trend >0.05) in Supplementary Tables S12–S14.

**Table 4:** Proportions of countries with upward, downward, and unchanged trends in positive attitudes about smoking tobacco among adolescents aged 12–16 years from 1999 to 2019.

**Group**	**Tobacco smoking helps people feel more comfortable**				**Tobacco smoking makes more friends**				**Tobacco smoking is more attractive**			
	**No. of countries**	**Down** ^β^	**Up** ^β^	**Unchanged** ^θ^	**No. of countries**	**Down** ^β^	**Up** ^β^	**Unchanged** ^θ^	**No. of countries**	**Down** ^β^	**Up** ^β^	**Unchanged** ^θ^
Total	108	27.8	37.0	35.2	78	37.2	29.5	33.3	86	41.9	14.0	44.2
**Age group**												
12–14 years	108	22.2	35.2	42.6	78	35.9	25.6	38.5	86	32.6	12.8	54.7
15–16 years	107	30.8	29.0	40.2	78	35.9	24.4	39.7	85	48.2	14.1	37.6
**Sex**												
Boys	108	27.8	34.3	38.0	78	37.2	21.8	41.0	86	47.7	9.3	43.0
Girls	108	23.1	32.4	44.4	78	28.2	19.2	52.6	86	25.6	16.3	58.1
**WHO region**												
Africa	18	50.0	22.2	27.8	13	53.8	0.0	46.2	14	35.7	14.3	50.0
Americas	29	17.2	58.6	24.1	24	16.7	50.0	33.3	23	34.8	13.0	52.2
Eastern Mediterranean	18	22.2	27.8	50.0	7	57.1	0.0	42.9	15	60.0	6.7	33.3
Europe	23	13.0	43.5	43.5	18	33.3	33.3	33.3	18	38.9	11.1	50.0
South-East Asia	10	50.0	20.0	30.0	8	37.5	37.5	25.0	8	37.5	25.0	37.5
Western Pacific	10	40.0	20.0	40.0	8	62.5	25.0	12.5	8	50.0	25.0	25.0
**World Bank income category**												
Low income	14	35.7	21.4	42.9	7	42.9	14.3	42.9	9	22.2	33.3	44.4
Lower-middle income	32	43.8	31.2	25.0	21	42.9	19.0	38.1	26	57.7	15.4	26.9
Upper-middle income	38	23.7	52.6	23.7	32	31.2	43.8	25.0	31	32.3	9.7	58.1
High income	24	8.3	29.2	62.5	18	38.9	22.2	38.9	20	45.0	10.0	45.0
**Current cigarette use^#^**												
Yes	108	8.3	33.3	58.3	78	7.7	23.1	69.2	85	28.0	10.6	61.2
No	108	30.6	34.3	35.2	78	37.2	28.2	34.6	86	36.0	15.1	48.8
Current any tobacco use^*^												
Yes	105	10.5	36.2	53.3	77	14.3	24.7	61.0	84	23.8	10.7	65.5
No	105	28.6	35.2	36.2	77	37.7	28.6	33.8	84	34.5	20.2	45.2
**Secondhand smoke exposure^∫^**												
Yes	103	25.2	36.9	37.9	71	36.6	26.8	36.6	78	42.3	11.5	46.2
No	103	27.2	25.2	47.6	71	39.4	9.9	50.7	78	28.2	11.5	60.3
**FCTC ratification**												
Yes	99	27.3	39.4	33.3	73	37.0	30.1	32.9	78	39.7	15.4	44.9
No	9	33.3	11.1	55.6	5	40.0	20.0	40.0	8	62.5	0.0	37.5
**Monitoring tobacco use**												
Yes	35	25.7	40.0	34.3	23	47.8	26.1	26.1	23	52.2	17.4	30.4
No	73	28.8	35.6	35.6	55	32.7	30.9	36.4	63	38.1	12.7	49.2
**Warning about the dangers of tobacco**												
Yes	32	25.0	31.2	43.8	19	52.6	31.6	15.8	18	72.2	0.0	27.8
No	76	28.9	39.5	31.6	59	32.2	28.8	39.0	68	33.8	17.6	48.5
**Enforcing tobacco advertising bans**												
Yes	20	35.0	25.0	40.0	10	50.0	30.0	20.0	10	50.0	0.0	50.0
No	88	26.1	39.8	34.1	68	35.3	29.4	35.3	76	40.8	15.8	43.4

Data are presented as proportion.

WHO, World Health Organization.

# Current cigarette use was defined as smoking a cigarette on at least 1 day during the past 30 days.

* Current any tobacco use was defined as using either cigarettes or other tobacco products (e.g., chewing tobacco, snuff, dip, cigars, cigarillos, pipe, e-cigarettes) on at least 1 day during the past 30 days.

∫ Current secondhand smoke exposure was defined as exposure to secondhand smoke in any place (at home or in public places) on at least 1 day during the past 7 days.

Proportions of countries with upward, downward, and unchanged trends were based on Poisson regression analysis used to examine the trend across all survey years in each country and territory (^β^P for trend < 0.05; ^θ^P for trend >0.05) in Supplementary Tables S12–S14.

Overall, the proportions of adolescents with inaccurate knowledge about tobacco use and positive attitudes toward tobacco smoking either increased or remained unchanged between 1999 and 2019 with two exceptions: the belief that quitting smoking is easy and the belief that tobacco smoking makes people more attractive, both of which decreased by 2.4 and 1.0%, respectively, per 5-year period (Table 5). The proportion of adolescents who believed that tobacco smoking is not harmful increased, particularly among boys, adolescents aged 15–16 years, current smokers, and in countries without MPOWER measures. Other beliefs, such as “secondhand smoke exposure is not harmful,” “it is safe to smoke for one or two years and then quit,” and attitudes like “smoking helps people feel more comfortable” or “smoking helps make more friends,” remained relatively stable. However, the belief that “once someone smokes, it is hard to quit” and the attitude that “smoking makes people more attractive” showed declining trends across most subgroups.

**Table 5:** Linear trends (per 5 calendar years) in the proportions of adolescents aged 12–16 years with incorrect beliefs and positive attitudes toward tobacco smoking from 1999 to 2019.

**Group**	**Tobacco smoking is not harmful**	**Secondhand smoke exposure is not harmful**	**It is safe to smoke for 1 or 2 years and then quit**	**Once someone smokes, it is easy to quit**	**Tobacco smoking helps people feel more comfortable**	**Tobacco smoking makes more friends**	**Tobacco smoking is attractive**
Total	1.2 (0.4 to 2.1)	0.5 (−0.1 to 1.1)	−0.2 (−2.8 to 2.5)	−2.4 (−3.2 to −1.5)	0.6 (−0.3 to 1.6)	−0.2 (−1.4 to 0.9)	−1.0 (−2.0 to 0.0)
**Sex**							
Boys	1.9 (1.0 to 2.8)	1.0 (0.4 to 1.7)	−0.2 (−2.8 to 2.4)	−1.5 (−2.4 to −0.6)	0.7 (−0.2 to 1.6)	−0.2 (−1.3 to 1.0)	−0.8 (−1.7 to 0.1)
Girls	0.5 (−0.4 to 1.4)	−0.1 (−0.7 to 0.5)	−0.4 (−3.2 to 2.4)	−3.3 (−4.3 to −2.3)	0.5 (−0.6 to 1.5)	−0.3 (−1.5 to 0.8)	−1.1 (−2.3 to 0.0)
**Age group**							
12–14 years	0.9 (0.0 to 1.8)	0.3 (−0.3 to 0.9)	−0.1 (−2.8 to 2.5)	−2.6 (−3.5 to −1.6)	0.4 (−0.5 to 1.3)	−0.4 (−1.5 to 0.8)	−1.0 (−2.1 to 0.1)
15–16 years	1.7 (0.7 to 2.6)	0.7 (0.0 to 1.4)	−0.3 (−3.0 to 2.4)	−2.1 (−3.0 to −1.1)	0.9 (−0.1 to 2.0)	−0.0 (−1.1 to 1.1)	−0.8 (−1.7 to 0.2)
**WHO region**							
Africa	0.9 (−2.4 to 4.2)	−0.5 (−2.7 to 1.8)	0.3 (−3.2 to 3.9)	−1.7 (−4.4 to 1.0)	−1.9 (−3.7 to −0.0)	−3.5 (−5.8 to −1.1)	−0.7 (−2.0 to 0.7)
Americas	2.5 (1.1 to 3.9)	1.7 (0.6 to 2.8)	−5.0 (−8.5 to −1.5)	−1.9 (−3.1 to −0.7)	1.6 (0.5 to 2.7)	1.9 (0.3 to 3.5)	0.3 (−1.0 to 1.6)
Eastern Mediterranean	1.2 (−0.6 to 3.0)	1.7 (0.4 to 2.9)	11.9 (−0.8 to 24.6)	−4.0 (−5.8 to −2.2)	1.2 (−2.9 to 5.3)	−2.1 (−4.8 to 0.6)	−3.0 (−6.4 to 0.4)
Europe	1.2 (0.6 to 1.8)	−0.5 (−1.7 to 0.7)	−0.8 (−4.3 to 2.8)	−2.0 (−4.8 to 0.9)	1.8 (0.2 to 3.4)	0.2 (−1.1 to 1.5)	−1.0 (−2.7 to 0.7)
South-East Asia	−0.9 (−8.3 to 6.5)	−0.9 (−3.2 to 1.4)	0.7 (−6.2 to 7.5)	−4.6 (−8.0 to −1.2)	0.1 (−4.1 to 4.3)	1.0 (−7.4 to 9.4)	−0.8 (−7.5 to 6.0)
Western Pacific	0.2 (−0.4 to 0.8)	0.2 (−1.2 to 1.5)	−4.3 (−9.6 to 1.1)	−0.5 (−3.4 to 2.3)	−0.9 (−3.5 to 1.8)	−2.2 (−5.9 to 1.6)	−1.5 (−6.6 to 3.6)
**World Bank income category**							
Low income	0.4 (−3.8 to 4.6)	0.9 (−1.4 to 3.2)	−2.2 (−10.1 to 5.7)	−2.1 (−5.8 to 1.5)	−0.8 (−3.9 to 2.3)	−2.6 (−8.6 to 3.4)	2.0 (−2.3 to 6.3)
Lower-middle income	1.9 (0.4 to 3.4)	−0.2 (−1.6 to 1.1)	3.0 (−4.1 to 10.0)	−2.5 (−4.4 to −0.7)	0.2 (−2.3 to 2.7)	−0.2 (−3.0 to 2.6)	−2.1 (−4.5 to 0.3)
Upper-middle income	0.6 (−0.9 to 2.2)	0.5 (−0.4 to 1.5)	−1.7 (−4.9 to 1.5)	−2.5 (−3.7 to −1.4)	1.5 (0.2 to 2.8)	−0.2 (−1.7 to 1.4)	−1.1 (−2.6 to 0.3)
High income	1.8 (0.6 to 2.9)	1.1 (0.3 to 1.9)	−0.8 (−6.3 to 4.6)	−2.1 (−3.4 to −0.7)	0.6 (−0.2 to 1.5)	0.4 (−1.4 to 2.2)	−0.6 (−1.6 to 0.4)
**Current cigarette use^#^**							
Yes	2.4 (1.3 to 3.5)	1.0 (0.1 to 1.9)	0.1 (−1.8 to 2.0)	−1.4 (−2.5 to −0.3)	2.0 (1.0 to 2.9)	1.6 (0.5 to 2.8)	−0.9 (−2.0 to 0.3)
No	1.1 (0.3 to 2.0)	0.5 (−0.1 to 1.1)	−0.0 (−2.8 to 2.7)	−2.4 (−3.3 to −1.5)	0.6 (−0.4 to 1.5)	−0.4 (−1.5 to 0.7)	−0.8 (−1.8 to 0.2)
Current any tobacco use^*^							
Yes	2.0 (0.9 to 3.1)	1.1 (0.3 to 1.9)	0.1 (−2.1 to 2.3)	−1.9 (−3.0 to −0.8)	2.2 (1.2 to 3.2)	1.1 (0.0 to 2.2)	−1.0 (−2.1 to 0.1)
No	1.1 (0.3 to 2.0)	0.4 (−0.2 to 1.0)	−0.3 (−3.1 to 2.4)	−2.5 (−3.4 to −1.5)	0.7 (−0.3 to 1.7)	−0.3 (−1.4 to 0.9)	−0.7 (−1.8 to 0.3)
**Secondhand smoke exposure^∫^**							
Yes	1.2 (0.3 to 2.1)	0.3 (−0.3 to 0.9)	−0.1 (−2.9 to 2.7)	−2.7 (−3.6 to −1.7)	0.8 (−0.2 to 1.8)	0.2 (−1.1 to 1.4)	−1.1 (−2.1 to −0.0)
No	1.2 (0.1 to 2.2)	0.9 (0.1 to 1.7)	0.1 (−2.9 to 3.1)	−2.3 (−3.3 to −1.4)	0.5 (−0.5 to 1.5)	−1.2 (−2.4 to −0.1)	−0.8 (−1.8 to 0.3)
**FCTC ratification**							
Yes	1.3 (0.4 to 2.2)	0.3 (−0.3 to 0.9)	−0.2 (−3.1 to 2.6)	−2.6 (−3.5 to −1.7)	0.8 (−0.2 to 1.8)	−0.1 (−1.2 to 1.0)	−0.8 (−1.9 to 0.3)
No	0.5 (−2.6 to 3.5)	2.4 (−0.7 to 5.6)	0.5 (−8.7 to 9.7)	−0.0 (−4.3 to 4.2)	−1.5 (−3.7 to 0.8)	−2.6 (−9.5 to 4.3)	−2.8 (−4.6 to −1.0)
**Monitoring tobacco use**							
Yes	1.3 (0.4 to 2.2)	−0.0 (−0.9 to 0.8)	−1.7 (−6.9 to 3.5)	−2.7 (−4.3 to −1.1)	0.9 (−0.4 to 2.1)	−0.9 (−2.5 to 0.7)	−1.8 (−3.6 to −0.0)
No	1.2 (0.0 to 2.4)	0.7 (−0.1 to 1.5)	0.4 (−2.8 to 3.6)	−2.2 (−3.3 to −1.2)	0.5 (−0.7 to 1.8)	0.0 (−1.4 to 1.5)	−0.7 (−1.9 to 0.5)
**Warning about the dangers of tobacco**							
Yes	1.2 (0.2 to 2.2)	0.7 (−0.1 to 1.6)	−3.5 (−7.4 to 0.4)	−1.7 (−3.1 to −0.4)	0.4 (−1.0 to 1.7)	−0.8 (−2.6 to 0.9)	−2.5 (−4.5 to −0.5)
No	1.2 (0.1 to 2.3)	0.4 (−0.4 to 1.2)	0.7 (−2.5 to 3.9)	−2.7 (−3.8 to −1.5)	0.7 (−0.5 to 2.0)	−0.1 (−1.4 to 1.3)	−0.6 (−1.7 to 0.6)
**Enforcing tobacco advertising bans**							
Yes	−2.5 (−7.3 to 2.3)	−0.7 (−2.3 to 0.9)	−3.9 (−7.2 to −0.5)	−2.0 (−3.8 to −0.1)	−0.7 (−2.2 to 0.8)	−1.4 (−3.9 to 1.1)	−3.6 (−7.1 to 0.0)
No	1.7 (1.1 to 2.4)	0.8 (0.1 to 1.4)	0.2 (−2.7 to 3.1)	−2.5 (−3.5 to −1.5)	0.9 (−0.2 to 2.0)	−0.1 (−1.3 to 1.2)	−0.6 (−1.7 to 0.4)

Data are presented as proportion (95% CI).

WHO, World Health Organization.

# Current cigarette use was defined as smoking a cigarette on at least 1 day during the past 30 days.

* Current any tobacco use was defined as using either cigarettes or other tobacco products (e.g., chewing tobacco, snuff, dip, cigars, cigarillos, pipe, e-cigarettes) on at least 1 day during the past 30 days.

∫ Current secondhand smoke exposure was defined as exposure to secondhand smoke in any place (at home or in public places) on at least 1 day during the past 7 days.

## Discussion

Based on the most recent GYTS data from 145 countries conducted between 2010 and 2019, substantial proportions of adolescents held inaccurate knowledge about tobacco use (~14%−43%) and held positive attitudes toward tobacco smoking (16%−26%), believing it helps young people feel more comfortable, make more friends, and appear more attractive. Additionally, from 1999 to 2019, 112 countries conducted at least three rounds of the GYTS, collectively including data from 1,734,258 adolescents to assess trends in knowledge and attitudes toward tobacco smoking. Among these countries, the proportions of adolescents with inaccurate knowledge about tobacco use increased in ~6.9%−49.4% of countries, while 29%−48% showed no significant change. Similarly, the proportions of countries where adolescents expressed positive attitudes toward tobacco smoking increased in ~14%−37% of countries and remained stable in about 33%−44% during the same period.

Between 2010 and 2019, 13.9% of adolescents perceived tobacco smoking as harmless, and 16.0% perceived secondhand smoke exposure as harmless. Additionally, over 40% believed that short-term tobacco use was harmless and that quitting smoking was easy. Previous research has highlighted that awareness of tobacco-related hazards is a key determinant of adolescent tobacco use among adolescents (16, 25, 26). Bernat et al. (27) reported that 21.1 and 52.8% of U.S. adolescents aged 14–17 considered e-cigarettes and secondhand e-cigarette smoke to be harmless, respectively. Similarly, 19% of middle school students in Mexico perceived e-cigarettes as less harmful than conventional cigarettes (28). Moreover, adolescents in countries with more stringent tobacco hazard warning policies demonstrated greater awareness of the adverse effects of tobacco smoking, secondhand smoke exposure, and short-term smoking. However, no previous study has assessed adolescents' knowledge regarding the short-term effect of tobacco use or the perceived difficulty of quitting. Our findings indicate inaccurate knowledge about tobacco use remains prevalent and underscore the need for strengthened health education efforts to address these incorrect beliefs among adolescents.

Our finding that a substantial proportion of adolescents across various countries maintained positive attitudes toward tobacco smoking between 2010 and 2019 is consistent with previous findings. For instance, a 2008 study of 649 Swedish adolescents aged 17–18 reported that 18.2% held incorrect beliefs about tobacco smoking (29), while a 2014 study among 3,430 Vietnamese adolescents aged 13–15 found that 30.8% held positive attitudes toward smoking (20). Moreover, a 2015 study in Thailand reported that 27.7% of 1,721 adolescents aged 13–15 believed that tobacco smoking helped young people feel more comfortable (30). Collectively, these findings suggest that despite numerous global, regional, and national initiatives, many adolescents continue to hold favorable perceptions of tobacco smoking. Furthermore, a significant proportion of adolescents use alternative tobacco products, such as e-cigarettes, and maintain positive attitudes toward them (31), which may contribute to future increases in traditional cigarette smoking (32). Our study found that adolescents in countries with the most stringent tobacco surveillance policies or advertising bans exhibited lower proportions of positive attitudes toward smoking, suggesting that such policies may help mitigate these perceptions. Given the persistently high levels of positive attitudes, continued efforts to enhance tobacco-related knowledge and restrict smoking behaviors should be prioritized. These efforts should include further implementation of the MPOWER framework and strengthened collaboration between governments and families to improve tobacco education.

The proportions of adolescents exhibiting positive attitudes toward tobacco smoking were higher among current tobacco users and those exposed to secondhand smoke than among their counterparts. This pattern is consistent with findings from Jiang et al., (33) who reported that Hong Kong secondary school students who used waterpipes had a higher prevalence of positive attitudes toward smoking than non-users. Similarly, a longitudinal study of Swedish adolescents found that current tobacco users were more likely to hold incorrect beliefs about smoking than non-users (29). The higher proportions of positive attitudes among these subgroups may reflect a more favorable perception of tobacco use. This trend was particularly evident among boys, older adolescents aged 15–16 years, and those in low-income countries or regions where tobacco smoking is more socially accepted, such as Europe, Africa, and South-East Asia (7). The widespread prevalence of positive attitudes in these regions and demographic groups may be attributable to the higher rates of tobacco use (7, 34). These findings underscore the urgent need for targeted interventions, particularly among current adolescent tobacco users, those exposed to secondhand smoke, and adolescents in low-income countries or regions where tobacco smoking remains culturally accepted.

According to research by Chen et al. (35) school-based smoking interventions significantly improved awareness and attitudes toward tobacco smoking among Chinese adolescents. Similarly, Rozi et al. (36) found that stronger interventions in secondary schools in Karachi were associated with higher knowledge scores regarding the risks of smokeless tobacco use. In addition, community-based prevention programs have been shown to reduce cigarette use and increase cessation rates among adolescents (37). These findings highlight the importance of family- and school-based interventions in promoting accurate knowledge of tobacco-related harms and fostering critical attitudes toward tobacco use.

We observed that adolescents' inaccurate knowledge about tobacco use and positive attitudes toward tobacco smoking, did not improve, and even worsened, from 1999 to 2019, especially among boys and adolescents aged 15–16 years. Despite widespread tobacco control measures, including advertising restrictions, increased taxation, plain packaging, and health warning labels, the enforcement of these policies varies across countries due to political and economic factors (38–40). Previous studies have confirmed the crucial role of knowledge and attitudes in shaping adolescent smoking behaviors (41–43). Ma et al. (7) reported that while the global prevalence of cigarette use among adolescents aged 13–15 years decreased from 1999 to 2018, the use of other tobacco products either remained unchanged or increased in many countries. The high prevalence of tobacco use among adults in some countries may contribute to adolescents' incorrect perceptions (44, 45). Furthermore, the emergence of new tobacco products, such as e-cigarettes, often marketed as less harmful, may serve as a major source of misinformation (46). The widespread advertising of these products can blur the perceived dangers of tobacco. Further studies should focus on the impact of new tobacco products on adolescents' knowledge and attitudes toward smoking, and how e-cigarettes influence adolescent smoking prevalence.

Despite global and national initiatives, such as the implementation of the FCTC and MPOWER, the prevalence of tobacco use, including e-cigarettes and other tobacco products, remained high among adolescents (8, 9, 47). This persistent trend is consistent with widespread presence of inaccurate knowledge and positive attitudes toward tobacco smoking. Sustained efforts are required to reduce adolescent tobacco use, including implementing effective interventions that improve knowledge and attitudes toward smoking and tobacco products, while also strengthening measures that restrict adolescent access to tobacco (48). Promoting accurate awareness of tobacco-related risks is a shared responsibility that requires coordinated action from governments, schools, communities, and families to foster a healthier, smoke-free environment.

### Strengths and limitations

Our study has several strengths. First, it used recent GYTS data between 2010 and 2019 across 145 countries, providing the most current and comprehensive assessment of adolescents' knowledge and attitudes toward tobacco smoking. In addition, data from an expanded set of 112 countries enabled us to assess the long-term trends in adolescents' knowledge and attitudes toward tobacco use from 1999 to 2019, coinciding with the implementation of global tobacco control initiatives. Second, the standardization questionnaire employed across all participating countries allowed for direct cross-national comparisons of proportions. Third, the study assessed the impact of diverse tobacco control policies on knowledge and attitudes toward tobacco smoking in adolescents from a global perspective. However, our study also has several limitations. First, the use of self-reported data may have introduced recall or reporting bias. Second, our findings may not be generalizable to out-of-school adolescents, as the sample consisted exclusively of students aged 12–16 years. Third, findings may not be generalizable to other age groups, as the study focused exclusively on adolescents aged 12–16 years. Fourth, data from some countries were not nationally representative, as sampling was limited to select cities or regions. Finally, trend analyses were limited to countries with at least three survey rounds, potentially limiting the ability to detect non-linear temporal patterns in countries with fewer data points.

## Conclusions

Our findings reveal that a significant proportion of adolescents worldwide continue to hold incorrect beliefs and positive attitudes toward tobacco smoking. This highlights the urgent need for more effective interventions and improved implementation of existing strategies, such as the FCTC and MPOWER frameworks. Efforts should prioritize high-risk groups, including boys, adolescents aged 15–16, and those in low-income countries or the African region. To enhance knowledge of the harms of tobacco use and foster healthier attitudes, targeted community, school, and family-based programs, particularly for current smokers, are crucial.

## Data Availability

The datasets presented in this study can be found in online repositories. The names of the repository/repositories and accession number(s) can be found in the article/Supplementary material.
