# Supplementary material for: Proportions and trends of global adolescent knowledge and attitudes toward tobacco smoking from 1999 to 2019
Source: Front Public Health. 2025 Aug 18;13:1546867. doi: 10.3389/fpubh.2025.1546867 (PMC12399639; doi:10.3389/fpubh.2025.1546867)
Supplement: Supplementary file 1 [file Data_Sheet_1.doc]

**Supplemental Information**

**
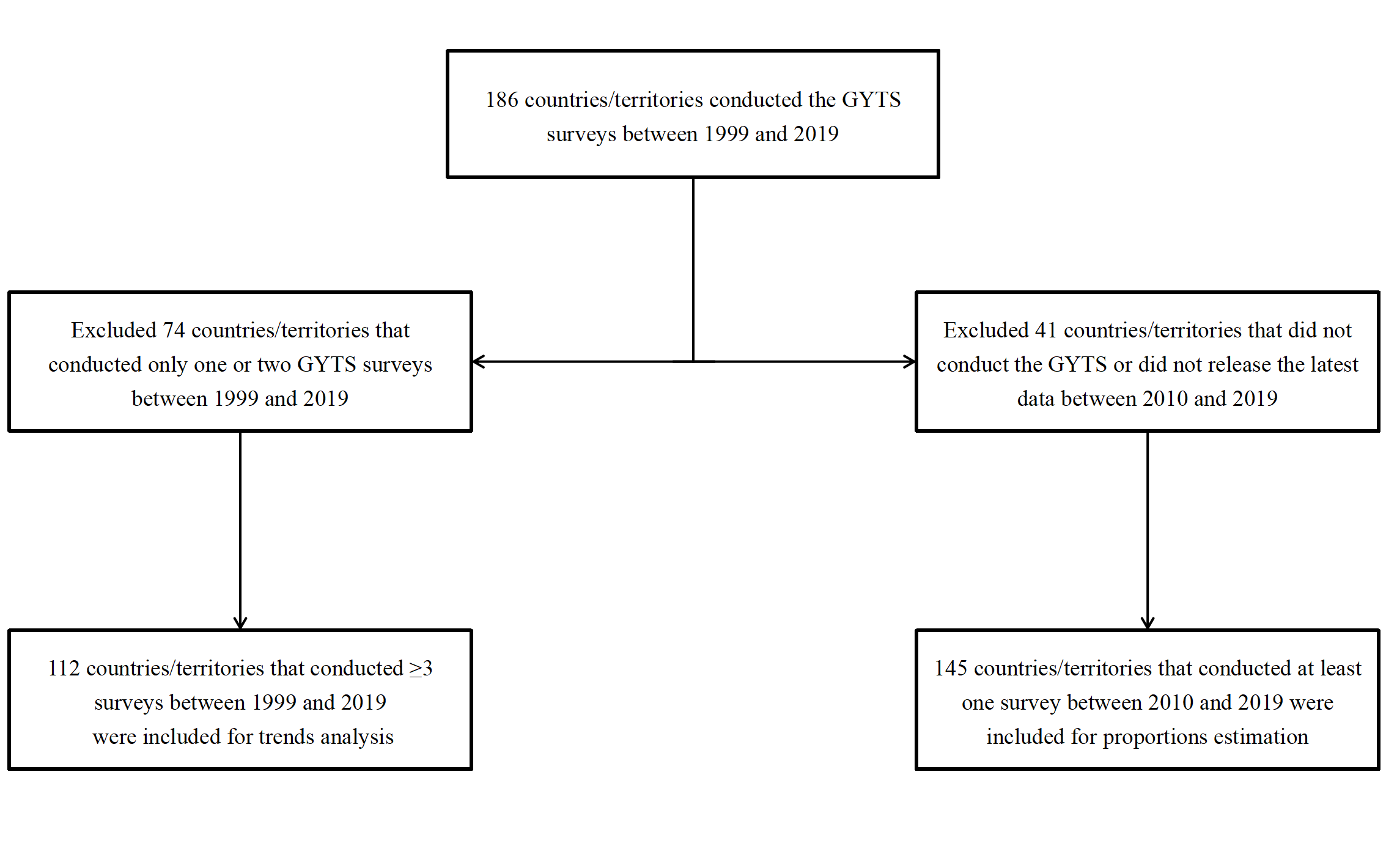
**

**Figure S1 Inclusion and exclusion of participating countries**

**Table S1 Characteristics of Global Youth Tobacco Surveys of adolescents aged 12-16 years in 145 countries/territories, 2010-2019**

| **Country/territory** | **Representativeness** | **Survey year** | **Sample size** | **Boys, %** |
| --- | --- | --- | --- | --- |
| **Africa** |  |  |  |  |
| Algeria | National | 2013 | 5248 | 45.8 |
| Angola | Subnational | 2010 | 1161 | 57.6 |
| Cameroon | National | 2014 | 2404 | 55.1 |
| Chad | National | 2019 | 1469 | 60.8 |
| Comoros | National | 2015 | 2238 | 45.4 |
| Congo | National | 2019 | 5035 | 51.1 |
| Gabon | National | 2014 | 1185 | 42.1 |
| Gambia | National | 2017 | 8026 | 41.5 |
| Ghana | National | 2017 | 5480 | 47.7 |
| Kenya | National | 2013 | 1737 | 46.2 |
| Madagascar | National | 2018 | 2318 | 45.4 |
| Mauritania | National | 2018 | 3401 | 45.3 |
| Mauritius | National | 2016 | 3961 | 46.6 |
| Mozambique | National | 2013 | 4238 | 47.1 |
| Sao Tome and Principe | National | 2010 | 5583 | 45.2 |
| Senegal | National | 2013 | 1232 | 50.1 |
| Seychelles | National | 2015 | 2393 | 47.1 |
| Sierra Leone | National | 2017 | 4587 | 43.8 |
| South Africa | National | 2011 | 6163 | 44.3 |
| Togo | National | 2019 | 3181 | 55.7 |
| Uganda | National | 2018 | 2959 | 44.1 |
| United Republic of Tanzania | National | 2016 | 3548 | 46.8 |
| Zambia | National | 2011 | 2767 | 47.5 |
| Zimbabwe | National | 2014 | 6082 | 44.4 |
| **Americas** |  |  |  |  |
| Antigua and Barbuda | National | 2017 | 1973 | 50.5 |
| Argentina | National | 2018 | 1405 | 49.2 |
| Bahamas | National | 2013 | 1288 | 47.5 |
| Barbados | National | 2013 | 1681 | 50.6 |
| Belize | National | 2014 | 1724 | 43.6 |
| Bolivia | National | 2018 | 4647 | 51.9 |
| Brazil | Subnational | 2011 | 1416 | 45.8 |
| Chile | Subnational | 2016 | 10941 | 48.2 |
| Costa Rica | National | 2013 | 2815 | 48.5 |
| Cuba | National | 2018 | 4071 | 48.9 |
| Dominican Republic | National | 2016 | 1227 | 45.0 |
| Ecuador | National | 2016 | 5084 | 50.5 |
| El Salvador | National | 2015 | 2983 | 47.5 |
| Grenada | National | 2016 | 2028 | 48.5 |
| Guatemala | National | 2015 | 3988 | 51.5 |
| Guyana | National | 2015 | 1536 | 43.8 |
| Honduras | National | 2016 | 3330 | 47.6 |
| Jamaica | National | 2017 | 1397 | 44.8 |
| Mexico | National | 2011 | 2444 | 47.4 |
| Nicaragua | National | 2019 | 7619 | 47.4 |
| Panama | National | 2017 | 2548 | 49.1 |
| Paraguay | National | 2019 | 4585 | 47.6 |
| Peru | National | 2019 | 3852 | 49.0 |
| Saint Kitts and Nevis | National | 2010 | 756 | 46.0 |
| Saint Lucia | National | 2017 | 1170 | 47.9 |
| Saint Vincent and the Grenadines | National | 2018 | 1312 | 44.8 |
| Suriname | National | 2016 | 1779 | 46.7 |
| Trinidad and Tobago | National | 2017 | 3528 | 43.3 |
| Uruguay | National | 2014 | 4619 | 46.9 |
| Venezuela | National | 2019 | 6328 | 46.6 |
| **Eastern Mediterranean** | |  |  |  |
| Afghanistan | Subnational | 2017 | 1444 | 52.6 |
| Bahrain | National | 2015 | 3324 | 49.0 |
| Djibouti | National | 2013 | 1658 | 51.9 |
| Egypt | National | 2014 | 2300 | 58.6 |
| Gaza Strip | Subnational | 2019 | 1485 | 44.6 |
| Iraq | National | 2019 | 2073 | 65.4 |
| Jordan | National | 2014 | 2107 | 55.0 |
| Kuwait | National | 2016 | 2380 | 45.4 |
| Lebanon | National | 2011 | 2133 | 48.2 |
| Libyan Arab Jamahiriya | National | 2010 | 1761 | 50.7 |
| Morocco | National | 2016 | 3723 | 49.0 |
| Oman | National | 2016 | 2020 | 46.1 |
| Pakistan | National | 2013 | 7849 | 44.8 |
| Qatar | National | 2018 | 1982 | 49.3 |
| Saudi Arabia | National | 2010 | 2221 | 47.1 |
| Syrian Arab Republic | National | 2010 | 1602 | 39.6 |
| Tunisia | National | 2017 | 2401 | 44.4 |
| United Arab Emirates | National | 2013 | 4029 | 44.3 |
| UNRWA GAZA | Regional | 2013 | 1672 | 59.0 |
| UNRWA Jordan | Regional | 2014 | 1343 | 44.8 |
| UNRWA Lebanon | Regional | 2013 | 1406 | 46.7 |
| UNRWA Westbank | Regional | 2014 | 1481 | 52.2 |
| West BANK | Regional | 2016 | 1344 | 42.9 |
| Yemen | National | 2014 | 1904 | 53.2 |
| **Europe** |  |  |  |  |
| Albania | National | 2015 | 4488 | 47.9 |
| Azerbaijan | National | 2016 | 2215 | 50.6 |
| Belarus | National | 2015 | 2947 | 50.0 |
| Bosnia and Herzegovina | National | 2019 | 5328 | 51.8 |
| Bulgaria | National | 2015 | 3997 | 47.2 |
| Croatia (Hrvatska) | National | 2016 | 3221 | 51.4 |
| Cyprus | National | 2011 | 1036 | 49.2 |
| Czech Republic | National | 2016 | 3914 | 49.4 |
| Finland | National | 2012 | 4945 | 49.3 |
| Georgia | National | 2017 | 1300 | 49.4 |
| Greece | National | 2013 | 4562 | 50.7 |
| Italy | National | 2018 | 1644 | 48.5 |
| Kazakhstan | National | 2014 | 2048 | 52.7 |
| Kosovo | National | 2016 | 4938 | 49.2 |
| Kyrgyzstan | National | 2019 | 6057 | 49.1 |
| Latvia | National | 2019 | 4181 | 51.2 |
| Lithuania | National | 2018 | 3000 | 49.6 |
| Macedonia | National | 2016 | 5083 | 50.7 |
| Malta | National | 2017 | 1244 | 55.7 |
| Montenegro | National | 2018 | 4149 | 49.6 |
| Poland | National | 2016 | 5005 | 48.0 |
| Portugal | National | 2013 | 10602 | 46.6 |
| Republic of Moldova | National | 2019 | 4647 | 50.4 |
| Romania | National | 2017 | 5339 | 48.5 |
| Russian Federation | Subnational | 2015 | 6523 | 49.8 |
| San Marino | National | 2018 | 606 | 55.6 |
| Serbia | National | 2017 | 3802 | 49.6 |
| Slovakia | National | 2016 | 3965 | 50.0 |
| Slovenia | National | 2017 | 2503 | 46.1 |
| Tajikistan | National | 2019 | 3956 | 50.3 |
| Turkey | National | 2017 | 113509 | 49.7 |
| Turkmenistan | National | 2015 | 8263 | 49.5 |
| Ukraine | National | 2017 | 4006 | 49.6 |
| **South-East Asia** |  |  |  |  |
| Bangladesh | National | 2013 | 3232 | 43.5 |
| Bhutan | National | 2019 | 3340 | 44.3 |
| Indonesia | National | 2019 | 8035 | 44.7 |
| Maldives | National | 2011 | 4148 | 44.9 |
| Myanmar | National | 2016 | 3469 | 45.9 |
| Nepal | National | 2011 | 2380 | 47.8 |
| Sri Lanka | National | 2015 | 1499 | 51.2 |
| Thailand | National | 2015 | 1864 | 45.8 |
| Timor-Leste | National | 2019 | 2519 | 38.0 |
| **Western Pacific** |  |  |  |  |
| Brunei Darussalam | National | 2019 | 2248 | 46.5 |
| Cambodia | National | 2016 | 2785 | 42.6 |
| Cook Islands | National | 2016 | 482 | 48.1 |
| Fiji | National | 2016 | 2469 | 45.6 |
| Guam | National | 2017 | 1805 | 49.8 |
| Kiribati | National | 2018 | 1921 | 44.6 |
| Laos | National | 2016 | 5647 | 47.5 |
| Macao (China) | Regional | 2015 | 1644 | 49.5 |
| Marshall Islands | National | 2016 | 2277 | 43.4 |
| Micronesia | National | 2013 | 5192 | 44.8 |
| Mongolia | National | 2019 | 4105 | 48.3 |
| New Caledonia | National | 2010 | 912 | 47.6 |
| New Zealand | National | 2010 | 1336 | 50.9 |
| Niue | National | 2019 | 118 | 46.6 |
| Northern Mariana Islands | National | 2014 | 2122 | 51.6 |
| Palau | National | 2017 | 1053 | 49.4 |
| Papua New Guinea | National | 2016 | 1874 | 49.4 |
| Philippines | National | 2019 | 9713 | 44.9 |
| Samoa | National | 2017 | 1483 | 37.0 |
| South Korea | National | 2013 | 4185 | 46.1 |
| Tokelau | National | 2014 | 96 | 53.1 |
| Tonga | National | 2010 | 1986 | 45.1 |
| Tuvalu | National | 2018 | 601 | 45.3 |
| Vanuatu | National | 2017 | 1289 | 38.0 |
| Viet Nam | National | 2014 | 3528 | 48.5 |
| Total | NA | NA | 570492 | 48.2 |

Abbreviation: NA, not applicable.

**Table** **S2 Prevalence of inaccurate beliefs and positive attitudes toward tobacco smoking among adolescents aged 12–16 years, by country/territory, 2010–2019**

| **Country/territory** | **Tobacco smoking is not harmful** | **Secondhand smoke exposure is not harmful** | **It is safe to smoke one or two years then quit** | **Once someone smoke, it is easy to quit** | **Tobacco smoking helps people feel more comfortable** | **Tobacco smoking makes more friends** | **Tobacco smoking is more attractive** |
| --- | --- | --- | --- | --- | --- | --- | --- |
| **Africa** |  |  |  |  |  |  |  |
| Algeria | NA | 8.9(7.5-10.2) | NA | 35.4(33.5-37.3) | 30.0(28.0-32.0) | NA | NA |
| Angola | 36.3(27.6-45.0) | 33.9(24.9-42.8) | NA | 77.6(66.3-88.8) | NA | NA | 46.5(35.8-57.1) |
| Cameroon | NA | 35.0(29.0-41.0) | NA | 54.6(47.0-62.2) | 23.6(19.3-27.8) | NA | NA |
| Chad | NA | 40.7 (38.2-43.2) | NA | NA | 30.6 (28.2-33.0) | 22.1 (20.0-24.3) | 24.8 (22.5-27.1) |
| Comoros | NA | 40.4(34.0-46.8) | NA | 49.3(45.3-53.2) | 25.3(21.0-29.6) | NA | NA |
| Congo | NA | 39.0(33.6-44.4) | NA | 66.4(62.0-70.9) | 15.5(12.7-18.4) | 26.1(23.2-29.0) | 25.4(22.3-28.5) |
| Gabon | NA | 27.8(23.2-32.3) | NA | 30.2(25.7-34.8) | 18.5(16.1-20.9) | NA | NA |
| Gambia | 41.3(36.0-46.5) | 34.0(29.9-38.0) | 19.2(16.3-22.1) | 61.7(58.1-65.4) | 27.2(25.0-29.5) | 22.7(20.4-24.9) | 12.7(11.0-14.5) |
| Ghana | NA | 29.3(25.5-33.1) | NA | 50.6(46.8-54.5) | 17.9(15.2-20.6) | NA | NA |
| Kenya | NA | 16.5(10.6-22.3) | NA | 52.0(47.6-56.4) | 15.3(13.1-17.6) | NA | NA |
| Madagascar | NA | 5.0(3.1-7.0) | NA | 48.6(40.9-56.3) | 7.6(5.0-10.1) | NA | NA |
| Mauritania | 28.3(23.0-33.6) | 29.5(24.5-34.6) | 46.1(40.5-51.7) | 67.8(64.2-71.4) | 21.0(15.4-26.5) | 27.7(25.0-30.5) | 28.8(26.4-31.2) |
| Mauritius | 13.7(9.3-18.1) | 15.5(10.5-20.5) | 17.5(15.6-19.5) | 28.3(24.5-32.1) | 17.7(14.3-21.0) | 28.0(22.8-33.1) | 10.8(8.0-13.5) |
| Mozambique | NA | 31.0(27.0-35.0) | NA | 47.0(43.3-50.6) | 23.0(20.9-25.2) | NA | NA |
| Sao Tome and Principe | 19.9 (18.9-21.0) | 22.3 (21.2-23.4) | 41.0 (39.7-42.3) | 57.4 (56.1-58.7) | 34.0 (32.7-35.2) | 20.7 (19.7-21.8) | 29.2(28.0-30.4) |
| Senegal | NA | 49.5(35.6-63.5) | NA | 44.1(35.5-52.7) | 24.1(19.0-29.1) | NA | NA |
| Seychelles | NA | 28.7(24.9-32.4) | NA | 38.5(34.9-42.1) | 27.2(24.5-29.9) | NA | 13.1(11.3-14.9) |
| Sierra Leone | 31.5(26.3-36.7) | 24.0(19.0-29.0) | NA | 58.8(52.8-64.8) | 38.9(32.9-44.9) | NA | NA |
| South Africa | 29.6(25.5-33.7) | 32.1(28.4-35.7) | 13.3(12.0-14.5) | 50.2(45.5-54.8) | 46.4(43.4-49.3) | 58.2(54.2-62.2) | 26.0(23.8-28.3) |
| Togo | 29.1(18.9-39.4) | 24.1(16.2-31.9) | NA | 47.9(38.4-57.4) | 8.0(4.4-11.6) | 14.3(11.0-17.6) | 11.1(8.1-14.1) |
| Uganda | 34.1(27.1-41.1) | 21.3(16.8-25.8) | 9.8(5.1-14.5) | 53.6(48.4-58.8) | 16.7(14.3-19.1) | 30.3(25.6-34.9) | 16.6(12.6-20.6) |
| United Republic of Tanzania | 11.8(9.4-14.2) | 17.5(13.1-22.0) | NA | 44.2(40.9-47.5) | 23.6(20.2-27.0) | NA | NA |
| Zambia | 37.5 (35.7-39.3) | 42.4 (40.6-44.3) | 18.0 (16.6-19.4) | 41.2 (39.4-43.0) | 31.7 (30.0-33.5) | 37.9 (36.1-39.8) | 26.9(25.2-28.5) |
| Zimbabwe | 43.7(37.4-50.0) | 41.0(33.1-48.9) | NA | 53.8(48.8-58.9) | 22.2(18.1-26.4) | 22.2(19.2-25.1) | 17.1(13.1-21.0) |
| **Americas** |  |  |  |  |  |  |  |
| Antigua and Barbuda | 14.2(11.8-16.6) | 14.1(11.8-16.4) | 13.0(11.3-14.6) | 29.2(26.6-31.9) | 34.6(32.0-37.3) | 27.4(25.0-29.8) | 8.1(6.5-9.6) |
| Argentina | 21.9(13.7-30.2) | 16.2(11.4-21.0) | NA | 31.8(24.5-39.1) | 24.4(18.7-30.0) | 12.8(9.5-16.0) | 8.7(3.9-13.4) |
| Bahamas | 14.3(8.5-20.1) | 17.3(12.5-22.1) | NA | 42.5(28.9-56.1) | 28.1(21.8-34.5) | NA | 16.0(2.7-29.4) |
| Barbados | NA | 17.3(14.5-20.1) | NA | 30.5(27.7-33.4) | 42.8(39.4-46.2) | NA | NA |
| Belize | NA | 15.9(13.3-18.5) | NA | 31.1(28.3-33.9) | 33.1(29.5-36.6) | NA | NA |
| Bolivia | 26.9(23.4-30.3) | 19.8(17.3-22.3) | 19.4(18.0-20.7) | 45.5(41.6-49.4) | 30.5(28.2-32.8) | 29.2(23.8-34.7) | 14.3(12.1-16.5) |
| Brazil | 7.8(6.3-9.3) | 10.0(8.4-11.6) | 9.7(8.3-11.0) | 35.2(32.5-37.9) | NA | NA | NA |
| Chile | NA | 11.4(9.9-13.0) | 18.6(17.3-20.0) | 27.9(26.4-29.4) | 37.3(35.8-38.8) | NA | NA |
| Costa Rica | 18.9(16.1-21.8) | 15.0(12.7-17.2) | 12.7(11.3-14.1) | 29.0(25.6-32.3) | 27.9(25.5-30.3) | 18.7(16.4-21.0) | 6.8(5.5-8.1) |
| Cuba | 17.4(14.2-20.6) | 10.9(9.3-12.6) | 18.5(17.2-19.8) | 42.3(39.8-44.8) | 17.5(15.5-19.5) | 12.8(11.3-14.3) | 7.6(6.2-9.0) |
| Dominican Republic | 16.5(9.5-23.5) | 36.7(30.1-43.2) | 63.1(57.0-69.1) | 44.8(38.3-51.3) | 32.1(27.6-36.6) | 21.7(18.9-24.6) | 9.8(6.7-12.9) |
| Ecuador | NA | 32.5(28.4-36.7) | NA | 42.8(39.1-46.4) | 35.5(32.2-38.7) | NA | NA |
| El Salvador | NA | 6.9(5.5-8.2) | NA | 33.3(30.6-36.0) | 32.8(30.3-35.3) | NA | NA |
| Grenada | NA | 12.4(10.0-14.7) | NA | 28.9(26.0-31.9) | 32.1(29.6-34.7) | NA | NA |
| Guatemala | 31.0(27.7-34.4) | 13.0(11.1-14.9) | 24.6(23.1-26.1) | 36.3(32.9-39.7) | 36.5(34.1-38.8) | 30.5(28.1-32.8) | 15.1(12.8-17.3) |
| Guyana | 15.0(11.2-18.8) | 26.8(19.9-33.7) | 12.6(10.8-14.5) | 48.7(42.5-54.9) | 27.4(23.9-31.0) | 41.8(36.9-46.6) | 14.4(11.0-17.9) |
| Honduras | NA | 27.9(24.6-31.2) | NA | 44.7(40.6-48.8) | 21.0(18.5-23.4) | NA | NA |
| Jamaica | 13.6(9.6-17.5) | 15.1 (13.2-17.0) | 18.3(14.5-22.1) | 34.3 (31.8-36.8) | 45.7 (43.0-48.3) | 46.1(40.6-51.7) | 16.2(11.4-21.1) |
| Mexico | 6.2(4.3-8.1) | 7.4(5.7-9.1) | 23.3(20.8-25.7) | 39.6(36.6-42.7) | 30.7(27.5-34.0) | 31.5(28.8-34.1) | 20.3(17.8-22.8) |
| Nicaragua | NA | 12.3(10.7-13.9) | 12.2(11.0-13.5) | 44.2(41.4-47.0) | 21.6(20.0-23.2) | 25.5(23.4-27.7) | 10.6(9.4-11.8) |
| Panama | 14.1(12.0-16.2) | 9.2(7.5-10.9) | 20.0(18.4-21.5) | 47.9(43.7-52.2) | 21.6(19.2-23.9) | 17.1(15.4-18.7) | 11.8(10.0-13.6) |
| Paraguay | 38.5(33.5-43.5) | 30.1(26.3-33.9) | 16.2(14.7-17.8) | 41.6(38.6-44.5) | 29.0(27.1-30.9) | 20.1(18.2-22.0) | NA |
| Peru | NA | 9.7(7.9-11.6) | NA | 34.1(31.7-36.5) | 23.3(21.0-25.5) | NA | NA |
| Saint Kitts and Nevis | 11.7(8.0-15.4) | 17.5(12.9-22.0) | 14.2(11.1-17.2) | 30.9(25.4-36.4) | 29.4(25.6-33.1) | 39.9(33.2-46.6) | 10.3(6.2-14.3) |
| Saint Lucia | 17.8(14.9-20.8) | 16.2(13.3-19.1) | 15.0(12.2-17.8) | 38.8(35.7-41.9) | 33.8(30.9-36.7) | 29.0(25.8-32.1) | 11.0(8.7-13.2) |
| Saint Vincent and the Grenadines | 16.2(12.9-19.6) | 16.4(13.7-19.0) | 18.1(15.7-20.6) | 35.2(32.1-38.3) | 40.3(36.4-44.1) | 41.3(38.6-43.9) | 7.9(6.5-9.3) |
| Suriname | 9.4(7.6-11.2) | 9.4(7.6-11.2) | NA | 26.7(23.7-29.6) | 44.8(41.6-48.1) | 41.0(37.7-44.3) | NA |
| Trinidad and Tobago | 14.3(11.4-17.3) | 20.2(17.4-23.1) | 16.0(14.5-17.5) | 30.0(25.6-34.3) | 31.7(28.7-34.7) | 34.9(31.9-37.9) | 9.8(8.2-11.4) |
| Uruguay | NA | 9.7(8.4-11.0) | 17.1(15.8-18.3) | 33.4(31.6-35.2) | 22.9(20.8-25.0) | NA | NA |
| Venezuela | NA | 31.1(27.2-34.9) | NA | 40.4(36.4-44.4) | 25.5(22.8-28.3) | 11.6(9.8-13.3) | 3.3(2.4-4.1) |
| **Eastern Mediterranean** |  |  |  |  |  |  |  |
| Afghanistan | 20.9(11.0-30.8) | 22.3(17.9-26.6) | 50.6(38.5-62.7) | 37.3(33.4-41.1) | 17.9(14.4-21.3) | 33.9(20.3-47.5) | 54.9(44.2-65.5) |
| Bahrain | 24.2(17.5-30.8) | 21.4(17.2-25.7) | NA | 35.4(30.5-40.2) | 23.0(20.4-25.5) | 29.6(26.4-32.8) | 21.0(18.0-24.0) |
| Djibouti | NA | 51.2(44.8-57.6) | NA | 58.3(53.4-63.3) | 32.5(27.6-37.4) | NA | NA |
| Egypt | 14.3(10.4-18.3) | 16.2(9.2-23.3) | 19.8(12.0-27.5) | 57.1(45.1-69.0) | 20.2(13.1-27.3) | 34.0(23.6-44.5) | 13.7(8.5-18.9) |
| Gaza Strip | NA | 20.1(13.7-26.6) | NA | 50.2(43.6-56.7) | 18.7(14.2-23.2) | NA | 16.0(9.9-22.2) |
| Iraq | 18.9(13.4-24.3) | 24.6(21.4-27.8) | NA | 54.7(50.8-58.5) | 23.4(19.2-27.7) | 25.8(20.6-31.1) | 13.4(9.7-17.1) |
| Jordan | 15.2(11.9-18.6) | 16.1(11.9-20.4) | NA | 35.7(31.5-40.0) | 24.4(21.0-27.8) | 36.6(33.5-39.6) | 19.4(15.7-23.2) |
| Kuwait | 12.9(10.0-15.7) | 14.1(11.9-16.2) | NA | 32.1(28.8-35.4) | 23.3(21.5-25.0) | 25.0(22.2-27.7) | 20.5(18.2-22.7) |
| Lebanon | 5.4(4.0-6.8) | 5.5(4.0-7.1) | NA | 21.7(18.8-24.6) | 18.6(16.7-20.5) | 20.9(17.5-24.2) | 17.2(15.2-19.3) |
| Libyan Arab Jamahiriya | 8.0(6.3-9.7) | 18.6(15.7-21.6) | 40.0(36.5-43.4) | NA | 13.5(10.6-16.3) | NA | 19.7(16.9-22.5) |
| Morocco | 11.6(9.5-13.7) | 15.1(12.7-17.5) | 76.1(74.0-78.2) | 44.1(41.1-47.1) | 18.2(14.7-21.6) | NA | 19.6(17.0-22.3) |
| Oman | 10.2(7.5-12.9) | 15.0(9.0-21.0) | 45.8(41.7-50.0) | 47.9(42.4-53.3) | 18.1(13.9-22.3) | 23.6(20.8-26.3) | 16.1(12.7-19.5) |
| Pakistan | 22.6(17.3-27.9) | 12.7(9.6-15.8) | 32.3(27.7-36.9) | 61.2(56.7-65.7) | 62.4(54.9-69.9) | 19.6(15.9-23.2) | 39.2(32.8-45.6) |
| Qatar | NA | 23.5(20.3-26.8) | NA | 36.3(31.5-41.1) | 22.7(20.1-25.3) | NA | NA |
| Saudi Arabia | 7.2(5.2-9.1) | 15.3(12.5-18.2) | 44.8(41.7-48.0) | NA | 14.9(12.1-17.8) | NA | 25.2(21.2-29.2) |
| Syrian Arab Republic | 10.5(7.8-13.2) | 19.0(13.5-24.6) | 55.7(51.2-60.1) | NA | 14.9(12.2-17.5) | NA | 18.5(14.8-22.2) |
| Tunisia | 5.7(3.9-7.4) | 12.5(10.2-14.8) | 44.7(41.2-48.3) | 43.3(39.7-46.8) | 20.6(17.9-23.3) | NA | 21.3(18.1-24.5) |
| United Arab Emirates | NA | 14.1(11.9-16.3) | 14.4(12.7-16.0) | 29.3(26.3-32.2) | 27.0(23.7-30.2) | 25.3(23.5-27.2) | NA |
| UNRWA GAZA | NA | 15.8(11.4-20.2) | NA | 44.5(39.6-49.5) | 19.8(15.8-23.8) | 31.9(26.2-37.7) | NA |
| UNRWA Jordan | NA | 19.8(16.1-23.6) | NA | 42.2(37.6-46.8) | 21.4(18.7-24.1) | 33.8(30.1-37.5) | NA |
| UNRWA Lebanon | NA | 14.7(11.6-17.9) | NA | 37.6(32.9-42.2) | 19.5(15.9-23.2) | 30.7(27.8-33.6) | NA |
| UNRWA West bank | NA | 12.9(9.5-16.4) | NA | 36.4(30.8-42.0) | 21.9(18.4-25.5) | 33.6(31.0-36.2) | NA |
| West BANK | NA | 17.4(14.5-20.4) | NA | 42.9(37.0-48.7) | 20.8(15.9-25.7) | NA | 19.4(14.6-24.2) |
| Yemen | NA | 18.3(13.0-23.6) | NA | 47.1(40.4-53.8) | 18.1(13.9-22.3) | NA | 13.7(10.4-17.0) |
| **Europe** |  |  |  |  |  |  |  |
| Albania | 15.8(13.5-18.1) | 10.8(9.0-12.7) | 47.5(45.4-49.6) | 26.3(24.1-28.6) | 24.6(22.3-26.8) | 22.1(19.9-24.2) | NA |
| Azerbaijan | 10.2(6.2-14.2) | 10.2(8.0-12.4) | 36.9(32.3-41.5) | 59.6(54.6-64.6) | 25.4(22.6-28.2) | 19.1(16.7-21.6) | 29.1(25.0-33.2) |
| Belarus | 4.8(3.7-5.9) | 8.4(6.9-9.8) | NA | 15.4(14.0-16.8) | 20.1(18.4-21.9) | NA | 3.5(2.5-4.4) |
| Bosnia and Herzegovina | 7.9(6.4-9.4) | 10.6(9.3-12.0) | NA | 21.1(18.6-23.6) | 45.6(43.8-47.4) | 16.6(15.2-18.0) | 15.0(13.7-16.3) |
| Bulgaria | NA | 14.8(11.4-18.2) | NA | 36.5(33.4-39.6) | 29.7(27.6-31.7) | NA | NA |
| Croatia (Hrvatska) | 7.1(5.4-8.7) | 9.2(7.1-11.3) | 41.9(39.6-44.2) | 18.6(16.1-21.1) | 43.6(40.2-47.0) | 26.7(23.0-30.4) | 21.1(18.1-24.2) |
| Cyprus | 12.0 (10.0-13.9) | 15.5 (13.3-17.8) | 22.4 (19.9-24.9) | NA | 25.2 (22.5-27.9) | 31.9 (29.0-34.7) | 25.4 (22.7-28.0) |
| Czech Republic | 7.0(6.0-8.0) | 10.0(8.7-11.4) | 21.2(19.5-23.0) | 11.6(10.1-13.1) | 39.8(36.9-42.7) | 33.8(30.1-37.5) | 13.0(10.0-15.9) |
| Finland | 5.2(4.3-6.1) | 22.2(20.6-23.7) | 34.4(32.8-36.0) | 19.8(18.3-21.4) | NA | NA | NA |
| Georgia | 11.4(8.8-13.9) | 9.3(6.8-11.7) | NA | 76.3(72.8-79.9) | 32.3(26.8-37.9) | 16.3(12.0-20.5) | 11.4(8.9-13.9) |
| Greece | NA | 6.2(5.0-7.5) | NA | 18.5(17.1-19.9) | 25.5(24.0-26.9) | NA | NA |
| Italy | 3.6(2.3-4.9) | 9.8(8.2-11.4) | NA | 10.9(9.1-12.7) | 40.8(37.5-44.2) | 21.0(18.2-23.7) | 14.0(11.6-16.4) |
| Kazakhstan | 8.5(5.0-12.1) | 8.8(6.4-11.3) | 44.5(37.4-51.5) | 24.0(19.6-28.5) | 12.4(9.6-15.2) | 10.9(7.7-14.1) | 12.3(7.6-17.0) |
| Kosovo | 8.6(7.1-10.1) | 11.3(10.5-12.1) | NA | 29.6(27.1-32.1) | 21.2(19.3-23.1) | 15.2(13.1-17.2) | 16.2(14.8-17.6) |
| Kyrgyzstan | NA | 12.6(10.8-14.3) | NA | 34.6(31.6-37.6) | 28.0(25.4-30.6) | NA | NA |
| Latvia | 7.6(6.0-9.2) | 9.7(8.0-11.5) | 39.9(38.1-41.7) | 17.9(15.6-20.1) | 26.8(24.8-28.7) | 23.9(21.7-26.2) | 6.9(4.9-9.0) |
| Lithuania | 13.6(11.5-15.8) | 17.3(15.1-19.5) | NA | 51.1(48.3-53.9) | 31.0(28.3-33.6) | 32.4(30.5-34.4) | 8.8(7.5-10.1) |
| Macedonia | 12.9(10.5-15.2) | 11.0(9.1-12.9) | NA | 28.6(25.8-31.4) | 23.0(21.5-24.5) | NA | 14.4(12.6-16.2) |
| Malta | NA | 8.2 (6.6-9.7) | NA | 16.7 (14.6-18.7) | 40.7 (37.9-43.4) | NA | NA |
| Montenegro | 9.2(7.1-11.3) | 11.7(10.3-13.0) | NA | 28.7(26.2-31.1) | 37.6(35.5-39.7) | 16.6(14.6-18.6) | 13.6(11.9-15.3) |
| Poland | 16.1(13.8-18.3) | 18.7(16.9-20.5) | 16.7(15.4-18.0) | 24.6(22.1-27.2) | 41.5(39.8-43.2) | 22.7(20.3-25.2) | 6.8(5.3-8.3) |
| Portugal | NA | 12.7(11.1-14.2) | NA | 22.9(20.4-25.4) | 30.6(29.3-31.9) | NA | 10.9(10.0-11.8) |
| Republic of Moldova | NA | 15.0(13.3-16.8) | NA | 28.7(26.0-31.3) | 29.6(27.4-31.9) | NA | NA |
| Romania | 11.9(9.5-14.3) | 17.9(15.5-20.2) | NA | 28.5(26.3-30.7) | 25.5(23.6-27.4) | 17.1(15.6-18.7) | 9.9(8.7-11.0) |
| Russian Federation | NA | 13.3(11.7-14.8) | NA | 15.7(14.0-17.5) | 24.8(20.6-28.9) | NA | NA |
| San Marino | 5.5(2.0-9.0) | 13.1(10.3-16.0) | 21.7(17.2-26.3) | 7.2(5.1-9.3) | 44.7(40.7-48.6) | 18.1(14.3-22.0) | 13.7(11.0-16.4) |
| Serbia | 8.0 (7.1-8.8) | 9.4 (8.4-10.3) | NA | 24.2 (22.8-25.5) | 53.6 (52.0-55.2) | NA | NA |
| Slovakia | 6.8(5.6-8.1) | 10.4(9.0-11.8) | 18.4(17.3-19.4) | 21.2(19.8-22.7) | 47.8(45.0-50.7) | 19.3(17.4-21.2) | 9.8(8.2-11.5) |
| Slovenia | 4.8(2.4-7.2) | 7.2(5.1-9.2) | 26.4(24.1-28.8) | 23.5(20.6-26.4) | 53.3(50.5-56.1) | 27.7(24.8-30.6) | 10.2(7.5-12.9) |
| Tajikistan | 9.4(7.3-11.5) | 17.6(10.9-24.4) | NA | 25.7(21.6-29.8) | 24.3(21.7-26.8) | NA | 51.8(44.3-59.3) |
| Turkey | 9.7(8.9-10.5) | 8.5(8.0-9.0) | 70.1(67.5-72.6) | 38.3(37.4-39.2) | 27.7(26.8-28.5) | 32.7(31.8-33.5) | 16.4(14.2-18.6) |
| Turkmenistan | NA | 2.9(1.9-3.9) | NA | 6.7(4.3-9.2) | 7.4(3.3-11.5) | NA | NA |
| Ukraine | 10.8(7.7-13.8) | 15.2(11.6-18.8) | 46.3(41.7-51.0) | 27.2(24.1-30.4) | 25.7(20.4-30.9) | 32.8(29.4-36.2) | 8.9(7.0-10.9) |
| **South-East Asia** |  |  |  |  |  |  |  |
| Bangladesh | NA | 9.2(5.1-13.4) | NA | 45.0(40.0-50.1) | 33.1(23.1-43.0) | NA | NA |
| Bhutan | NA | 10.6(8.7-12.4) | NA | 30.5(27.6-33.3) | 16.4(14.6-18.2) | NA | NA |
| Indonesia | 7.5(6.4-8.7) | 5.7(4.6-6.9) | 28.1(26.0-30.2) | 20.4(18.2-22.5) | 12.8(11.6-13.9) | 19.5(15.8-23.2) | 6.0(4.6-7.3) |
| Maldives | 5.2(3.9-6.5) | 14.5(12.5-16.4) | NA | 29.4(27.1-31.7) | 18.5(16.6-20.3) | 36.8(32.8-40.8) | 14.9(12.8-16.9) |
| Myanmar | 6.6(5.2-7.9) | 4.7(3.5-5.9) | 49.5(46.3-52.7) | 28.7(25.6-31.8) | 25.8(20.5-31.1) | 40.5(36.2-44.7) | 37.5(32.3-42.7) |
| Nepal | 10.6(7.7-13.6) | 17.9(14.8-21.1) | 32.9(28.3-37.6) | 29.9(26.2-33.6) | 53.7(49.3-58.0) | 55.8(50.6-61.1) | 53.0(49.6-56.4) |
| Sri Lanka | 6.6(4.6-8.6) | 6.5(5.0-8.0) | 12.9(10.6-15.1) | 63.3(57.9-68.7) | 24.3(18.7-29.8) | 53.8(51.5-56.0) | 13.2(11.6-14.8) |
| Thailand | NA | 9.4(7.4-11.4) | NA | 47.7(43.1-52.2) | 28.1(26.0-30.2) | 23.9(20.2-27.6) | 11.9(9.2-14.7) |
| Timor-Leste | 67.8(60.0-75.6) | 35.6(32.0-39.2) | NA | 53.3(50.3-56.4) | 39.1(36.3-41.8) | NA | NA |
| **Western Pacific** |  |  |  |  |  |  |  |
| Brunei Darussalam | 5.3(3.6-7.0) | 6.0(4.2-7.8) | 31.1(28.3-34.0) | 15.4(12.8-18.0) | 10.6(7.6-13.6) | 19.0(16.7-21.3) | 5.2(3.7-6.7) |
| Cambodia | 6.9(5.2-8.7) | 6.2(5.2-7.3) | 88.8(86.4-91.2) | 24.1(21.4-26.9) | 10.8(8.6-12.9) | 9.4(7.1-11.7) | 11.8(9.1-14.4) |
| Cook Islands | 21.0 (17.3-24.6) | 26.1 (22.2-30.1) | 21.6 (17.9-25.3) | 36.3 (32.0-40.6) | 29.7 (25.5-33.9) | 27.4  (23.4-31.4) | 10.1 (7.4-12.9) |
| Fiji | 21.8(16.9-26.7) | 19.2(15.8-22.5) | 18.1(14.6-21.5) | 40.0(35.4-44.6) | 27.2(24.1-30.3) | 40.3(35.3-45.4) | 19.4(15.6-23.1) |
| Guam | 15.8(12.3-19.2) | 18.3(16.2-20.4) | 16.6(13.5-19.6) | 28.5(25.5-31.5) | 26.5(24.3-28.7) | 31.2(27.6-34.7) | 10.5(7.9-13.1) |
| Kiribati | 16.3(13.7-18.9) | 13.7(11.5-15.9) | 24.6(22.3-26.9) | 44.1(41.3-46.8) | 22.8(20.5-25.2) | NA | NA |
| Laos | 12.2(10.1-14.2) | 14.5(11.3-17.8) | 31.0(27.6-34.4) | 68.6(65.3-72.0) | 41.8(39.4-44.2) | 35.2(31.9-38.6) | 26.4(23.6-29.3) |
| Macao (China) | 3.8(2.6-5.0) | 4.3(3.2-5.4) | 13.2(10.8-15.6) | 15.4(12.8-18.0) | 7.9(3.6-12.3) | 6.3(5.0-7.6) | 6.5(5.3-7.8) |
| Marshall Islands | 50.8(47.4-54.3) | 41.3(37.9-44.7) | NA | 55.3(52.5-58.2) | 33.2(30.8-35.6) | 35.5(33.0-38.0) | 30.1(27.7-32.5) |
| Micronesia | 40.6(38.1-43.1) | 38.0(35.6-40.4) | 26.8(25.4-28.3) | 48.0(45.9-50.0) | 32.2(30.7-33.8) | 50.7(48.8-52.5) | 27.7(26.0-29.4) |
| Mongolia | 8.1(6.6-9.5) | 11.6(9.9-13.3) | NA | 60.0(56.8-63.3) | 6.8(5.4-8.2) | 18.7(16.5-20.9) | 3.4(2.6-4.3) |
| New Caledonia | 3.5(1.8-5.3) | 9.8(7.1-12.6) | 57.6(52.2-63.0) | 19.5(15.9-23.1) | 47.9(41.9-53.9) | 34.3(27.8-40.7) | 17.7(12.9-22.4) |
| New Zealand | 7.2(4.5-9.9) | 8.7(6.2-11.1) | NA | 15.9(11.6-20.2) | NA | NA | NA |
| Niue | 24.9(15.7-34.1) | 23.4(16.3-30.6) | 16.6(9.4-23.8) | 24.3(17.2-31.5) | 40.2(26.2-54.1) | 32.6(24.9-40.2) | 13.7(5.3-22.0) |
| Northern Mariana Islands | NA | 13.6(11.8-15.5) | NA | 27.9(24.7-31.1) | 28.4(24.5-32.3) | 29.4(24.3-34.5) | 8.7(7.2-10.1) |
| Palau | 14.5 (12.4-16.6) | 15.1(12.5-17.6) | 19.1(16.7-21.4) | 27.4(24.3-30.4) | 26.3(23.1-29.5) | NA | NA |
| Papua New Guinea | 31.4(24.4-38.4) | 32.8(26.3-39.3) | 16.7(14.5-18.9) | 49.0(44.3-53.6) | 25.5(23.6-27.4) | 45.8(40.6-51.1) | 16.8(14.0-19.7) |
| Philippines | 19.6(17.6-21.6) | 16.3(14.5-18.0) | 16.9(15.6-18.2) | 41.1(39.1-43.1) | 16.9(15.4-18.3) | 33.0(31.0-34.9) | 14.7(13.0-16.4) |
| Samoa | 26.1(20.9-31.3) | 10.0(8.0-12.0) | NA | 70.5(64.3-76.7) | 34.1(31.3-36.9) | NA | NA |
| South Korea | NA | 3.4(2.9-3.9) | NA | 27.1(24.9-29.4) | 9.5(8.4-10.5) | NA | NA |
| Tokelau | 23.2(10.2-36.2) | 21.8(12.2-31.3) | 29.4(19.5-39.3) | 35.7(24.1-47.2) | 42.1(28.3-55.9) | 42.1(28.7-55.5) | 29.6(14.4-44.7) |
| Tonga | NA | 31.3(27.8-34.8) | 25.8(22.6-29.0) | 57.0(52.3-61.7) | 36.1(32.4-39.8) | 61.5(57.7-65.3) | 27.5(23.4-31.6) |
| Tuvalu | 12.9(9.1-16.6) | 31.4(24.9-38.0) | 43.5(38.2-48.9) | 44.2(38.7-49.8) | 24.6(19.6-29.6) | NA | NA |
| Vanuatu | NA | 45.8(39.1-52.5) | 12.9(9.5-16.2) | 47.7(42.9-52.6) | 37.2(32.7-41.7) | 67.7(64.1-71.2) | 26.4(21.1-31.7) |
| Viet Nam | 6.7(5.2-8.2) | 5.1(4.0-6.3) | 89.3(86.8-91.8) | 24.3(21.8-26.7) | 26.9(24.0-29.8) | 19.9(17.2-22.6) | 7.4(6.0-8.8) |

Abbreviation: NA, not available.

Data are presented as %(95%CI).

**Table S3 Proportions of incorrect beliefs and attitudes toward tobacco smoking among adolescents aged 12–16 years who are current smokers, by country/territory.**

| **Country/territory** | **Tobacco smoking is not harmful** | **Secondhand smoke exposure is not harmful** | **It is safe to smoke one or two years then quit** | **Once someone smoke, it is easy to quit** | **Tobacco smoking helps people feel more comfortable** | **Tobacco smoking makes more friends** | **Tobacco smoking is more attractive** |
| --- | --- | --- | --- | --- | --- | --- | --- |
| **Africa** |  |  |  |  |  |  |  |
| Algeria | NA | 15.1 (10.8-19.3) | NA | 37.6 (33.3-42.0) | 44.4 (39.3-49.5) | NA | NA |
| Angola | 76.2 (65.1-87.3) | 65.4 (50.7-80.1) | NA | 91.5 (87.0-96.0) | NA | NA | 75.2 (61.7-88.6) |
| Cameroon | NA | 37.6 (23.8-51.5) | NA | 50.0 (37.0-63.0) | 26.6 (20.6-32.6) | NA | NA |
| Chad | NA | 61.8 (52.6-71.0) | NA | NA | 45.7 (36.0-55.4) | 42.1 (32.6-51.6) | 46.6 (36.8-56.4) |
| Comoros | NA | 46.7 (39.1-54.3) | NA | 52.7 (44.9-60.5) | 31.9 (23.9-39.9) | NA | NA |
| Congo | NA | 50.1 (39.4-60.8) | NA | 64.4 (57.1-71.6) | 35.4 (26.4-44.5) | 39.6 (32.1-47.1) | 38.5 (27.7-49.4) |
| Gabon | NA | 15.7 (6.5-24.8) | NA | 27.9 (13.6-42.2) | 29.5 (20.8-38.3) | NA | NA |
| Gambia | 42.6 (36.4-48.8) | 36.3 (30.9-41.6) | 22.2 (18.3-26.0) | 60.3 (54.8-65.9) | 33.0 (28.1-37.9) | 28.7 (22.7-34.7) | 16.8 (13.2-20.5) |
| Ghana | NA | 33.1 (23.0-43.2) | NA | 49.7 (37.9-61.4) | 29.9 (20.7-39.1) | NA | NA |
| Kenya | NA | 16.0 (9.7-22.4) | NA | 47.3 (40.1-54.4) | 25.6 (15.6-35.6) | NA | NA |
| Madagascar | NA | 11.0 (5.0-17.1) | NA | 59.4 (44.8-74.0) | 29.5 (11.9-47.2) | NA | NA |
| Mauritania | 37.2 (29.7-44.8) | 46.9 (38.3-55.5) | 42.4 (30.6-54.1) | 71.5 (65.3-77.6) | 34.7 (20.4-49.1) | 46.1 (34.9-57.2) | 42.1 (31.2-53.0) |
| Mauritius | 19.4 (15.7-23.1) | 26.5 (18.0-34.9) | 35.0 (29.9-40.2) | 31.6 (27.0-36.3) | 35.9 (29.5-42.3) | 46.4 (41.4-51.3) | 21.2 (17.0-25.5) |
| Mozambique | NA | 50.6 (41.0-60.2) | NA | 57.6 (46.8-68.4) | 36.7 (25.4-48.0) | NA | NA |
| Sao Tome and Principe | 22.2 (19.8-24.6) | 24.9 (22.5-27.4) | 42.6 (39.8-45.4) | 54.5 (51.6-57.3) | 35.1 (32.3-37.9) | 26.4 (23.9-28.9) | 34.0 (31.3-36.7) |
| Senegal | NA | 48.2 (33.7-62.7) | NA | 39.7 (27.4-52.0) | 27.3 (15.9-38.6) | NA | NA |
| Seychelles | NA | 39.2 (33.4-45.0) | NA | 41.3 (36.2-46.4) | 42.6 (37.8-47.3) | NA | 18.5 (14.6-22.3) |
| Sierra Leone | 25.7 (15.0-36.4) | 32.1 (15.4-48.8) | NA | 58.1 (46.1-70.1) | 45.5 (22.9-68.2) | NA | NA |
| South Africa | 32.2 (27.7-36.7) | 36.2 (32.0-40.3) | 18.6 (15.9-21.2) | 54.0 (49.8-58.2) | 48.8 (45.1-52.5) | 60.5 (56.5-64.4) | 29.3 (25.6-33.0) |
| Togo | 39.5 (24.7-54.3) | 27.8 (15.7-39.9) | NA | 49.3 (34.8-63.7) | 15.2 (6.4-24.0) | 25.1 (10.8-39.4) | 19.4 (9.7-29.1) |
| Uganda | 35.6 (25.5-45.7) | 21.8 (12.7-30.8) | 19.8 (10.6-28.9) | 55.6 (44.2-67.0) | 20.0 (11.1-28.9) | 40.6 (31.6-49.6) | 22.8 (15.5-30.1) |
| United Republic of Tanzania | 30.9 (11.3-50.5) | 26.6 (9.2-44.1) | NA | 16.2 (2.9-29.5) | 33.0 (12.9-53.1) | NA | NA |
| Zambia | 48.1 (43.6-52.7) | 51.9 (47.3-56.4) | 29.2 (25.0-33.4) | 45.1 (40.5-49.7) | 35.8 (31.3-40.3) | 45.5 (41.0-50.1) | 37.5 (33.0-41.9) |
| Zimbabwe | 53.9 (39.8-67.9) | 46.1 (31.2-61.1) | NA | 45.8 (33.7-57.9) | 29.9 (12.5-47.3) | 21.8 (12.3-31.4) | 22.9 (12.1-33.6) |
| **Americas** |  |  |  |  |  |  |  |
| Antigua and Barbuda | 19.8 (11.3-28.2) | 16.9 (9.6-24.3) | 23.4 (15.1-31.6) | 33.9 (25.4-42.4) | 35.4 (24.7-46.2) | 26.9 (18.4-35.5) | 15.2 (7.2-23.2) |
| Argentina | 21.9 (6.2-37.5) | 17.6 (7.2-28.1) | NA | 19.4 (6.1-32.7) | 27.8 (13.7-41.8) | 6.2 (-1.0-13.5) | 10.6 (2.1-19.1) |
| Bahamas | 19.1 (11.8-26.3) | 26.5 (19.7-33.3) | NA | 48.2 (37.8-58.5) | 37.1 (26.0-48.2) | NA | 14.3 (7.0-21.7) |
| Barbados | NA | 29.1 (21.0-37.3) | NA | 42.3 (36.0-48.6) | 50.6 (43.2-57.9) | NA | NA |
| Belize | NA | 29.3 (21.9-36.7) | NA | 39.5 (29.2-49.7) | 49.4 (37.4-61.3) | NA | NA |
| Bolivia | 32.3 (24.6-40.0) | 27.0 (21.5-32.6) | 33.4 (29.7-37.2) | 46.6 (41.0-52.1) | 40.1 (35.3-44.9) | 32.3 (19.8-44.7) | 17.5 (13.3-21.6) |
| Brazil | 10.1 (6.6-13.6) | 18.3 (14.1-22.6) | 20.1 (16.3-24.0) | 39.4 (34.1-44.7) | NA | NA | NA |
| Chile | NA | 20.3 (15.8-24.9) | 31.3 (28.8-33.9) | 38.4 (34.8-42.0) | 44.8 (41.1-48.5) | NA | NA |
| Costa Rica | 23.1 (17.1-29.0) | 21.0 (14.4-27.6) | 26.0 (19.0-33.0) | 37.2 (30.0-44.5) | 46.8 (37.1-56.5) | 25.3 (19.1-31.6) | 14.2 (8.5-19.8) |
| Cuba | 25.6 (18.4-32.9) | 15.0 (9.9-20.2) | 37.0 (30.9-43.0) | 42.7 (36.5-48.9) | 30.2 (22.0-38.4) | 20.4 (15.6-25.2) | 11.7 (6.1-17.2) |
| Dominican Republic | 14.7 (4.5-25.0) | 37.0 (15.5-58.5) | 61.6 (51.6-71.7) | 37.4 (11.3-63.5) | 56.2 (28.7-83.6) | 24.2 (15.0-33.4) | 10.2 (5.4-15.1) |
| Ecuador | NA | 37.6 (31.1-44.0) | NA | 46.7 (41.3-52.1) | 44.8 (40.0-49.5) | NA | NA |
| El Salvador | NA | 14.8 (9.6-19.9) | NA | 37.5 (30.8-44.2) | 48.0 (41.6-54.3) | NA | NA |
| Grenada | NA | 22.6 (14.4-30.7) | NA | 42.9 (34.2-51.6) | 39.9 (29.9-49.9) | NA | NA |
| Guatemala | 35.4 (29.7-41.0) | 20.2 (16.1-24.4) | 41.8 (37.3-46.3) | 40.1 (35.0-45.3) | 50.2 (44.9-55.5) | 38.3 (33.3-43.2) | 22.2 (18.2-26.2) |
| Guyana | 17.6 (12.4-22.8) | 41.4 (32.2-50.7) | 17.4 (13.5-21.3) | 58.7 (47.7-69.6) | 38.1 (25.9-50.4) | 53.4 (45.9-60.8) | 22.4 (16.8-28.1) |
| Honduras | NA | 41.4 (31.1-51.7) | NA | 48.5 (38.6-58.4) | 40.6 (35.2-45.9) | NA | NA |
| Jamaica | 20.7 (15.8-25.6) | 18.8 (13.4-24.2) | 30.9 (26.7-35.2) | 41.1 (34.2-47.9) | 60.2 (53.3-67.1) | 56.1 (47.6-64.6) | 29.6 (21.8-37.4) |
| Mexico | 9.3 (5.4-13.1) | 12.3 (7.6-16.9) | 38.4 (33.2-43.6) | 47.2 (42.0-52.4) | 38.8 (34.4-43.2) | 34.6 (29.3-39.8) | 35.8 (30.8-40.7) |
| Nicaragua | NA | 20.3 (15.0-25.6) | 25.3 (21.5-29.0) | 51.4 (46.2-56.5) | 31.5 (27.7-35.3) | 36.1 (31.5-40.6) | 15.0 (12.6-17.3) |
| Panama | 25.4 (20.4-30.5) | 16.5 (6.7-26.2) | 37.2 (32.0-42.3) | 64.1 (53.1-75.0) | 34.8 (25.3-44.2) | 31.7 (23.7-39.7) | 24.8 (19.9-29.7) |
| Paraguay | 44.9 (37.3-52.4) | 40.7 (34.0-47.5) | 22.3 (18.1-26.5) | 59.0 (49.7-68.2) | 35.2 (30.2-40.1) | 24.8 (18.0-31.6) | NA |
| Peru | NA | 14.8 (9.5-20.2) | NA | 42.3 (36.3-48.4) | 40.1 (31.8-48.5) | NA | NA |
| Saint Kitts and Nevis | 30.8 (19.1-42.6) | 30.0 (16.8-43.2) | 21.2 (7.6-34.8) | 45.9 (35.3-56.5) | 31.6 (21.5-41.8) | 55.1 (39.7-70.4) | 29.6 (14.4-44.7) |
| Saint Lucia | 29.3 (18.6-39.9) | 21.6 (14.3-28.9) | 22.7 (17.1-28.3) | 44.7 (33.6-55.8) | 37.5 (28.5-46.5) | 41.9 (32.1-51.7) | 23.0 (14.4-31.6) |
| Saint Vincent and the Grenadines | 23.0 (14.3-31.7) | 25.0 (15.4-34.7) | 25.6 (20.0-31.1) | 44.3 (36.5-52.1) | 47.3 (36.7-58.0) | 47.0 (42.0-52.0) | 9.1 (2.9-15.3) |
| Suriname | 11.1 (6.1-16.1) | 13.3 (7.2-19.3) | NA | 36.1 (30.2-42.0) | 42.4 (34.8-49.9) | 46.8 (35.9-57.8) | NA |
| Trinidad and Tobago | 22.3 (16.0-28.6) | 33.4 (25.4-41.5) | 30.9 (24.9-36.8) | 37.0 (28.1-45.9) | 51.2 (45.3-57.1) | 39.5 (32.6-46.4) | 18.7 (14.8-22.6) |
| Uruguay | NA | 15.8 (11.6-20.0) | 29.1 (23.6-34.5) | 39.5 (34.9-44.1) | 36.6 (30.4-42.9) | NA | NA |
| Venezuela | NA | 33.4 (21.9-44.8) | NA | 41.4 (27.9-54.8) | 39.4 (25.1-53.8) | NA | NA |
| **Eastern Mediterranean** |  |  |  |  |  |  |  |
| Afghanistan | 35.3 (18.6-52.0) | 24.9 (19.4-30.4) | 47.8 (37.0-58.5) | 46.4 (36.4-56.3) | 32.1 (21.8-42.3) | 46.4 (28.7-64.0) | 48.6 (30.8-66.3) |
| Bahrain | 41.4 (34.3-48.4) | 37.0 (30.8-43.1) | NA | 48.0 (42.5-53.5) | 35.5 (27.6-43.4) | 40.3 (33.6-47.1) | 34.2 (28.9-39.4) |
| Djibouti | NA | 63.4 (54.1-72.7) | NA | 52.2 (38.8-65.5) | 41.4 (29.3-53.4) | NA | NA |
| Egypt | 33.3 (8.6-58.1) | 17.1 (8.4-25.8) | 21.5 (8.6-34.4) | 55.0 (30.6-79.4) | 50.6 (32.9-68.3) | 37.5 (17.6-57.5) | 32.0 (11.0-53.0) |
| Gaza Strip | NA | 28.9 (21.5-36.2) | NA | 44.1 (34.6-53.5) | 27.6 (19.5-35.8) | NA | 33.8 (25.7-41.9) |
| Iraq | 33.7 (24.6-42.7) | 31.6 (25.2-38.0) | NA | 48.6 (40.3-56.9) | 40.0 (28.7-51.3) | 38.2 (33.5-42.8) | 24.0 (15.7-32.2) |
| Jordan | 23.1 (17.4-28.9) | 23.0 (16.6-29.4) | NA | 37.5 (30.6-44.3) | 37.3 (28.8-45.9) | 40.0 (32.7-47.4) | 30.7 (22.6-38.8) |
| Kuwait | 24.2 (16.9-31.4) | 25.2 (18.0-32.5) | NA | 39.5 (32.9-46.0) | 44.5 (37.9-51.0) | 36.8 (29.3-44.3) | 33.5 (27.0-40.1) |
| Lebanon | 6.4 (4.0-8.8) | 8.3 (5.4-11.2) | NA | 19.0 (16.8-21.2) | 23.3 (19.3-27.3) | 27.9 (21.1-34.7) | 24.4 (20.7-28.1) |
| Libyan Arab Jamahiriya | 20.6 (11.3-29.9) | 24.8 (16.8-32.7) | 48.0 (37.5-58.4) | NA | 36.9 (27.6-46.2) | NA | 37.3 (25.2-49.3) |
| Morocco | 31.0 (16.6-45.3) | 31.2 (20.0-42.4) | 69.6 (60.7-78.6) | 49.6 (38.5-60.7) | 36.5 (24.0-49.0) | NA | 30.1 (23.4-36.8) |
| Oman | 40.7 (20.5-60.8) | 38.9 (25.3-52.5) | 44.7 (29.8-59.6) | 58.8 (47.7-69.9) | 39.2 (25.5-52.9) | 35.3 (24.0-46.6) | 37.6 (26.4-48.9) |
| Pakistan | 42.2 (30.6-53.8) | 23.3 (15.4-31.2) | 33.7 (24.3-43.1) | 63.8 (54.9-72.7) | 51.1 (40.4-61.8) | 38.3 (24.7-51.9) | 39.4 (28.7-50.0) |
| Qatar | NA | 37.8 (30.0-45.6) | NA | 45.4 (37.1-53.8) | 44.9 (38.3-51.4) | NA | NA |
| Saudi Arabia | 10.7 (6.5-14.9) | 27.0 (19.4-34.6) | 62.4 (53.3-71.6) | NA | 28.4 (20.4-36.3) | NA | 37.9 (30.3-45.6) |
| Syrian Arab Republic | 13.4 (7.6-19.1) | 19.4 (13.1-25.6) | 60.7 (52.3-69.2) | NA | 24.5 (18.3-30.7) | NA | 26.2 (20.5-31.9) |
| Tunisia | 8.6 (4.0-13.3) | 19.2 (12.9-25.5) | 57.9 (49.5-66.4) | 46.0 (38.1-53.9) | 38.5 (30.5-46.5) | NA | 33.0 (25.8-40.2) |
| United Arab Emirates | NA | 21.3 (16.9-25.7) | 39.2 (33.2-45.2) | 35.3 (28.7-41.8) | 49.5 (41.5-57.6) | 42.0 (36.0-48.0) | NA |
| UNRWA GAZA | NA | 22.1 (11.8-32.4) | NA | 45.5 (36.7-54.4) | 39.6 (30.8-48.3) | 39.8 (31.6-48.0) | NA |
| UNRWA Jordan | NA | 27.1 (17.0-37.3) | NA | 45.1 (38.0-52.2) | 35.6 (29.4-41.7) | 34.8 (26.4-43.2) | NA |
| UNRWA Lebanon | NA | 20.1 (15.4-24.8) | NA | 35.5 (30.2-40.8) | 28.2 (22.5-33.9) | 35.3 (29.7-41.0) | NA |
| UNRWA West bank | NA | 15.2 (11.3-19.1) | NA | 37.6 (29.1-46.1) | 29.7 (24.8-34.6) | 39.4 (33.9-44.9) | NA |
| West BANK | NA | 27.1 (21.7-32.4) | NA | 41.9 (34.0-49.7) | 31.6 (18.1-45.0) | NA | 34.9 (26.3-43.5) |
| Yemen | NA | 24.7 (13.6-35.7) | NA | 60.3 (50.6-70.1) | 28.0 (18.1-37.9) | NA | 25.6 (17.4-33.9) |
| **Europe** |  |  |  |  |  |  |  |
| Albania | 34.2 (27.0-41.3) | 20.5 (13.5-27.6) | 50.8 (44.3-57.3) | 35.4 (29.8-40.9) | 42.6 (36.6-48.7) | 34.3 (26.0-42.7) | NA |
| Azerbaijan | 33.5 (20.8-46.2) | 31.2 (20.7-41.8) | 36.8 (25.7-47.8) | 63.7 (53.1-74.3) | 34.4 (23.0-45.7) | 30.7 (22.0-39.3) | 33.9 (23.7-44.1) |
| Belarus | 10.0 (5.8-14.2) | 14.5 (9.7-19.4) | NA | 26.7 (19.7-33.8) | 25.1 (15.8-34.4) | NA | 8.6 (3.0-14.3) |
| Bosnia and Herzegovina | 13.1 (10.0-16.2) | 17.3 (14.0-20.5) | NA | 28.6 (24.0-33.2) | 52.7 (49.5-55.9) | 22.1 (18.2-26.0) | 22.9 (19.2-26.5) |
| Bulgaria | NA | 21.3 (16.5-26.1) | NA | 42.8 (39.8-45.8) | 34.8 (31.0-38.6) | NA | NA |
| Croatia (Hrvatska) | 11.9 (7.7-16.2) | 16.6 (11.7-21.6) | 45.1 (41.0-49.3) | 29.3 (24.3-34.4) | 51.7 (47.1-56.4) | 27.3 (23.1-31.6) | 24.0 (19.8-28.3) |
| Cyprus | 18.9 (13.4-24.4) | 33.8 (27.2-40.4) | 45.3 (38.3-52.2) | NA | 30.7 (24.1-37.3) | 30.3 (23.9-36.8) | 33.8 (27.2-40.4) |
| Czech Republic | 10.8 (8.4-13.2) | 20.4 (17.2-23.6) | 28.0 (25.1-30.9) | 18.3 (15.8-20.8) | 47.8 (43.1-52.5) | 33.8 (28.7-38.8) | 16.8 (12.1-21.5) |
| Finland | 6.7 (4.8-8.5) | 28.2 (25.4-30.9) | 36.2 (32.9-39.4) | 27.0 (24.0-30.0) | NA | NA | NA |
| Georgia | 24.0 (13.8-34.2) | 17.6 (9.8-25.3) | NA | 59.6 (50.1-69.0) | 40.7 (33.7-47.6) | 26.6 (19.1-34.0) | 23.7 (14.4-33.0) |
| Greece | NA | 12.6 (9.4-15.7) | NA | 25.5 (20.7-30.3) | 38.2 (33.8-42.7) | NA | NA |
| Italy | 3.8 (0.5-7.2) | 17.8 (13.5-22.0) | NA | 18.5 (13.8-23.1) | 42.2 (36.0-48.4) | 17.4 (12.1-22.7) | 14.0 (9.4-18.7) |
| Kazakhstan | 21.1 (6.0-36.3) | 30.9 (10.2-51.6) | 46.2 (28.5-63.9) | 26.3 (9.4-43.2) | 42.0 (20.4-63.6) | 33.3 (13.4-53.2) | 25.2 (10.7-39.8) |
| Kosovo | 19.9 (14.6-25.2) | 20.4 (15.8-25.0) | NA | 34.4 (28.9-39.8) | 34.3 (27.9-40.8) | 28.3 (20.8-35.7) | 34.3 (27.1-41.5) |
| Kyrgyzstan | NA | 35.8 (28.2-43.4) | NA | 42.3 (32.5-52.1) | 38.8 (32.0-45.6) | NA | NA |
| Latvia | 12.6 (8.6-16.5) | 15.3 (12.4-18.3) | 44.5 (41.5-47.5) | 24.3 (19.9-28.7) | 37.5 (32.7-42.3) | 28.0 (23.9-32.1) | 9.2 (6.1-12.3) |
| Lithuania | 21.9 (18.2-25.5) | 25.2 (20.9-29.5) | NA | 61.2 (57.6-64.7) | 41.6 (37.6-45.5) | 32.9 (28.1-37.7) | 14.8 (11.9-17.6) |
| Macedonia | 22.2 (18.2-26.2) | 24.4 (19.5-29.3) | NA | 35.9 (31.4-40.4) | 31.6 (26.9-36.4) | NA | 18.8 (14.5-23.0) |
| Malta | NA | 14.3 (5.9-22.7) | NA | 32.9 (21.6-44.1) | 56.5 (44.5-68.5) | NA | NA |
| Montenegro | 18.0 (12.8-23.1) | 26.9 (21.6-32.3) | NA | 40.3 (33.3-47.4) | 51.5 (45.3-57.8) | 25.7 (20.2-31.2) | 21.1 (15.0-27.3) |
| Poland | 25.2 (21.9-28.5) | 26.2 (22.5-29.9) | 27.5 (24.1-31.0) | 34.1 (30.1-38.1) | 51.4 (47.2-55.5) | 22.0 (18.7-25.2) | 8.9 (6.8-10.9) |
| Portugal | NA | 29.8 (25.4-34.2) | NA | 34.0 (29.3-38.7) | 42.8 (38.0-47.6) | NA | 22.1 (19.2-25.0) |
| Republic of Moldova | NA | 22.6 (17.8-27.5) | NA | 30.6 (25.6-35.6) | 36.9 (30.0-43.9) | NA | NA |
| Romania | 15.6 (10.2-21.1) | 26.3 (21.1-31.5) | NA | 35.3 (29.8-40.9) | 35.8 (31.5-40.1) | 21.2 (17.1-25.3) | 11.5 (8.7-14.3) |
| Russian Federation | NA | 26.6 (21.2-32.1) | NA | 28.8 (22.1-35.6) | 40.4 (30.0-50.8) | NA | NA |
| San Marino | 2.0 (-2.1-6.2) | 17.7 (7.1-28.3) | 32.4 (19.0-45.8) | 13.0 (5.1-20.9) | 37.6 (24.9-50.3) | 20.5 (6.4-34.5) | 13.0 (4.1-21.9) |
| Serbia | 13.5 (10.5-16.4) | 17.3 (14.1-20.6) | NA | 34.5 (30.4-38.6) | 64.4 (60.2-68.5) | NA | NA |
| Slovakia | 9.2 (7.2-11.3) | 19.0 (14.7-23.2) | 30.0 (26.4-33.6) | 30.0 (27.0-33.1) | 59.2 (53.3-65.1) | 24.0 (19.2-28.9) | 14.1 (11.0-17.1) |
| Slovenia | 8.4 (2.8-14.1) | 13.5 (6.7-20.2) | 42.5 (36.7-48.3) | 37.4 (33.5-41.2) | 67.8 (61.1-74.6) | 27.4 (19.6-35.2) | 18.7 (12.3-25.2) |
| Tajikistan | 19.9 (6.7-33.1) | 42.7 (22.6-62.7) | NA | 33.8 (14.6-53.0) | 35.8 (13.4-58.2) | NA | 69.0 (51.7-86.3) |
| Turkey | 19.4 (17.3-21.4) | 18.5 (17.0-19.9) | 70.1 (67.5-72.7) | 44.3 (42.2-46.3) | 38.9 (37.4-40.5) | 40.9 (39.1-42.6) | 15.9 (13.7-18.2) |
| Ukraine | 6.8 (2.7-10.9) | 24.6 (16.4-32.8) | 42.5 (31.7-53.3) | 36.2 (22.9-49.5) | 41.8 (28.0-55.6) | 39.4 (32.8-46.0) | 16.4 (10.0-22.8) |
| **South-East Asia** |  |  |  |  |  |  |  |
| Bangladesh | NA | 9.2 (-5.1-23.4) | NA | 30.3 (3.5-57.1) | 30.7 (3.6-57.9) | NA | NA |
| Bhutan | NA | 15.1 (11.9-18.3) | NA | 32.9 (28.5-37.2) | 24.0 (20.0-28.1) | NA | NA |
| Indonesia | 13.9 (11.4-16.3) | 10.3 (7.8-12.7) | 51.1 (47.6-54.7) | 29.1 (25.4-32.9) | 22.9 (20.0-25.7) | 36.8 (30.0-43.5) | 16.0 (13.0-18.9) |
| Maldives | 14.3 (8.6-20.1) | 26.9 (19.8-33.9) | NA | 35.7 (28.2-43.2) | 35.9 (27.5-44.3) | 55.6 (49.1-62.1) | 32.0 (23.5-40.4) |
| Myanmar | 13.1 (9.2-17.0) | 8.1 (4.9-11.2) | 51.5 (44.5-58.4) | 43.7 (37.2-50.2) | 27.9 (22.4-33.4) | 46.6 (39.4-53.9) | 44.7 (38.8-50.7) |
| Nepal | 17.1 (9.7-24.6) | 27.0 (19.2-34.8) | 37.3 (29.2-45.3) | 38.2 (31.7-44.6) | 57.1 (48.1-66.2) | 65.4 (56.6-74.2) | 59.7 (52.5-66.9) |
| Sri Lanka | 8.0 (0.0-70.8) | 17.4 (0.0-100) | 40.4 (0.0-100) | 47.9 (0.0-100) | 28.2 (0.0-100) | 62.1 (51.3-72.9) | 31.7 (21.9-41.5) |
| Thailand | NA | 16.7 (9.2-24.2) | NA | 57.0 (50.4-63.5) | 35.7 (26.1-45.3) | 39.2 (32.7-45.8) | 22.6 (17.8-27.4) |
| Timor-Leste | 63.4 (49.6-77.3) | 33.9 (29.0-38.7) | NA | 51.2 (45.3-57.1) | 31.4 (25.0-37.9) | NA | NA |
| **Western Pacific** |  |  |  |  |  |  |  |
| Brunei Darussalam | 12.1 (6.4-17.8) | 13.4 (6.6-20.2) | 48.1 (41.0-55.3) | 22.6 (16.3-28.8) | 11.2 (5.9-16.5) | 32.0 (20.0-44.0) | 16.2 (8.0-24.4) |
| Cambodia | 23.7 (4.6-42.8) | 25.8 (7.4-44.1) | 74.0 (60.4-87.7) | 34.2 (13.7-54.6) | 26.0 (9.5-42.4) | 15.6 (-2.6-33.7) | 15.4 (-0.1-30.9) |
| Cook Islands | 24.0 (15.5-32.5) | 40.0 (30.2-49.8) | 27.0 (18.1-35.9) | 39.0 (29.3-48.7) | 33.0 (23.3-42.7) | 37.8 (28.0-47.5) | 14.4 (7.3-21.6) |
| Fiji | 29.5 (23.1-36.0) | 27.8 (21.0-34.5) | 35.2 (28.5-41.9) | 43.8 (34.2-53.4) | 44.1 (36.8-51.4) | 57.1 (47.9-66.3) | 30.5 (24.4-36.6) |
| Guam | 27.1 (19.5-34.8) | 30.6 (23.1-38.2) | 28.3 (21.4-35.2) | 39.5 (31.1-47.9) | 37.0 (28.7-45.2) | 37.5 (30.4-44.7) | 19.4 (13.1-25.6) |
| Kiribati | 18.4 (14.1-22.6) | 12.9 (9.4-16.4) | 31.7 (27.7-35.7) | 47.4 (42.3-52.5) | 18.7 (13.7-23.6) | NA | NA |
| Laos | 15.9 (11.3-20.6) | 20.7 (16.1-25.2) | 42.8 (32.5-53.1) | 68.3 (64.1-72.6) | 33.7 (28.3-39.1) | 49.1 (44.5-53.7) | 35.0 (30.1-39.9) |
| Macao (China) | 11.1 (4.8-17.4) | 8.1 (3.0-13.2) | 28.4 (19.9-36.8) | 32.6 (25.4-39.9) | 17.5 (6.3-28.7) | 7.9 (3.8-12.1) | 12.2 (7.2-17.3) |
| Marshall Islands | 51.7 (45.6-57.7) | 41.6 (35.6-47.6) | NA | 60.6 (55.9-65.4) | 38.1 (32.3-43.9) | 35.0 (29.1-41.0) | 36.6 (30.9-42.3) |
| Micronesia | 43.5 (40.0-47.1) | 39.1 (35.9-42.4) | 32.4 (29.6-35.2) | 47.8 (44.8-50.9) | 34.7 (31.8-37.6) | 55.3 (51.9-58.7) | 28.5 (25.5-31.4) |
| Mongolia | 14.5 (9.8-19.1) | 18.6 (13.7-23.6) | NA | 51.8 (45.8-57.9) | 15.2 (10.0-20.4) | 26.8 (20.4-33.2) | 6.9 (3.6-10.1) |
| New Caledonia | 3.1 (0.8-5.5) | 13.0 (7.8-18.2) | 49.8 (42.1-57.5) | 23.0 (17.6-28.5) | 43.0 (38.2-47.9) | 39.1 (31.2-47.0) | 22.3 (16.3-28.4) |
| New Zealand | 7.5 (4.8-10.1) | 15.4 (9.2-21.7) | NA | 27.0 (21.0-32.9) | NA | NA | NA |
| Niue | 27.7 (-3.2-58.6) | 31.7 (11.3-52.1) | 41.4 (8.5-74.4) | 32.8 (13.0-52.6) | 50.5 (22.9-78.1) | 49.9 (26.0-73.8) | 32.5 (12.0-52.9) |
| Northern Mariana Islands | NA | 22.7 (17.4-28.0) | NA | 38.1 (30.6-45.7) | 33.6 (28.0-39.2) | 40.2 (33.4-46.9) | 16.5 (11.7-21.3) |
| Palau | NA | 19.1 (14.6-23.6) | 25.3 (21.3-29.3) | 27.9 (22.8-33.0) | 33.6 (28.3-39.0) | NA | NA |
| Papua New Guinea | 36.0 (27.3-44.7) | 36.2 (29.5-42.9) | 28.7 (22.2-35.3) | 53.4 (44.8-62.0) | 27.8 (22.4-33.2) | 53.6 (45.4-61.8) | 17.8 (14.6-20.9) |
| Philippines | 28.2 (24.5-31.9) | 21.2 (16.9-25.5) | 29.2 (24.5-33.8) | 48.0 (43.5-52.5) | 25.1 (20.7-29.5) | 49.0 (44.3-53.6) | 26.1 (22.3-30.0) |
| Samoa | 35.9 (24.5-47.2) | 17.9 (11.0-24.9) | NA | 61.1 (48.7-73.5) | 29.3 (20.9-37.8) | NA | NA |
| South Korea | NA | 9.5 (5.0-14.0) | NA | 27.5 (22.0-32.9) | 20.3 (15.5-25.1) | NA | NA |
| Tokelau | 19.2 (0.0-46.3) | 23.5 (8.2-38.7) | 38.3 (19.5-57.1) | 33.4 (6.6-60.2) | 54.2 (35.7-72.7) | 41.4 (20.2-62.6) | 30.2 (10.2-50.3) |
| Tonga | NA | 37.6 (32.6-42.7) | 32.7 (23.1-42.3) | 57.2 (46.8-67.5) | 33.5 (28.2-38.9) | 67.9 (60.1-75.8) | 33.6 (24.7-42.5) |
| Tuvalu | 15.6 (6.6-24.6) | 40.0 (26.5-53.5) | 39.1 (28.0-50.2) | 43.6 (32.6-54.5) | 19.7 (10.4-29.1) | NA | NA |
| Vanuatu | NA | 40.1 (30.5-49.8) | 22.5 (15.8-29.2) | 40.6 (32.6-48.7) | 45.2 (36.7-53.7) | 74.3 (67.0-81.5) | 29.7 (21.1-38.4) |
| Viet Nam | 12.1 (4.2-20.1) | 7.8 (1.1-14.5) | 76.9 (66.6-87.3) | 44.4 (34.3-54.5) | 32.8 (21.5-44.1) | 29.8 (18.8-40.7) | 17.5 (10.9-24.0) |

Abbreviation: NA, not available.

Data are presented as %(95%CI).

Current any tobacco use was defined as using either cigarettes or other tobacco products (e.g., chewing tobacco, snuff, dip, cigars, cigarillos, pipe, e-cigarettes) on at least 1 day during the past 30 day

**Table S4. Proportions of incorrect beliefs and attitudes toward tobacco smoking among adolescents aged 12–16 years who are non-smokers, by country/territory.**

| **Country/territory** | **Tobacco smoking is not harmful** | **Secondhand smoke exposure is not harmful** | **It is safe to smoke one or two years then quit** | **Once someone smoke, it is easy to quit** | **Tobacco smoking helps people feel more comfortable** | **Tobacco smoking makes more friends** | **Tobacco smoking is more attractive** |
| --- | --- | --- | --- | --- | --- | --- | --- |
| **Africa** |  |  |  |  |  |  |  |
| Algeria | NA | 8.0 (6.6-9.3) | NA | 35.1 (33.0-37.2) | 28.4 (26.3-30.6) | NA | NA |
| Angola | 26.9 (18.7-35.1) | 26.1 (18.1-34.1) | NA | 74.5 (61.1-87.9) | NA | NA | 39.9 (27.8-51.9) |
| Cameroon | NA | 34.8 (28.6-41.1) | NA | 54.9 (46.9-62.9) | 23.4 (19.0-27.8) | NA | NA |
| Chad | NA | 37.8 (35.2-40.5) | NA | NA | 29.4 (26.8-31.9) | 19.8 (17.6-22.0) | 22.7 (20.4-25.1) |
| Comoros | NA | 39.3 (33.1-45.6) | NA | 48.5 (44.2-52.8) | 24.0 (20.1-27.8) | NA | NA |
| Congo | NA | 36.2 (30.6-41.9) | NA | 66.3 (61.5-71.1) | 12.5 (10.2-14.7) | 24.4 (21.7-27.1) | 23.9 (21.3-26.5) |
| Gabon | NA | 28.5 (23.6-33.5) | NA | 30.4 (25.9-34.9) | 17.8 (15.4-20.2) | NA | NA |
| Gambia | 40.5 (34.9-46.1) | 33.4 (29.2-37.7) | 19.0 (15.8-22.2) | 61.6 (57.9-65.4) | 26.2 (23.8-28.6) | 22.0 (19.8-24.2) | 12.3 (10.6-14.0) |
| Ghana | NA | 28.2 (24.3-32.2) | NA | 50.2 (46.0-54.3) | 17.1 (14.3-19.9) | NA | NA |
| Kenya | NA | 16.2 (10.4-22.0) | NA | 52.2 (47.3-57.0) | 14.5 (12.1-16.9) | NA | NA |
| Madagascar | NA | 4.4 (2.4-6.4) | NA | 47.4 (39.9-54.9) | 5.0 (2.8-7.3) | NA | NA |
| Mauritania | 25.2 (19.0-31.4) | 24.4 (18.8-29.9) | 47.7 (42.2-53.2) | 67.2 (62.9-71.5) | 16.9 (14.3-19.6) | 25.3 (21.7-29.0) | 25.2 (22.4-28.0) |
| Mauritius | 12.2 (7.4-17.0) | 12.8 (8.7-16.9) | 13.7 (11.9-15.4) | 27.1 (23.1-31.1) | 13.5 (10.6-16.4) | 23.9 (19.1-28.8) | 8.4 (5.6-11.2) |
| Mozambique | NA | 28.7 (25.1-32.4) | NA | 45.7 (41.9-49.5) | 22.0 (20.0-23.9) | NA | NA |
| Sao Tome and Principe | 19.6 (18.4-20.8) | 22.1 (20.8-23.3) | 40.4 (38.9-41.9) | 58.7 (57.2-60.2) | 33.5 (32.1-35.0) | 19.2 (18.0-20.4) | 28.1 (26.8-29.5) |
| Senegal | NA | 48.7 (33.5-63.8) | NA | 43.9 (34.5-53.4) | 23.0 (17.8-28.2) | NA | NA |
| Seychelles | NA | 25.7 (21.7-29.8) | NA | 37.3 (33.4-41.2) | 23.5 (20.8-26.2) | NA | 11.1 (9.4-12.8) |
| Sierra Leone | 31.8 (26.4-37.1) | 23.7 (18.5-28.8) | NA | 58.8 (52.7-64.9) | 38.6 (32.6-44.7) | NA | NA |
| South Africa | 27.7 (23.6-31.9) | 29.8 (25.9-33.7) | 10.7 (9.5-11.9) | 48.5 (43.1-53.8) | 45.7 (42.1-49.3) | 57.2 (52.2-62.3) | 24.5 (21.8-27.2) |
| Togo | 28.8 (18.3-39.2) | 24.1 (16.2-32.0) | NA | 47.8 (38.1-57.6) | 7.4 (3.9-10.9) | 13.7 (10.3-17.0) | 10.6 (7.8-13.4) |
| Uganda | 33.6 (26.7-40.5) | 20.2 (16.0-24.3) | 8.3 (3.6-13.0) | 53.1 (47.7-58.5) | 16.0 (13.7-18.2) | 28.6 (23.3-33.9) | 15.3 (11.4-19.2) |
| United Republic of Tanzania | 11.6 (9.2-14.1) | 17.4 (13.1-21.8) | NA | 44.5 (41.1-47.8) | 23.5 (20.1-26.9) | NA | NA |
| Zambia | 35.2 (33.2-37.2) | 40.6 (38.5-42.6) | 15.0 (13.5-16.5) | 40.2 (38.1-42.2) | 30.7 (28.7-32.6) | 35.9 (33.9-37.9) | 23.7 (21.9-25.5) |
| Zimbabwe | 37.9 (31.3-44.5) | 37.5 (29.2-45.9) | NA | 53.9 (48.7-59.2) | 19.9 (17.2-22.7) | 21.8 (18.4-25.3) | 15.4 (11.4-19.4) |
| **Americas** |  |  |  |  |  |  |  |
| Antigua and Barbuda | 13.2 (10.8-15.5) | 13.3 (10.9-15.6) | 12.0 (10.2-13.7) | 28.3 (25.7-31.0) | 34.5 (31.7-37.4) | 27.0 (24.5-29.5) | 7.2 (5.8-8.6) |
| Argentina | 21.5 (14.2-28.8) | 15.3 (10.4-20.2) | NA | 34.9 (27.5-42.3) | 23.2 (17.9-28.6) | 14.1 (11.7-16.6) | 8.1 (3.8-12.4) |
| Bahamas | 12.9 (7.5-18.3) | 15.3 (10.7-20.0) | NA | 41.5 (26.2-56.8) | 26.8 (20.3-33.4) | NA | 15.9 (0.3-31.6) |
| Barbados | NA | 15.4 (13.0-17.9) | NA | 28.7 (25.5-31.9) | 41.7 (37.9-45.4) | NA | NA |
| Belize | NA | 13.8 (11.4-16.1) | NA | 29.8 (26.6-32.9) | 31.1 (27.3-34.9) | NA | NA |
| Bolivia | 25.4 (22.2-28.5) | 18.2 (15.9-20.5) | 17.6 (16.1-19.1) | 45.2 (41.1-49.2) | 29.0 (26.6-31.4) | 28.4 (22.4-34.5) | 13.2 (11.0-15.4) |
| Brazil | 7.4 (5.7-9.1) | 8.4 (6.6-10.2) | 7.7 (6.5-8.9) | 34.5 (31.4-37.5) | NA | NA | NA |
| Chile | NA | 9.0 (8.0-9.9) | 15.3 (14.3-16.2) | 25.1 (23.7-26.4) | 35.6 (33.7-37.4) | NA | NA |
| Costa Rica | 18.3 (15.6-21.0) | 14.3 (12.0-16.6) | 11.3 (10.1-12.4) | 27.9 (24.5-31.2) | 25.9 (23.5-28.3) | 18.1 (15.9-20.3) | 6.0 (4.9-7.1) |
| Cuba | 16.2 (13.1-19.3) | 10.5 (8.7-12.2) | 16.1 (14.6-17.6) | 42.2 (39.1-45.2) | 16.1 (14.3-17.8) | 11.5 (10.1-13.0) | 6.9 (5.5-8.2) |
| Dominican Republic | 16.2 (6.8-25.7) | 36.0 (28.9-43.1) | 63.8 (54.5-73.1) | 44.5 (37.6-51.3) | 30.4 (25.9-35.0) | 21.1 (17.7-24.5) | 8.9 (5.7-12.1) |
| Ecuador | NA | 31.3 (27.2-35.5) | NA | 41.8 (38.0-45.6) | 34.0 (30.9-37.1) | NA | NA |
| El Salvador | NA | 5.1 (4.1-6.1) | NA | 32.1 (29.2-35.0) | 30.1 (27.5-32.8) | NA | NA |
| Grenada | NA | 10.9 (8.8-13.0) | NA | 27.1 (24.2-30.1) | 31.5 (29.0-34.0) | NA | NA |
| Guatemala | 30.0 (26.8-33.2) | 10.7 (8.8-12.6) | 21.4 (20.0-22.7) | 35.0 (31.4-38.6) | 33.7 (31.4-36.1) | 28.8 (26.8-30.9) | 13.4 (11.3-15.5) |
| Guyana | 14.3 (10.0-18.5) | 25.6 (18.1-33.2) | 11.3 (9.2-13.4) | 47.9 (41.4-54.4) | 26.6 (23.0-30.2) | 39.0 (34.0-44.1) | 12.5 (9.5-15.5) |
| Honduras | NA | 26.8 (23.5-30.1) | NA | 44.5 (40.2-48.7) | 19.8 (16.9-22.6) | NA | NA |
| Jamaica | 10.9 (7.3-14.5) | 13.8 (11.8-15.8) | 13.4 (10.0-16.8) | 32.7 (30.0-35.4) | 42.4 (39.5-45.3) | 42.5 (37.3-47.8) | 11.1 (7.8-14.4) |
| Mexico | 5.4 (3.3-7.5) | 6.1 (4.3-7.9) | 19.7 (17.3-22.1) | 38.0 (34.6-41.4) | 29.1 (25.6-32.6) | 30.7 (27.8-33.5) | 16.8 (14.2-19.3) |
| Nicaragua | NA | 10.4 (9.2-11.6) | 10.2 (9.1-11.3) | 42.7 (40.1-45.4) | 19.9 (18.5-21.4) | 23.5 (21.3-25.6) | 9.5 (8.3-10.6) |
| Panama | 12.9 (10.8-15.0) | 8.9 (7.2-10.6) | 18.3 (16.8-19.8) | 47.3 (42.9-51.6) | 21.0 (18.7-23.4) | 15.5 (14.1-16.9) | 10.3 (8.6-12.0) |
| Paraguay | 37.2 (32.1-42.3) | 27.8 (24.1-31.5) | 15.6 (13.9-17.4) | 39.4 (36.0-42.8) | 28.3 (26.0-30.5) | 19.3 (16.9-21.6) | NA |
| Peru | NA | 9.1 (7.4-10.8) | NA | 33.2 (30.5-35.9) | 21.8 (19.5-24.1) | NA | NA |
| Saint Kitts and Nevis | 9.8 (6.5-13.1) | 15.4 (11.5-19.4) | 13.2 (9.8-16.6) | 28.9 (23.2-34.7) | 28.8 (24.9-32.7) | 38.6 (31.5-45.7) | 7.7 (4.7-10.7) |
| Saint Lucia | 16.3 (13.1-19.5) | 15.1 (11.8-18.3) | 12.4 (9.5-15.2) | 37.8 (34.7-40.8) | 33.4 (30.4-36.3) | 27.5 (24.4-30.6) | 9.8 (7.6-12.1) |
| Saint Vincent and the Grenadines | 12.6 (9.8-15.4) | 14.1 (11.7-16.5) | 16.4 (13.6-19.3) | 31.6 (28.2-34.9) | 40.0 (35.8-44.2) | 39.9 (36.9-42.8) | 6.9 (5.4-8.4) |
| Suriname | 8.9 (7.1-10.7) | 8.7 (6.6-10.8) | NA | 25.3 (22.6-28.0) | 45.0 (41.4-48.5) | 40.1 (37.0-43.1) | NA |
| Trinidad and Tobago | 13.1 (10.1-16.0) | 17.9 (15.1-20.8) | 13.8 (12.5-15.2) | 28.7 (24.4-33.0) | 29.0 (25.9-32.1) | 34.0 (30.8-37.2) | 8.1 (6.5-9.7) |
| Uruguay | NA | 9.0 (7.8-10.2) | 15.9 (14.5-17.3) | 32.7 (30.9-34.6) | 21.6 (19.7-23.5) | NA | NA |
| Venezuela | NA | 29.6 (26.2-33.1) | NA | 39.4 (35.7-43.2) | 23.3 (21.1-25.6) | NA | NA |
| **Eastern Mediterranean** |  |  |  |  |  |  |  |
| Afghanistan | 17.9 (9.0-26.8) | 21.2 (16.7-25.7) | 51.6 (39.7-63.6) | 36.4 (32.1-40.6) | 16.4 (13.4-19.3) | 31.8 (17.6-46.0) | 55.4 (44.4-66.3) |
| Bahrain | 18.9 (13.6-24.2) | 17.0 (14.1-19.8) | NA | 32.1 (27.6-36.6) | 20.4 (18.2-22.7) | 27.4 (24.7-30.1) | 18.0 (15.3-20.8) |
| Djibouti | NA | 50.1 (43.3-56.9) | NA | 58.8 (54.0-63.6) | 31.8 (26.7-36.9) | NA | NA |
| Egypt | 12.0 (8.6-15.5) | 16.0 (8.4-23.7) | 19.4 (11.2-27.7) | 57.1 (44.8-69.5) | 16.8 (9.5-24.1) | 33.6 (22.1-45.0) | 11.6 (7.0-16.2) |
| Gaza Strip | NA | 15.5 (9.5-21.6) | NA | 49.5 (43.1-56.0) | 15.0 (11.2-18.9) | NA | 10.4 (5.9-14.9) |
| Iraq | 16.3 (11.2-21.5) | 21.6 (18.1-25.0) | NA | 56.5 (51.8-61.2) | 18.7 (15.3-22.2) | 23.3 (18.0-28.5) | 11.2 (7.6-14.7) |
| Jordan | 12.5 (9.1-15.9) | 13.2 (9.6-16.8) | NA | 34.6 (30.2-39.1) | 20.6 (17.9-23.3) | 35.7 (33.4-38.1) | 16.5 (13.6-19.4) |
| Kuwait | 11.1 (8.4-13.9) | 12.4 (10.5-14.3) | NA | 31.0 (27.5-34.5) | 20.1 (17.9-22.3) | 23.2 (20.5-25.9) | 18.5 (16.1-21.0) |
| Lebanon | 4.7 (2.9-6.5) | 3.8 (2.1-5.6) | NA | 23.4 (19.7-27.0) | 15.7 (12.6-18.8) | 16.5 (14.4-18.7) | 12.9 (10.4-15.3) |
| Libyan Arab Jamahiriya | 6.9 (5.5-8.3) | 18.1 (15.2-21.0) | 39.3 (36.0-42.6) | NA | 11.5 (8.8-14.3) | NA | 18.3 (15.7-20.8) |
| Morocco | 10.4 (8.5-12.3) | 14.0 (11.8-16.2) | 76.6 (74.6-78.6) | 43.8 (40.8-46.7) | 16.9 (12.8-21.1) | NA | 18.8 (16.0-21.5) |
| Oman | 8.3 (6.0-10.7) | 12.2 (7.2-17.1) | 45.9 (41.9-49.8) | 45.0 (40.5-49.5) | 15.5 (12.1-19.0) | 22.9 (20.1-25.7) | 13.2 (9.9-16.5) |
| Pakistan | 20.5 (15.8-25.3) | 10.8 (7.9-13.8) | 32.2 (26.9-37.5) | 61.2 (56.2-66.1) | 63.1 (54.9-71.3) | 18.2 (14.4-21.9) | 39.1 (32.0-46.1) |
| Qatar | NA | 19.2 (15.9-22.6) | NA | 33.4 (29.2-37.6) | 18.2 (15.2-21.2) | NA | NA |
| Saudi Arabia | 6.6 (4.5-8.6) | 13.4 (10.6-16.2) | 41.9 (38.5-45.2) | NA | 12.7 (9.5-15.9) | NA | 23.0 (19.0-27.1) |
| Syrian Arab Republic | 9.6 (7.2-12.0) | 18.9 (12.4-25.4) | 54.1 (49.7-58.6) | NA | 12.0 (9.6-14.4) | NA | 16.1 (12.6-19.7) |
| Tunisia | 5.2 (3.7-6.7) | 11.7 (9.4-14.0) | 42.8 (39.0-46.5) | 43.1 (39.6-46.7) | 18.4 (15.9-20.9) | NA | 19.6 (16.4-22.7) |
| United Arab Emirates | NA | 12.6 (10.4-14.8) | 11.1 (9.9-12.4) | 28.0 (25.2-30.8) | 24.4 (21.6-27.2) | 23.1 (21.4-24.8) | NA |
| UNRWA GAZA | NA | 14.4 (10.8-18.1) | NA | 43.9 (38.7-49.1) | 16.3 (12.2-20.5) | 30.6 (24.2-37.0) | NA |
| UNRWA Jordan | NA | 16.5 (13.0-20.0) | NA | 41.0 (36.2-45.8) | 17.2 (14.3-20.0) | 33.3 (28.6-37.9) | NA |
| UNRWA Lebanon | NA | 12.4 (8.8-15.9) | NA | 37.6 (32.5-42.8) | 16.8 (13.3-20.4) | 28.6 (25.1-32.1) | NA |
| UNRWA West bank | NA | 11.3 (7.2-15.5) | NA | 34.7 (29.9-39.6) | 17.2 (13.9-20.5) | 30.2 (27.9-32.6) | NA |
| West BANK | NA | 15.2 (12.1-18.4) | NA | 43.1 (37.1-49.0) | 18.4 (14.5-22.4) | NA | 16.0 (12.1-19.8) |
| Yemen | NA | 17.9 (12.6-23.1) | NA | 46.2 (39.2-53.2) | 17.4 (13.1-21.7) | NA | 12.9 (9.5-16.3) |
| **Europe** |  |  |  |  |  |  |  |
| Albania | 14.2 (12.1-16.4) | 10.0 (8.3-11.7) | 47.2 (45.0-49.4) | 25.6 (23.3-27.8) | 23.0 (20.7-25.2) | 21.0 (19.2-22.9) | NA |
| Azerbaijan | 7.6 (4.5-10.6) | 7.6 (5.0-10.2) | 37.2 (32.5-41.9) | 59.3 (54.2-64.4) | 24.6 (21.7-27.5) | 18.1 (15.7-20.6) | 28.4 (24.1-32.7) |
| Belarus | 4.2 (3.0-5.4) | 7.6 (5.9-9.3) | NA | 14.2 (12.9-15.4) | 19.7 (18.2-21.1) | NA | 2.7 (1.9-3.5) |
| Bosnia and Herzegovina | 5.0 (4.0-6.0) | 6.7 (5.7-7.7) | NA | 16.7 (14.8-18.5) | 42.7 (40.0-45.4) | 15.5 (14.1-16.8) | 13.4 (12.2-14.6) |
| Bulgaria | NA | 11.2 (8.5-13.9) | NA | 33.9 (30.2-37.6) | 27.3 (25.3-29.4) | NA | NA |
| Croatia (Hrvatska) | 5.7 (4.2-7.2) | 7.3 (5.4-9.1) | 40.4 (37.8-43.1) | 16.1 (14.0-18.3) | 41.8 (37.9-45.6) | 26.1 (21.9-30.2) | 20.2 (17.0-23.3) |
| Cyprus | 10.3 (8.2-12.4) | 11.1 (9.0-13.3) | 16.9 (14.3-19.4) | NA | 24.0 (21.0-26.9) | 32.2 (29.0-35.4) | 23.4 (20.5-26.2) |
| Czech Republic | 6.1 (5.1-7.1) | 7.6 (6.4-8.9) | 17.0 (14.4-19.6) | 10.0 (8.4-11.6) | 37.9 (34.6-41.2) | 33.8 (30.0-37.5) | 10.6 (8.0-13.1) |
| Finland | 4.8 (3.9-5.6) | 20.3 (18.4-22.3) | 33.8 (32.1-35.6) | 17.6 (16.1-19.2) | NA | NA | NA |
| Georgia | 9.3 (6.7-11.9) | 7.9 (5.4-10.3) | NA | 78.4 (74.5-82.2) | 31.2 (25.5-36.8) | 15.0 (10.5-19.5) | 9.9 (7.8-12.0) |
| Greece | NA | 5.0 (3.8-6.1) | NA | 17.1 (15.7-18.6) | 23.4 (22.0-24.8) | NA | NA |
| Italy | 3.2 (1.9-4.5) | 7.3 (5.7-9.0) | NA | 8.6 (6.8-10.5) | 40.5 (37.1-43.8) | 21.9 (18.3-25.5) | 13.8 (10.9-16.8) |
| Kazakhstan | 8.2 (4.4-12.0) | 8.1 (5.4-10.7) | 44.3 (37.4-51.1) | 23.7 (19.0-28.4) | 11.6 (8.7-14.5) | 10.3 (7.1-13.5) | 12.1 (7.1-17.0) |
| Kosovo | 7.5 (6.1-8.9) | 10.3 (9.4-11.2) | NA | 29.2 (26.6-31.8) | 20.3 (18.2-22.3) | 14.3 (12.3-16.2) | 14.9 (13.7-16.1) |
| Kyrgyzstan | NA | 11.2 (9.6-12.9) | NA | 34.0 (31.1-37.0) | 27.4 (24.6-30.3) | NA | NA |
| Latvia | 5.6 (4.2-7.0) | 7.5 (5.9-9.1) | 38.3 (36.3-40.3) | 15.3 (13.2-17.4) | 23.4 (21.2-25.7) | 22.6 (20.3-24.9) | 6.1 (4.0-8.2) |
| Lithuania | 10.7 (8.6-12.8) | 14.2 (12.4-15.9) | NA | 47.9 (45.1-50.7) | 27.8 (25.2-30.5) | 32.2 (29.6-34.9) | 6.8 (5.5-8.0) |
| Macedonia | 11.4 (9.2-13.5) | 8.8 (7.5-10.2) | NA | 27.4 (24.6-30.2) | 21.7 (20.0-23.4) | NA | 13.7 (11.7-15.6) |
| Malta | NA | 7.8 (6.3-9.3) | NA | 15.7 (13.6-17.8) | 39.7 (36.9-42.5) | NA | NA |
| Montenegro | 8.0 (5.9-10.0) | 9.9 (8.6-11.1) | NA | 27.0 (24.5-29.6) | 36.3 (34.1-38.6) | 15.5 (13.4-17.5) | 12.7 (11.0-14.5) |
| Poland | 12.7 (10.3-15.0) | 15.9 (14.0-17.8) | 13.3 (12.0-14.7) | 21.2 (18.6-23.8) | 38.2 (36.4-40.1) | 22.5 (19.8-25.2) | 5.8 (4.3-7.3) |
| Portugal | NA | 10.5 (9.0-12.1) | NA | 21.6 (19.2-23.9) | 29.1 (27.8-30.4) | NA | 9.5 (8.6-10.4) |
| Republic of Moldova | NA | 13.3 (11.8-14.7) | NA | 27.6 (24.8-30.4) | 27.7 (25.6-29.8) | NA | NA |
| Romania | 10.4 (8.3-12.5) | 15.9 (13.7-18.1) | NA | 27.1 (24.9-29.4) | 23.9 (21.9-25.9) | 16.5 (14.9-18.1) | 9.3 (8.2-10.5) |
| Russian Federation | NA | 10.8 (9.4-12.2) | NA | 13.7 (12.0-15.4) | 22.3 (19.1-25.4) | NA | NA |
| San Marino | 5.5 (2.0-9.0) | 12.2 (9.2-15.1) | 20.5 (16.1-24.9) | 6.3 (4.3-8.4) | 45.8 (41.8-49.7) | 18.0 (14.3-21.7) | 13.6 (10.6-16.5) |
| Serbia | 7.1 (6.3-8.0) | 8.1 (7.2-9.0) | NA | 22.6 (21.1-24.0) | 51.9 (50.2-53.6) | NA | NA |
| Slovakia | 6.0 (4.6-7.5) | 8.0 (6.9-9.2) | 14.9 (13.6-16.2) | 18.8 (17.2-20.4) | 44.8 (41.9-47.7) | 17.9 (16.2-19.5) | 8.6 (7.1-10.1) |
| Slovenia | 3.7 (2.0-5.4) | 5.8 (4.2-7.3) | 21.6 (19.0-24.2) | 20.8 (18.1-23.6) | 50.8 (48.2-53.4) | 27.8 (24.9-30.7) | 7.6 (5.4-9.8) |
| Tajikistan | 8.6 (6.5-10.6) | 17.1 (10.6-23.5) | NA | 25.1 (21.1-29.1) | 23.8 (21.0-26.5) | NA | 51.6 (44.1-59.0) |
| Turkey | 6.8 (6.1-7.5) | 5.4 (5.0-5.8) | 65.8 (55.6-76.1) | 36.2 (35.2-37.3) | 24.3 (23.4-25.1) | 30.1 (29.2-31.0) | 21.6 (15.5-27.7) |
| Turkmenistan | NA | 2.9 (1.9-3.9) | NA | 6.7 (4.3-9.2) | 7.4 (3.3-11.5) | NA | NA |
| Ukraine | 11.1 (8.0-14.1) | 14.4 (10.7-18.2) | 46.7 (41.9-51.5) | 26.5 (23.4-29.6) | 24.3 (19.4-29.3) | 31.3 (27.7-34.9) | 7.2 (5.3-9.1) |
| **South-East Asia** |  |  |  |  |  |  |  |
| Bangladesh | NA | 9.3 (5.1-13.5) | NA | 45.2 (39.7-50.7) | 33.0 (22.9-43.1) | NA | NA |
| Bhutan | NA | 9.4 (7.3-11.4) | NA | 29.7 (26.4-33.0) | 14.5 (12.7-16.3) | NA | NA |
| Indonesia | 5.7 (4.6-6.8) | 4.4 (3.4-5.4) | 22.3 (20.4-24.3) | 17.9 (15.8-20.1) | 10.1 (8.9-11.2) | 15.6 (12.4-18.8) | 3.7 (2.7-4.6) |
| Maldives | 4.3 (3.0-5.7) | 12.7 (10.8-14.5) | NA | 28.3 (26.0-30.6) | 16.7 (15.0-18.3) | 35.0 (31.0-39.1) | 13.2 (11.5-14.9) |
| Myanmar | 5.9 (4.6-7.2) | 4.3 (3.1-5.6) | 48.9 (45.0-52.9) | 27.1 (23.9-30.4) | 25.6 (20.0-31.1) | 39.1 (34.6-43.5) | 35.9 (30.4-41.5) |
| Nepal | 9.7 (6.9-12.5) | 17.1 (13.8-20.4) | 32.4 (27.4-37.4) | 28.9 (24.8-32.9) | 53.2 (48.7-57.8) | 54.8 (49.6-60.0) | 52.0 (48.1-56.0) |
| Sri Lanka | 6.6 (4.6-8.6) | 6.4 (4.9-7.9) | 12.4 (10.4-14.5) | 63.5 (58.2-68.8) | 24.2 (18.5-30.0) | 53.6 (51.4-55.9) | 12.5 (11.0-14.0) |
| Thailand | NA | 7.7 (6.0-9.3) | NA | 46.0 (41.3-50.7) | 26.5 (24.8-28.2) | 20.9 (17.4-24.4) | 9.8 (7.3-12.3) |
| Timor-Leste | 69.7 (61.4-78.0) | 34.1 (30.2-38.0) | NA | 53.8 (49.8-57.9) | 41.9 (39.2-44.5) | NA | NA |
| **Western Pacific** |  |  |  |  |  |  |  |
| Brunei Darussalam | 4.5 (2.9-6.0) | 5.2 (3.6-6.7) | 29.3 (26.6-31.9) | 14.5 (12.0-16.9) | 10.5 (7.4-13.6) | 17.6 (15.1-20.0) | 4.1 (3.1-5.1) |
| Cambodia | 6.6 (4.9-8.3) | 5.6 (4.5-6.7) | 89.3 (87.1-91.6) | 24.0 (21.4-26.7) | 10.6 (8.6-12.7) | 9.1 (6.8-11.4) | 11.6 (9.0-14.3) |
| Cook Islands | 19.5 (15.4-23.5) | 21.6 (17.4-25.9) | 20.5 (16.4-24.7) | 34.8 (29.9-39.7) | 29.4 (24.7-34.2 | 23.9 (19.5-28.3) | 8.9 (5.9-11.8) |
| Fiji | 20.6 (15.4-25.9) | 17.9 (14.4-21.5) | 15.8 (12.4-19.1) | 39.2 (33.9-44.4) | 25.2 (21.7-28.8) | 38.3 (33.0-43.5) | 17.7 (13.5-21.9) |
| Guam | 11.5 (8.9-14.1) | 16.9 (14.6-19.2) | 10.8 (7.2-14.3) | 27.3 (24.0-30.6) | 25.4 (23.2-27.7) | 29.7 (25.3-34.0) | 7.9 (5.4-10.4) |
| Kiribati | 14.3 (11.3-17.2) | 13.1 (10.3-15.9) | 21.9 (19.2-24.5) | 42.4 (39.4-45.5) | 24.1 (21.5-26.7) | NA | NA |
| Laos | 11.5 (9.4-13.6) | 13.7 (10.5-16.8) | 29.1 (26.3-32.0) | 68.6 (65.0-72.2) | 42.7 (40.0-45.3) | 33.9 (30.2-37.5) | 25.5 (22.5-28.5) |
| Macao (China) | 3.1 (2.2-3.9) | 3.9 (2.8-4.9) | 11.6 (9.4-13.9) | 13.5 (11.1-15.8) | 7.0 (3.3-10.8) | 6.1 (4.6-7.6) | 6.0 (4.9-7.1) |
| Marshall Islands | 49.5 (45.7-53.2) | 40.1 (36.4-43.9) | NA | 53.3 (49.8-56.7) | 31.0 (28.4-33.7) | 35.4 (32.5-38.2) | 28.5 (26.0-31.1) |
| Micronesia | 37.8 (35.0-40.7) | 35.9 (33.3-38.6) | 24.3 (22.6-26.1) | 46.7 (44.2-49.1) | 31.6 (29.7-33.5) | 49.6 (47.6-51.7) | 26.6 (24.7-28.6) |
| Mongolia | 7.3 (5.9-8.8) | 10.8 (9.0-12.6) | NA | 60.6 (57.2-64.1) | 6.0 (4.7-7.3) | 18.0 (15.8-20.2) | 2.9 (2.3-3.6) |
| New Caledonia | 3.7 (1.3-6.2) | 7.6 (4.8-10.4) | 62.6 (58.4-66.8) | 17.3 (13.6-21.0) | 51.0 (41.5-60.5) | 31.1 (24.3-37.9) | 14.8 (9.4-20.3) |
| New Zealand | 6.8 (3.5-10.2) | 7.0 (5.0-9.0) | NA | 13.4 (9.6-17.2) | NA | NA | NA |
| Niue | 24.3 (11.4-37.2) | 21.6 (11.0-32.3) | 11.2 (3.9-18.4) | 22.5 (14.8-30.1) | 38.3 (23.1-53.5) | 28.9 (23.9-33.9) | 9.9 (2.0-17.8) |
| Northern Mariana Islands | NA | 12.0 (10.3-13.7) | NA | 26.0 (23.0-29.1) | 27.4 (22.2-32.5) | 27.6 (21.0-34.2) | 7.1 (5.9-8.3) |
| Palau | NA | 12.2 (9.4-15.0) | 15.5 (12.7-18.3) | 26.6 (22.6-30.6) | 22.5 (18.8-26.2) | NA | NA |
| Papua New Guinea | 28.0 (20.8-35.2) | 29.9 (23.2-36.6) | 10.8 (8.1-13.5) | 47.4 (42.8-52.0) | 23.4 (20.3-26.4) | 42.6 (37.6-47.6) | 15.5 (12.9-18.0) |
| Philippines | 17.4 (15.4-19.4) | 14.4 (12.8-16.0) | 15.3 (14.2-16.5) | 38.8 (36.6-41.1) | 15.1 (13.8-16.3) | 30.8 (28.8-32.7) | 12.6 (11.1-14.2) |
| Samoa | 24.9 (19.5-30.2) | 9.0 (6.5-11.5) | NA | 71.7 (64.9-78.4) | 34.6 (31.6-37.7) | NA | NA |
| South Korea | NA | 3.0 (2.4-3.5) | NA | 26.9 (24.7-29.2) | 8.7 (7.5-9.8) | NA | NA |
| Tokelau | 26.0 (15.1-37.0) | 19.7 (10.5-29.0) | 21.8 (10.1-33.5) | 35.9 (22.5-49.3) | 32.3 (14.7-49.9) | 44.2 (24.1-64.2) | 26.4 (10.7-42.2) |
| Tonga | NA | 27.4 (23.6-31.2) | 22.2 (17.6-26.8) | 56.2 (52.8-59.7) | 37.8 (33.2-42.4) | 57.6 (52.9-62.2) | 24.3 (20.8-27.7) |
| Tuvalu | 12.6 (8.7-16.6) | 29.7 (22.8-36.6) | 43.6 (38.1-49.2) | 44.2 (37.2-51.2) | 25.1 (19.1-31.2) | NA | NA |
| Vanuatu | NA | 47.1 (40.0-54.2) | 10.7 (7.2-14.1) | 49.3 (44.3-54.4) | 35.4 (30.4-40.3) | 66.2 (62.2-70.1) | 25.7 (20.2-31.1) |
| Viet Nam | 6.4 (5.1-7.7) | 4.8 (3.7-5.9) | 89.9 (87.3-92.4) | 23.4 (21.1-25.7) | 26.6 (23.8-29.4) | 19.4 (16.9-21.8) | 6.9 (5.7-8.2) |

Abbreviation: NA, not available.

Data are presented as %(95%CI).

Current any tobacco use was defined as using either cigarettes or other tobacco products (e.g., chewing tobacco, snuff, dip, cigars, cigarillos, pipe, e-cigarettes) on at least 1 day during the past 30 days.

**TableS5 Proportions of incorrect beliefs and positive attitudes toward tobacco smoking among adolescents aged 12–16 years who are current smokers, stratified by age, sex, WHO region, World Bank income category, secondhand smoke exposure status, FCTC ratification, tobacco use monitoring, tobacco warning policies, and enforcement of tobacco advertising bans.**

|  | **Tobacco smoking is not harmful** | **Secondhand smoke exposure is not harmful** | **It is safe to smoke one or two years then quit** | **Once someone smoke, it is easy to quit** | **Tobacco smoking helps people feel more comfortable** | **Tobacco smoking makes more friends** | **Tobacco smoking is more attractive** |
| --- | --- | --- | --- | --- | --- | --- | --- |
| **Total** | 17.2 (13.7-20.7) | 20.4 (19.1-21.8) | 53.1 (48.9-57.2) | 39.1 (36.2-41.9) | 36.1 (33.9-38.3) | 30.7 (26.9-34.4) | 20.4 (17.4-23.4) |
| **Sex** |  |  |  |  |  |  |  |
| Boys | 19.3 (15.1-23.6) | 20.5 (18.6-22.4) | 54.7 (50.8-58.5) | 38.7 (34.5-42.9) | 34.3 (31.4-37.2) | 32.3 (27.4-37.1) | 19.5 (16.2-22.7) |
| Girls | 14.1 (10.1-18.1) | 20.4 (18.7-22.1) | 49.8 (42.5-57.1) | 39.5 (37.3-41.6) | 38.2 (35.9-40.5) | 28.1 (22.5-33.7) | 22.2 (19.0-25.3) |
| *P* value | 0.03 | 0.94 | 0.14 | 0.69 | 0.01 | 0.26 | 0.02 |
| **Age group** |  |  |  |  |  |  |  |
| 12-14 years | 18.3 (13.2-23.4) | 21.3 (19.7-22.8) | 49.4 (45.6-53.2) | 41.0 (38.0-44.1) | 37.1 (34.5-39.7) | 34.9 (29.2-40.6) | 24.0 (21.2-26.8) |
| 15-16 years | 15.6 (12.6-18.6) | 18.9 (16.9-21.0) | 58.6 (52.9-64.2) | 35.5 (31.6-39.3) | 34.2 (31.5-36.9) | 24.2 (20.4-28.1) | 15.2 (10.9-19.5) |
| *P* value | 0.33 | 0.04 | 0.001 | 0.005 | 0.06 | 0.003 | <0.001 |
| **WHO region** |  |  |  |  |  |  |  |
| Africa | 35.4 (31.7-39.1) | 29.9 (26.9-32.9) | 20.0 (16.8-23.1) | 50.1 (47.5-52.8) | 34.0 (31.1-36.9) | 51.2 (47.9-54.5) | 29.0 (26.0-32.0) |
| Americas | 15.8 (9.6-22.0) | 19.8 (16.0-23.6) | 54.3 (45.1-63.4) | 36.1 (29.3-43.0) | 36.7 (30.7-42.7) | 23.8 (18.3-29.3) | 15.2 (10.2-20.2) |
| Eastern Mediterranean | 22.1 (14.9-29.3) | 22.1 (19.5-24.6) | 46.9 (39.4-54.4) | 48.3 (40.2-56.3) | 35.9 (31.1-40.6) | 37.3 (28.9-45.7) | 30.5 (25.6-35.5) |
| Europe | 10.3 (8.0-12.7) | 20.4 (18.1-22.7) | 63.9 (61.6-66.2) | 27.5 (24.7-30.2) | 42.7 (39.6-45.8) | 24.4 (21.4-27.4) | 15.7 (14.1-17.3) |
| South-East Asia | 15.6 (13.1-18.0) | 15.2 (12.6-17.8) | 47.5 (44.5-50.5) | 38.6 (35.6-41.5) | 33.0 (29.4-36.7) | 44.1 (39.5-48.6) | 28.8 (25.5-32.2) |
| Western Pacific | 13.0 (10.6-15.3) | 15.2 (13.0-17.5) | 55.1 (50.8-59.5) | 34.8 (31.9-37.7) | 28.2 (25.4-31.0) | 37.8 (34.2-41.4) | 21.5 (19.1-24.0) |
| *P* value | 0.004 | <0.001 | <0.001 | <0.001 | 0.07 | <0.001 | <0.001 |
| **World Bank income category** |  |  |  |  |  |  |  |
| Low income | 24.6 (19.0-30.2) | 21.4 (18.2-24.6) | 33.5 (27.7-39.2) | 42.6 (39.0-46.2) | 37.5 (32.7-42.3) | 54.6 (48.6-60.6) | 47.3 (42.3-52.3) |
| Lower-Middle income | 20.2 (14.8-25.5) | 19.1 (17.0-21.3) | 47.4 (42.9-51.9) | 42.7 (37.7-47.6) | 32.1 (28.5-35.7) | 37.8 (32.9-42.6) | 25.3 (21.7-28.8) |
| Upper-Middle income | 16.9 (11.2-22.5) | 21.1 (18.3-24.0) | 58.1 (53.2-63.0) | 40.0 (34.8-45.2) | 37.0 (32.7-41.2) | 27.1 (22.2-32.1) | 16.9 (13.3-20.5) |
| High income | 9.3 (7.2-11.4) | 20.6 (18.2-23.0) | 42.0 (38.3-45.8) | 24.8 (22.1-27.6) | 41.5 (38.2-44.7) | 21.6 (18.1-25.1) | 18.3 (15.5-21.1) |
| *P* value | 0.04 | 0.54 | <0.001 | <0.001 | 0.03 | <0.001 | <0.001 |
| **Secondhand smoke exposure status**^∫^ |  |  |  |  |  |  |  |
| Yes | 17.0 (13.7-20.2) | 18.9 (17.4-20.3) | 50.0 (47.7-52.3) | 35.6 (32.8-38.3) | 36.8 (34.2-39.4) | 31.9 (28.0-35.7) | 21.9 (19.0-24.8) |
| No | 18.1 (9.6-26.5) | 27.8 (24.5-31.1) | 60.6 (47.9-73.4) | 55.9 (50.1-61.7) | 32.6 (29.0-36.2) | 27.2 (21.4-33.1) | 16.1 (10.9-21.4) |
| *P* value | 0.79 | <0.001 | 0.12 | <0.001 | 0.06 | 0.14 | 0.05 |
| **FCTC ratification** |  |  |  |  |  |  |  |
| Yes | 18.9 (15.9-21.9) | 21.2 (19.6-22.8) | 49.5 (47.0-52.0) | 40.3 (37.0-43.7) | 37.4 (34.9-39.9) | 32.9 (29.3-36.5) | 23.9 (21.9-25.9) |
| No | 14.6 (7.8-21.4) | 15.9 (13.2-18.6) | 58.9 (51.3-66.6) | 31.8 (28.4-35.1) | 27.7 (23.7-31.7) | 27.2 (20.3-34.2) | 12.6 (8.2-16.9) |
| *P* value | 0.30 | 0.003 | 0.02 | <0.001 | <0.001 | 0.17 | <0.001 |
| **Monitoring tobacco use** |  |  |  |  |  |  |  |
| Yes | 15.7 (10.4-20.9) | 15.9 (14.1-17.7) | 38.7 (33.6-43.7) | 36.8 (33.0-40.7) | 36.9 (33.6-40.2) | 30.2 (26.0-34.4) | 20.2 (15.7-24.7) |
| No | 17.8 (13.3-22.3) | 23.1 (21.0-25.2) | 56.3 (52.2-60.5) | 40.6 (36.5-44.6) | 35.6 (32.6-38.5) | 30.8 (26.1-35.5) | 20.5 (16.9-24.0) |
| *P* value | 0.56 | <0.001 | <0.001 | 0.20 | 0.57 | 0.86 | 0.93 |
| **Warning about the dangers of tobacco** |  |  |  |  |  |  |  |
| Yes | 17.8 (13.3-22.3) | 22.4 (21.1-23.8) | 56.8 (52.9-60.7) | 39.8 (38.2-41.3) | 34.3 (32.4-36.1) | 33.5 (27.4-39.6) | 19.9 (16.1-23.7) |
| No | 16.0 (10.4-21.6) | 18.2 (15.8-20.7) | 33.3 (27.8-38.7) | 38.4 (33.4-43.3) | 38.1 (33.8-42.3) | 26.2 (21.6-30.8) | 21.8 (17.7-25.8) |
| *P* value | 0.63 | 0.004 | <0.001 | 0.62 | 0.11 | 0.06 | 0.51 |
| **Enforcing tobacco advertising bans** |  |  |  |  |  |  |  |
| Yes | 17.2 (13.6-20.7) | 20.8 (18.5-23.2) | 53.2 (49.1-57.4) | 50.8 (47.3-54.3) | 28.8 (25.1-32.6) | 30.6 (26.8-34.3) | 20.3 (17.3-23.4) |
| No | 20.4 (17.6-23.2) | 20.4 (18.9-21.9) | 35.4 (30.8-39.9) | 38.0 (35.0-41.0) | 36.6 (34.3-39.0) | 39.1 (36.4-41.7) | 28.3 (24.0-32.5) |
| *P* value | 0.18 | 0.77 | <0.001 | <0.001 | 0.001 | <0.001 | 0.002 |

Data are presented as %(95%CI). WHO: World Health Organization;

^∫^ Current secondhand smoke exposure was defined as exposure to secondhand smoke in any place (at home or in public places) on at least 1 day during the past 7 days.

**TableS6 Proportions of incorrect beliefs and positive attitudes toward tobacco smoking among adolescents aged 12–16 years who are non-smokers, stratified by age, sex, WHO region, World Bank income category, secondhand smoke exposure status, FCTC ratification, tobacco use monitoring, tobacco warning policies, and enforcement of tobacco advertising bans.**

|  | **Tobacco smoking is not harmful** | **Secondhand smoke exposure is not harmful** | **It is safe to smoke one or two years then quit** | **Once someone smoke, it is easy to quit** | **Tobacco smoking helps people feel more comfortable** | **Tobacco smoking makes more friends** | **Tobacco smoking is more attractive** |
| --- | --- | --- | --- | --- | --- | --- | --- |
| **Total** | 12.7 (10.2-15.2) | 15.0 (14.1-16.0) | 41.4 (34.6-48.2) | 40.0 (38.9-41.2) | 24.6 (23.5-25.7) | 25.8 (24.0-27.6) | 15.0 (13.6-16.3) |
| **Sex** |  |  |  |  |  |  |  |
| Boys | 15.8 (12.7-18.9) | 16.9 (15.7-18.2) | 40.6 (34.8-46.4) | 44.1 (42.7-45.5) | 26.1 (24.6-27.7) | 25.6 (23.5-27.7) | 17.1 (15.4-18.8) |
| Girls | 10.0 (7.9-12.2) | 13.2 (12.2-14.2) | 42.0 (34.2-49.9) | 36.1 (34.5-37.6) | 23.0 (21.7-24.3) | 26.0 (23.6-28.4) | 13.2 (11.7-14.7) |
| *P* value | <0.001 | <0.001 | 0.44 | <0.001 | 0.001 | 0.78 | <0.001 |
| **Age group** |  |  |  |  |  |  |  |
| 12-14 years | 12.7 (9.6-15.7) | 14.6 (13.6-15.7) | 44.5 (36.4-52.6) | 39.4 (38.1-40.8) | 24.0 (22.6-25.4) | 23.8 (21.4-26.2) | 13.5 (12.0-14.9) |
| 15-16 years | 12.8 (11.0-14.5) | 15.9 (14.6-17.2) | 33.7 (30.1-37.2) | 41.2 (39.8-42.6) | 25.6 (24.4-26.8) | 31.1 (28.7-33.4) | 18.9 (17.1-20.6) |
| *P* value | 0.94 | 0.05 | 0.002 | 0.04 | 0.05 | <0.001 | <0.001 |
| **WHO region** |  |  |  |  |  |  |  |
| Africa | 21.5 (19.2-23.7) | 23.5 (21.0-26.1) | 11.3 (9.0-13.6) | 48.4 (46.5-50.3) | 20.1 (18.9-21.3) | 34.8 (31.8-37.7) | 18.9 (17.1-20.7) |
| Americas | 15.7 (10.3-21.1) | 17.9 (16.0-19.8) | 50.6 (39.8-61.4) | 37.9 (36.0-39.8) | 26.1 (24.6-27.6) | 22.0 (20.1-23.9) | 10.1 (8.3-12.0) |
| Eastern Mediterranean | 11.4 (10.1-12.7) | 16.2 (13.7-18.6) | 37.6 (33.5-41.7) | 50.8 (46.3-55.3) | 18.7 (16.4-21.1) | 29.7 (23.0-36.3) | 17.1 (15.3-18.8) |
| Europe | 6.8 (6.0-7.6) | 9.9 (9.1-10.7) | 35.6 (33.8-37.4) | 22.7 (21.0-24.4) | 31.3 (29.8-32.8) | 22.3 (20.7-23.9) | 14.1 (12.7-15.4) |
| South-East Asia | 7.7 (6.6-8.7) | 9.1 (7.6-10.5) | 26.8 (25.0-28.5) | 37.9 (35.7-40.1) | 29.8 (26.6-33.0) | 31.9 (28.9-34.8) | 22.3 (19.4-25.1) |
| Western Pacific | 7.7 (6.9-8.5) | 5.8 (5.4-6.3) | 79.5 (77.6-81.5) | 27.6 (26.3-28.8) | 16.8 (15.6-18.0) | 18.4 (17.0-19.8) | 10.6 (9.5-11.6) |
| *P* value | <0.001 | <0.001 | <0.001 | <0.001 | <0.001 | <0.001 | <0.001 |
| **World Bank income category** |  |  |  |  |  |  |  |
| Low income | 15.0 (13.4-16.6) | 13.9 (12.3-15.6) | 28.5 (25.0-32.0) | 42.4 (40.4-44.3) | 27.3 (24.1-30.4) | 44.5 (40.9-48.1) | 40.9 (38.2-43.7) |
| Lower-Middle income | 10.2 (9.2-11.1) | 15.7 (13.7-17.8) | 36.2 (33.5-38.8) | 43.5 (41.2-45.9) | 20.1 (18.6-21.7) | 26.3 (22.3-30.3) | 13.7 (12.5-15.0) |
| Upper-Middle income | 15.1 (10.3-19.9) | 16.5 (15.1-17.9) | 50.1 (39.8-60.5) | 39.3 (37.8-40.9) | 25.5 (24.3-26.6) | 22.6 (20.9-24.2) | 10.9 (9.4-12.4) |
| High income | 5.9 (5.2-6.7) | 10.1 (9.2-10.9) | 30.5 (28.9-32.1) | 17.2 (15.9-18.5) | 30.5 (28.7-32.3) | 22.8 (20.6-25.0) | 14.7 (13.1-16.4) |
| *P* value | 0.003 | 0.002 | 0.001 | <0.001 | <0.001 | <0.001 | <0.001 |
| **Secondhand smoke exposure status**^∫^ |  |  |  |  |  |  |  |
| Yes | 10.4 (9.1-11.6) | 12.0 (10.9-13.1) | 40.0 (33.3-46.7) | 35.8 (34.4-37.2) | 25.9 (24.4-27.4) | 28.1 (25.9-30.3) | 15.4 (13.7-17.0) |
| No | 15.7 (11.5-20.0) | 19.4 (18.2-20.7) | 43.1 (35.8-50.3) | 46.2 (44.7-47.7) | 22.6 (21.6-23.7) | 22.7 (18.8-26.6) | 14.5 (12.0-17.0) |
| *P* value | <0.001 | <0.001 | 0.12 | <0.001 | <0.001 | 0.04 | 0.61 |
| **FCTC ratification** |  |  |  |  |  |  |  |
| Yes | 12.1 (11.3-13.0) | 15.0 (14.0-15.9) | 32.7 (30.7-34.7) | 41.2 (40.0-42.4) | 25.4 (24.2-26.6) | 28.9 (26.9-30.8) | 18.2 (17.0-19.3) |
| No | 13.9 (6.8-21.0) | 15.8 (12.2-19.5) | 54.8 (45.3-64.4) | 29.1 (25.4-32.8) | 17.1 (14.7-19.5) | 20.1 (17.4-22.7) | 8.3 (6.0-10.6) |
| *P* value | 0.62 | 0.66 | <0.001 | <0.001 | <0.001 | <0.001 | <0.001 |
| **Monitoring tobacco use** |  |  |  |  |  |  |  |
| Yes | 8.2 (7.1-9.3) | 9.2 (7.8-10.7) | 22.7 (19.1-26.3) | 33.7 (31.5-35.9) | 21.6 (20.0-23.3) | 25.2 (21.2-29.3) | 11.0 (9.4-12.6) |
| No | 14.2 (11.1-17.4) | 17.5 (16.4-18.7) | 46.8 (39.9-53.7) | 42.9 (41.7-44.1) | 25.8 (24.5-27.2) | 26.0 (24.0-28.0) | 16.2 (14.2-18.1) |
| *P* value | <0.001 | <0.001 | <0.001 | <0.001 | <0.001 | 0.73 | <0.001 |
| **Warning about the dangers of tobacco** |  |  |  |  |  |  |  |
| Yes | 10.7 (9.1-12.3) | 12.6 (11.2-14.0) | 24.3 (21.0-27.6) | 40.1 (38.0-42.1) | 24.5 (23.0-26.1) | 25.2 (22.3-28.1) | 11.5 (10.1-12.8) |
| No | 13.6 (10.1-17.1) | 16.6 (15.4-17.8) | 47.4 (40.3-54.5) | 40.0 (38.8-41.2) | 24.6 (23.1-26.0) | 26.2 (23.9-28.5) | 16.7 (14.4-19.0) |
| *P* value | 0.12 | <0.001 | <0.001 | 0.95 | 0.99 | 0.60 | <0.001 |
| **Enforcing tobacco advertising bans** |  |  |  |  |  |  |  |
| Yes | 13.2 (10.6-15.8) | 17.0 (15.3-18.8) | 30.6 (28.8-32.5) | 48.1 (46.0-50.2) | 14.6 (13.5-15.6) | 21.5 (20.1-22.9) | 13.9 (12.2-15.6) |
| No | 12.7 (10.1-15.3) | 14.8 (13.7-15.8) | 41.6 (34.7-48.4) | 38.9 (37.6-40.1) | 25.9 (24.7-27.2) | 25.9 (24.1-27.7) | 15.0 (13.6-16.4) |
| *P* value | 0.80 | 0.02 | 0.003 | <0.001 | <0.001 | <0.001 | 0.37 |

Data are presented as %(95%CI). WHO: World Health Organization;

^∫^ Current secondhand smoke exposure was defined as exposure to secondhand smoke in any place (at home or in public places) on at least 1 day during the past 7 days.

**Table S7. Proportions of incorrect beliefs and positive attitudes toward tobacco smoking among adolescents aged 12–16 years who are exposed to secondhand smoke, by country/territory.**

| **Country/territory** | **Tobacco smoking is not harmful** | **Secondhand smoke exposure is not harmful** | **It is safe to smoke one or two years then quit** | **Once someone smoke, it is easy to quit** | **Tobacco smoking helps people feel more comfortable** | **Tobacco smoking makes more friends** | **Tobacco smoking is more attractive** |
| --- | --- | --- | --- | --- | --- | --- | --- |
| **Africa** |  |  |  |  |  |  |  |
| Algeria | NA | 7.7 (6.5-8.9) | NA | 33.6 (31.8-35.5) | 31.9 (29.8-34.0) | NA | NA |
| Angola | 32.6 (20.6-44.7) | 32.0 (19.5-44.5) | NA | 68.2 (49.7-86.7) | NA | NA | 34.0 (21.3-46.8) |
| Cameroon | NA | 25.9 (19.7-32.1) | NA | 48.3 (39.6-56.9) | 22.0 (17.4-26.6) | NA | NA |
| Chad | NA | 41.8 (38.4-45.2 | NA | NA | 36.0 (32.7-39.3) | 24.0 (21.1-27.0) | 27.0 (23.9-30.1) |
| Comoros | NA | 34.6 (29.0-40.2) | NA | 44.2 (40.0-48.5) | 23.0 (18.8-27.2) | NA | NA |
| Congo | NA | 36.3 (27.3-45.3) | NA | 62.8 (56.3-69.3) | 19.1 (13.0-25.2) | 30.6 (22.7-38.5) | 27.5 (20.6-34.5) |
| Gabon | NA | 27.2 (23.6-30.7) | NA | 30.1 (25.4-34.9) | 19.3 (15.9-22.6) | NA | NA |
| Gambia | 40.5 (35.1-45.8) | 32.8 (29.0-36.6) | 20.0 (16.8-23.3) | 60.9 (56.9-64.8) | 27.9 (25.4-30.4) | 23.5 (21.2-25.9) | 13.3 (11.3-15.3) |
| Ghana | NA | 26.9 (22.8-31.0) | NA | 49.3 (45.6-53.1) | 19.1 (16.9-21.2) | NA | NA |
| Kenya | NA | 13.1 (8.3-17.8) | NA | 49.2 (44.9-53.5) | 17.2 (13.8-20.6) | NA | NA |
| Madagascar | NA | 6.0 (3.2-8.8) | NA | 52.2 (45.0-59.4) | 9.3 (5.5-13.1) | NA | NA |
| Mauritania | 25.3 (20.8-29.9) | 27.7 (22.5-32.9) | 49.1 (42.7-55.5) | 65.2 (61.0-69.4) | 22.1 (14.9-29.4) | 30.4 (27.1-33.7) | 31.2 (27.7-34.7) |
| Mauritius | 12.6 (8.5-16.7) | 14.2 (9.4-19.1) | 19.0 (16.5-21.5) | 26.9 (22.2-31.6) | 21.0 (17.2-24.9) | 31.4 (25.8-37.0) | 11.0 (8.5-13.6) |
| Mozambique | NA | 26.1 (22.2-30.0) | NA | 41.4 (37.7-45.1) | 24.2 (21.7-26.7) | NA | NA |
| Sao Tome and Principe | 17.2 (15.8-18.6) | 18.5 (17.1-20.0) | 43.5 (41.6-45.3) | 53.2 (51.3-55.0) | 35.0 (33.2-36.8) | 22.1 (20.6-23.7) | 29.6 (27.9-31.2) |
| Senegal | NA | 38.3 (23.5-53.1) | NA | 34.5 (25.0-44.1) | 26.1 (20.6-31.6) | NA | NA |
| Seychelles | NA | 25.5 (22.0-29.0) | NA | 34.0 (30.8-37.1) | 30.4 (27.4-33.4) | NA | 12.3 (10.2-14.3) |
| Sierra Leone | 28.6 (23.7-33.6) | 23.0 (19.0-26.9) | NA | 53.0 (46.9-59.0) | 44.2 (38.0-50.4) | NA | NA |
| South Africa | 23.0 (19.6-26.3) | 23.6 (20.3-26.9) | 15.2 (13.0-17.3) | 42.6 (38.4-46.8) | 46.8 (43.0-50.6) | 59.2 (55.6-62.8) | 24.5 (21.3-27.8) |
| Togo | 22.4 (14.4-30.5) | 17.2 (10.4-23.9) | NA | 48.5 (34.1-62.9) | 7.5 (4.2-10.7) | 14.3 (11.0-17.7) | 10.8 (6.7-14.8) |
| Uganda | 32.3 (21.8-42.9) | 17.8 (11.7-23.9) | 12.9 (7.6-18.2) | 51.3 (47.1-55.4) | 18.1 (13.4-22.7) | 35.3 (27.2-43.4) | 18.5 (13.9-23.1) |
| United Republic of Tanzania | 11.0 (7.9-14.1) | 16.6 (12.3-21.0) | NA | 39.1 (36.2-41.9) | 24.9 (21.1-28.7) | NA | NA |
| Zambia | 35.9 (33.5-38.2) | 40.5 (38.1-42.9) | 22.9 (20.9-25.0) | 37.5 (35.2-39.9) | 34.9 (32.5-37.2) | 40.5 (38.1-42.9) | 30.5 (28.3-32.8) |
| Zimbabwe | 40.5 (34.5-46.4) | 37.3 (29.6-45.0) | NA | 48.8 (43.3-54.4) | 24.0 (18.4-29.6) | 23.0 (20.1-25.9) | 17.9 (13.9-21.9) |
| **Americas** |  |  |  |  |  |  |  |
| Antigua and Barbuda | 11.6 (9.2-14.0) | 12.3 (9.7-14.9) | 15.3 (12.6-18.0) | 25.4 (22.4-28.5) | 38.1 (34.3-41.8) | 30.5 (27.6-33.5) | 8.7 (7.0-10.4) |
| Argentina | 20.6 (11.3-29.9) | 14.4 (9.6-19.3) | NA | 28.8 (20.5-37.1) | 23.4 (16.4-30.3) | 13.3 (8.8-17.8) | 9.2 (3.4-14.9) |
| Bahamas | 10.7 (5.4-16.0) | 14.4 (8.8-20.1) | NA | 48.6 (30.5-66.8) | 28.6 (19.2-38.0) | NA | 21.2 (1.2-41.3) |
| Barbados | NA | 17.3 (14.0-20.5) | NA | 28.2 (25.2-31.1) | 45.3 (40.9-49.6) | NA | NA |
| Belize | NA | 15.4 (12.0-18.8) | NA | 29.5 (26.2-32.8) | 35.7 (31.3-40.1) | NA | NA |
| Bolivia | 24.5 (19.7-29.3) | 17.1 (13.3-20.9) | 23.3 (21.2-25.5) | 39.2 (33.7-44.8) | 35.5 (32.6-38.3) | 30.2 (22.5-37.8) | 13.9 (10.8-17.1) |
| Brazil | 6.8 (5.3-8.3) | 9.7 (7.8-11.6) | 11.9 (9.7-14.0) | 31.6 (27.8-35.4) | NA | NA | NA |
| Chile | NA | 11.6 (9.9-13.2) | 20.5 (18.1-22.9) | 26.4 (24.8-27.9) | 38.9 (37.2-40.5) | NA | NA |
| Costa Rica | 16.2 (13.4-19.0) | 12.8 (10.5-15.1) | 13.8 (11.9-15.7) | 24.6 (21.6-27.6) | 30.9 (27.7-34.1) | 18.7 (16.3-21.1) | 6.7 (5.3-8.1) |
| Cuba | 11.7 (8.8-14.5) | 7.0 (5.1-8.9) | 22.5 (19.7-25.3) | 33.8 (30.5-37.1) | 17.6 (15.0-20.1) | 12.2 (10.6-13.8) | 6.7 (5.1-8.3) |
| Dominican Republic | 14.5 (11.9-17.1) | 27.2 (19.5-34.8) | 61.2 (50.9-71.4) | 39.8 (32.3-47.2) | 34.6 (29.1-40.2) | 25.5 (21.0-30.0) | 11.2 (5.6-16.8) |
| Ecuador | NA | 25.5 (21.3-29.6) | NA | 33.2 (29.5-37.0) | 39.6 (36.6-42.6) | NA | NA |
| El Salvador | NA | 7.1 (5.1-9.0) | NA | 31.0 (27.9-34.1) | 37.3 (34.3-40.4) | NA | NA |
| Grenada | NA | 10.9 (8.6-13.3) | NA | 28.4 (25.2-31.6) | 36.3 (33.0-39.6) | NA | NA |
| Guatemala | 27.9 (24.3-31.6) | 11.8 (9.3-14.3) | 27.0 (24.8-29.3) | 30.9 (27.7-34.1) | 42.0 (38.8-45.1) | 30.0 (26.6-33.3) | 15.2 (12.5-17.8) |
| Guyana | 12.6 (9.6-15.6) | 22.2 (13.4-31.1) | 13.8 (11.9-15.7) | 40.8 (34.6-46.9) | 28.4 (25.8-31.0) | 40.8 (35.6-46.0) | 14.2 (10.6-17.8) |
| Honduras | NA | 24.9 (21.3-28.4) | NA | 37.2 (32.8-41.6) | 23.7 (20.5-27.0) | NA | NA |
| Jamaica | 12.7 (9.1-16.2) | 13.5 (11.3-15.6) | 20.8 (16.6-25.0) | 32.6 (29.6-35.6) | 49.2 (46.0-52.3) | 48.5 (42.6-54.4) | 18.2 (12.4-24.1) |
| Mexico | 6.5 (4.8-8.2) | 6.5 (4.7-8.3) | 25.1 (22.2-28.1) | 38.9 (35.1-42.7) | 33.4 (29.8-37.0) | 33.2 (29.8-36.7) | 22.6 (20.1-25.0) |
| Nicaragua | NA | 11.7 (9.6-13.9) | 16.1 (14.3-17.9) | 39.0 (36.0-41.9) | 24.0 (22.0-26.1) | 27.8 (24.7-31.0) | 10.4 (8.9-11.9) |
| Panama | 12.3 (10.5-14.2) | 8.1 (6.2-9.9) | 21.2 (18.9-23.6) | 42.5 (38.5-46.5) | 23.6 (21.1-26.2) | 19.2 (16.9-21.5) | 12.1 (10.3-14.0) |
| Paraguay | 34.7 (30.2-39.3) | 23.5 (20.5-26.6) | 17.1 (15.3-18.8) | 37.4 (34.5-40.2) | 30.8 (28.2-33.5) | 21.3 (18.8-23.9) | NA |
| Peru | NA | 8.3 (6.3-10.3) | NA | 28.6 (25.4-31.9) | 27.9 (24.6-31.2) | NA | NA |
| Saint Kitts and Nevis | 9.9 (6.1-13.6) | 15.0 (9.7-20.4) | 16.1 (11.4-20.9) | 29.0 (23.2-34.7) | 32.4 (28.0-36.9) | 42.1 (35.4-48.8) | 9.9 (4.8-14.9) |
| Saint Lucia | 17.8 (14.8-20.8) | 16.1 (12.4-19.7) | 17.1 (13.5-20.7) | 35.6 (31.4-39.7) | 35.0 (31.8-38.2) | 32.0 (28.4-35.6) | 11.0 (8.6-13.4) |
| Saint Vincent and the Grenadines | 15.3 (12.2-18.3) | 15.7 (12.5-18.9) | 19.9 (17.2-22.6) | 33.4 (30.2-36.6) | 43.0 (38.5-47.6) | 42.8 (39.8-45.8) | 8.5 (6.6-10.4) |
| Suriname | 8.2 (6.4-10.1) | 9.3 (7.6-10.9) | NA | 24.8 (21.7-28.0) | 47.9 (44.7-51.1) | 42.4 (38.8-45.9) | NA |
| Trinidad and Tobago | 11.4 (8.9-13.9) | 17.3 (14.9-19.7) | 20.2 (17.7-22.7) | 24.9 (21.1-28.7) | 34.8 (31.5-38.1) | 36.1 (32.6-39.6) | 10.2 (8.6-11.8) |
| Uruguay | NA | 8.0 (6.4-9.5) | 19.2 (17.5-21.0) | 30.7 (28.8-32.6) | 25.5 (23.4-27.6) | NA | NA |
| Venezuela | NA | 25.8 (22.1-29.6) | NA | 30.1 (24.7-35.6) | 30.5 (25.9-35.1) | NA | NA |
| **Eastern Mediterranean** |  |  |  |  |  |  |  |
| Afghanistan | 22.0 (8.8-35.3) | 19.6 (15.5-23.7) | 51.6 (39.9-63.2) | 34.2 (29.6-38.9) | 16.6 (12.5-20.8) | 38.3 (22.4-54.1) | 59.9 (47.8-72.0) |
| Bahrain | 24.1 (17.8-30.5) | 20.7 (16.3-25.1) | NA | 34.0 (28.7-39.3) | 26.0 (22.6-29.4) | 30.8 (26.6-35.0) | 22.0 (18.8-25.1) |
| Djibouti | NA | 45.3 (38.1-52.4) | NA | 52.8 (47.8-57.8) | 30.5 (25.4-35.5) | NA | NA |
| Egypt | 13.8 (10.0-17.6) | 17.5 (8.6-26.5) | 18.9 (10.8-27.0) | 50.1 (36.6-63.6) | 22.5 (14.4-30.5) | 37.3 (23.9-50.7) | 15.3 (9.4-21.3) |
| Gaza Strip | NA | 17.2 (12.1-22.3) | NA | 46.6 (41.4-51.7) | 19.6 (15.1-24.1) | NA | 15.9 (10.0-21.8) |
| Iraq | 16.6 (11.2-22.0) | 22.6 (19.6-25.7) | NA | 51.6 (46.9-56.3) | 25.2 (19.1-31.3) | 26.4 (21.9-31.0) | 13.5 (10.4-16.6) |
| Jordan | 13.5 (10.2-16.9) | 13.5 (10.1-17.0) | NA | 32.3 (28.5-36.0) | 24.7 (20.9-28.4) | 36.7 (33.5-39.8) | 19.2 (15.2-23.3) |
| Kuwait | 12.4 (10.3-14.5) | 13.4 (11.6-15.1) | NA | 30.8 (27.5-34.2) | 26.0 (24.1-27.8) | 25.8 (23.0-28.7) | 22.3 (20.0-24.7) |
| Lebanon | 4.9 (3.5-6.3) | 5.5 (3.8-7.2) | NA | 20.2 (17.4-22.9) | 19.1 (17.0-21.1) | 21.3 (17.4-25.2) | 18.3 (15.5-21.2) |
| Libyan Arab Jamahiriya | 8.2 (5.6-10.7) | 14.7 (10.6-18.8) | 43.0 (38.2-47.8) | NA | 17.6 (13.6-21.6) | NA | 21.7 (17.5-25.9) |
| Morocco | 10.0 (8.1-12.0) | 12.7 (10.0-15.4) | 77.5 (74.3-80.8) | 37.9 (33.9-41.9) | 20.4 (15.4-25.4) | NA | 22.0 (18.3-25.8) |
| Oman | 13.0 (8.6-17.5) | 14.6 (9.4-19.9) | 47.1 (38.7-55.6) | 47.7 (41.8-53.7) | 21.4 (15.6-27.1) | 26.3 (22.2-30.4) | 16.9 (14.2-19.7) |
| Pakistan | 20.0 (14.5-25.6) | 12.6 (8.0-17.2) | 36.3 (28.9-43.8) | 61.5 (54.1-68.9) | 61.9 (50.0-73.7) | 20.2 (14.9-25.5) | 38.6 (29.5-47.7) |
| Qatar | NA | 21.6 (18.2-25.0) | NA | 32.0 (27.5-36.4) | 26.2 (23.5-28.8) | NA | NA |
| Saudi Arabia | 8.0 (5.6-10.3) | 15.6 (12.2-19.0) | 51.9 (46.0-57.7) | NA | 22.5 (18.2-26.7) | NA | 28.0 (23.7-32.3) |
| Syrian Arab Republic | 9.9 (6.3-13.5) | 17.1 (10.6-23.7) | 60.3 (53.3-67.2) | NA | 15.5 (12.2-18.8) | NA | 18.9 (14.1-23.6) |
| Tunisia | 4.3 (2.8-5.7) | 9.4 (7.5-11.2) | 48.0 (44.0-52.1) | 40.4 (36.9-43.9) | 21.7 (18.7-24.7) | NA | 21.9 (18.4-25.5) |
| United Arab Emirates | NA | 12.6 (10.5-14.7) | 16.8 (14.9-18.6) | 26.2 (23.2-29.3) | 31.2 (27.4-34.9) | 27.6 (25.0-30.3) | NA |
| UNRWA GAZA | NA | 13.6 (9.6-17.6) | NA | 43.6 (38.5-48.8) | 21.3 (17.5-25.2) | 32.8 (27.1-38.5) | NA |
| UNRWA Jordan | NA | 18.8 (14.6-23.0) | NA | 40.4 (35.7-45.1) | 21.9 (19.5-24.3) | 34.0 (30.2-37.8) | NA |
| UNRWA Lebanon | NA | 13.9 (11.0-16.7) | NA | 34.6 (30.4-38.7) | 20.1 (16.4-23.8) | 30.8 (27.2-34.3) | NA |
| UNRWA West bank | NA | 10.9 (7.8-13.9) | NA | 34.9 (28.8-41.1) | 22.2 (19.1-25.2) | 34.1 (31.6-36.5) | NA |
| West BANK | NA | 17.3 (14.6-20.0) | NA | 39.7 (34.1-45.3) | 21.3 (16.1-26.4) | NA | 19.9 (15.0-24.8) |
| Yemen | NA | 16.8 (11.2-22.4) | NA | 47.2 (42.4-52.0) | 18.0 (14.5-21.6) | NA | 13.0 (10.4-15.6) |
| **Europe** |  |  |  |  |  |  |  |
| Albania | 13.7 (11.3-16.2) | 8.3 (6.7-10.0) | 49.3 (46.5-52.1) | 21.7 (19.3-24.2) | 25.2 (22.8-27.6) | 23.0 (20.6-25.4) | NA |
| Azerbaijan | 11.0 (6.3-15.7) | 11.3 (8.7-14.0) | 37.7 (31.9-43.6) | 60.2 (55.6-64.7) | 29.1 (25.9-32.2) | 22.4 (19.6-25.1) | 32.5 (28.7-36.2) |
| Belarus | 4.6 (3.5-5.6) | 8.2 (7.1-9.4) | NA | 14.8 (12.9-16.7) | 22.0 (19.2-24.8) | NA | 3.6 (2.5-4.8) |
| Bosnia and Herzegovina | 7.5 (6.1-9.0) | 10.5 (9.0-12.0) | NA | 20.4 (17.9-22.9) | 46.6 (44.8-48.3) | 17.1 (15.7-18.6) | 14.8 (13.4-16.2) |
| Bulgaria | NA | 12.8 (10.0-15.7) | NA | 34.4 (31.6-37.2) | 27.9 (26.3-29.5) | NA | NA |
| Croatia (Hrvatska) | 7.1 (5.6-8.7) | 8.9 (7.1-10.7) | 42.1 (40.0-44.2) | 18.2 (16.1-20.4) | 44.7 (41.1-48.3) | 27.3 (23.7-30.9) | 21.8 (18.8-24.8) |
| Cyprus | 9.5 (7.5-11.6) | 12.7 (10.4-15.0) | 24.3 (21.4-27.3) | NA | 24.9 (21.9-27.9) | 32.2 (28.9-35.4) | 23.8 (20.9-26.8) |
| Czech Republic | 7.1 (6.0-8.2) | 10.5 (9.0-11.9) | 22.0 (20.4-23.6) | 11.5 (10.1-12.9) | 40.6 (37.8-43.3) | 34.2 (30.3-38.1) | 13.3 (10.5-16.1) |
| Finland | NA | NA | NA | NA | NA | NA | NA |
| Georgia | 10.5 (7.8-13.1) | 8.2 (5.6-10.7) | NA | 75.8 (71.6-79.9) | 34.0 (29.0-39.1) | 17.5 (12.7-22.3) | 12.1 (9.0-15.1) |
| Greece | NA | 5.3 (4.5-6.2) | NA | 16.8 (15.5-18.2) | 26.4 (24.7-28.1) | NA | NA |
| Italy | 3.7 (2.4-5.1) | 9.9 (8.1-11.6) | NA | 10.8 (8.9-12.8) | 41.3 (37.5-45.0) | 21.5 (18.6-24.4) | 14.3 (11.7-17.0) |
| Kazakhstan | 10.0 (5.0-15.1) | 8.7 (5.3-12.1) | 39.1 (31.0-47.3) | 20.5 (16.2-24.8) | 16.5 (12.9-20.1) | 12.9 (8.7-17.1) | 12.1 (5.3-18.9) |
| Kosovo | 7.1 (5.9-8.3) | 9.2 (8.0-10.4) | NA | 25.9 (23.3-28.6) | 20.4 (18.3-22.5) | 16.7 (14.4-19.0) | 15.9 (14.6-17.3) |
| Kyrgyzstan | NA | 16.5 (13.6-19.4) | NA | 29.0 (24.3-33.6) | 27.2 (24.4-30.0) | NA | NA |
| Latvia | 7.1 (5.4-8.8) | 9.9 (8.4-11.5) | 40.6 (38.9-42.3) | 17.0 (15.0-19.1) | 28.8 (26.6-31.0) | 23.9 (20.9-26.9) | 6.3 (4.6-8.1) |
| Lithuania | 12.4 (10.3-14.5) | 16.2 (13.7-18.6) | NA | 49.3 (46.5-52.1) | 32.2 (29.5-34.8) | 31.9 (30.0-33.8) | 8.2 (6.8-9.6) |
| Macedonia | 10.8 (8.4-13.3) | 11.0 (8.9-13.0) | NA | 25.1 (22.4-27.9) | 23.7 (21.8-25.6) | NA | 13.0 (11.2-14.8) |
| Malta | NA | 6.0 (4.4-7.5) | NA | 14.8 (12.4-17.2) | 45.7 (42.3-49.0) | NA | NA |
| Montenegro | 8.8 (6.9-10.6) | 10.9 (9.5-12.3) | NA | 27.3 (24.8-29.7) | 39.7 (37.4-41.9) | 17.0 (14.9-19.2) | 13.7 (11.9-15.6) |
| Poland | 15.2 (13.4-16.9) | 18.2 (16.2-20.2) | 17.6 (16.3-19.0) | 23.1 (21.0-25.3) | 43.4 (41.4-45.4) | 21.9 (19.7-24.1) | 6.1 (5.0-7.2) |
| Portugal | NA | 13.8 (12.1-15.5) | NA | 22.2 (19.8-24.7) | 33.0 (31.7-34.3) | NA | 12.2 (10.8-13.5) |
| Republic of Moldova | NA | 12.6 (10.4-14.7) | NA | 23.8 (21.5-26.1) | 32.4 (28.9-35.8) | NA | NA |
| Romania | 10.4 (7.7-13.2) | 16.1 (13.8-18.5) | NA | 26.5 (23.9-29.0) | 29.0 (26.9-31.1) | 17.8 (15.8-19.8) | 9.6 (8.4-10.9) |
| Russian Federation | NA | 14.0 (11.8-16.1) | NA | 15.2 (12.8-17.5) | 25.9 (21.2-30.7) | NA | NA |
| San Marino | 5.2 (1.4-9.0) | 12.3 (9.3-15.4) | 23.0 (18.1-27.9) | 8.0 (5.5-10.4) | 45.5 (41.1-49.8) | 18.5 (14.1-22.9) | 14.2 (11.1-17.3) |
| Serbia | 7.5 (6.6-8.4) | 8.7 (7.7-9.7) | NA | 22.5 (21.1-24.0) | 54.6 (52.8-56.4) | NA | NA |
| Slovakia | 6.7 (5.4-7.9) | 10.7 (8.8-12.6) | 20.1 (18.9-21.3) | 21.3 (19.7-22.9) | 49.2 (46.3-52.0) | 20.0 (17.8-22.1) | 10.0 (8.1-11.9) |
| Slovenia | 4.9 (1.8-8.0) | 5.9 (3.3-8.5) | 31.2 (27.2-35.3) | 23.0 (19.6-26.4) | 54.5 (50.5-58.6) | 28.1 (24.6-31.6) | 12.6 (9.3-15.9) |
| Tajikistan | 9.1 (6.2-11.9) | 18.2 (8.8-27.6) | NA | 21.5 (16.5-26.4) | 27.3 (23.7-30.8) | NA | 55.1 (46.5-63.7) |
| Turkey | 9.5 (8.7-10.2) | 8.0 (7.4-8.5) | 68.8 (65.7-71.9) | 36.4 (35.4-37.4) | 27.8 (26.9-28.8) | 33.7 (32.7-34.7) | 17.0 (14.4-19.6) |
| Turkmenistan | NA | 22.1 (7.7-36.5) | NA | 21.1 (10.0-32.3) | 21.9 (-10.1-53.8) | NA | NA |
| Ukraine | 10.0 (7.1-12.9) | 13.6 (10.2-17.1) | 45.8 (40.9-50.8) | 26.3 (22.7-29.9) | 26.9 (21.7-32.2) | 34.3 (29.5-39.1) | 9.9 (7.4-12.3) |
| **South-East Asia** |  |  |  |  |  |  |  |
| Bangladesh | NA | 9.2 (4.1-14.3) | NA | 43.6 (36.9-50.4) | 36.4 (24.5-48.2) | NA | NA |
| Bhutan | NA | 9.7 (8.1-11.3) | NA | 28.8 (25.7-31.9) | 18.6 (16.0-21.1) | NA | NA |
| Indonesia | 6.9 (5.8-7.9) | 5.0 (4.1-5.9) | 29.7 (27.5-31.8) | 18.8 (16.9-20.8) | 13.3 (12.1-14.5) | 20.9 (17.6-24.1) | 6.5 (5.2-7.9) |
| Maldives | 4.8 (3.0-6.5) | 11.3 (9.8-12.9) | NA | 25.2 (22.9-27.5) | 19.4 (17.2-21.5) | 38.0 (34.2-41.7) | 15.8 (13.3-18.4) |
| Myanmar | 8.6 (6.4-10.8) | 4.8 (3.5-6.1) | 48.1 (44.5-51.7) | 32.1 (28.4-35.8) | 29.5 (21.8-37.1) | 41.3 (35.4-47.2) | 36.6 (30.0-43.1) |
| Nepal | 8.6 (6.2-11.0) | 15.0 (11.9-18.1) | 33.9 (28.6-39.3) | 28.4 (25.1-31.8) | 53.9 (49.3-58.4) | 58.3 (53.4-63.2) | 54.5 (50.1-59.0) |
| Sri Lanka | 5.1 (2.4-7.7) | 4.3 (2.9-5.7) | 13.6 (10.0-17.2) | 57.8 (52.8-62.7) | 23.7 (17.0-30.5) | 56.2 (53.7-58.7) | 13.8 (11.9-15.7) |
| Thailand | NA | 6.3 (4.5-8.1) | NA | 45.0 (40.1-49.8) | 28.3 (24.5-32.1) | 26.3 (22.4-30.3) | 9.9 (7.1-12.7) |
| Timor-Leste | 64.1 (55.1-73.1) | 31.4 (27.5-35.3) | NA | 49.8 (46.5-53.2) | 38.2 (35.7-40.6) | NA | NA |
| **Western Pacific** |  |  |  |  |  |  |  |
| Brunei Darussalam | 4.7 (3.2-6.2) | 5.5 (4.0-7.0) | 34.1 (31.0-37.2) | 14.9 (12.9-16.9) | 10.5 (7.6-13.5) | 20.4 (17.7-23.2) | 5.3 (3.6-7.0) |
| Cambodia | 5.4 (3.9-7.0) | 3.5 (2.4-4.6) | 89.9 (86.6-93.3) | 22.0 (19.2-24.9) | 11.1 (8.8-13.4) | 9.7 (7.2-12.2) | 11.5 (8.8-14.2) |
| Cook Islands | 20.7 (16.5-25.0) | 24.5 (19.9-29.0) | 23.1 (18.6-27.5) | 36.9 (31.8-42.0) | 29.3 (24.4-34.2) | 30.2 (25.4-35.1) | 10.0 (6.8-13.2) |
| Fiji | 20.5 (16.1-24.9) | 17.3 (13.7-21.0) | 19.0 (16.1-22.0) | 38.6 (33.7-43.4) | 30.1 (27.2-33.0) | 42.9 (37.3-48.5) | 21.4 (17.8-25.1) |
| Guam | 15.8 (11.7-19.9) | 16.7 (14.4-19.0) | 17.7 (14.2-21.2) | 26.3 (23.1-29.6) | 27.4 (24.9-29.8) | 31.1 (27.2-35.1) | 9.8 (7.0-12.7) |
| Kiribati | 15.5 (12.9-18.1) | 12.2 (10.4-14.0) | 25.4 (22.7-28.2) | 43.3 (40.4-46.2) | 21.6 (19.0-24.2) | NA | NA |
| Laos | 9.9 (7.9-11.9) | 11.7 (7.4-16.1) | 35.6 (30.8-40.3) | 65.4 (61.2-69.5) | 41.6 (38.1-45.1) | 39.1 (34.7-43.5) | 28.5 (24.1-32.9) |
| Macao (China) | 2.6 (1.3-3.9) | 3.4 (2.4-4.5) | 12.4 (10.0-14.8) | 13.7 (10.9-16.6) | 8.0 (2.8-13.2) | 6.1 (4.8-7.4) | 6.3 (5.0-7.6) |
| Marshall Islands | 45.4 (41.3-49.4) | 34.3 (30.5-38.1) | NA | 51.7 (48.3-55.0) | 32.6 (29.9-35.3) | 36.2 (33.3-39.1) | 29.2 (26.3-32.1) |
| Micronesia | 36.0 (33.5-38.4) | 32.6 (30.2-35.1) | 28.3 (26.5-30.0) | 42.3 (40.2-44.4) | 32.6 (30.9-34.3) | 53.3 (51.1-55.5) | 26.5 (24.6-28.4) |
| Mongolia | 6.5 (5.2-7.7) | 9.2 (7.8-10.7) | NA | 56.4 (53.0-59.7) | 5.9 (4.7-7.1) | 19.8 (17.7-21.9) | 3.0 (2.1-3.8) |
| New Caledonia | 3.5 (1.5-5.5) | 9.4 (6.4-12.4) | 56.5 (50.5-62.6) | 18.7 (14.9-22.5) | 47.7 (41.4-53.9) | 35.4 (28.7-42.0) | 19.4 (14.4-24.5) |
| New Zealand | 7.3 (4.2-10.4) | 9.9 (6.8-12.9) | NA | 16.9 (11.8-22.0) | NA | NA | NA |
| Niue | 21.6 (11.1-32.2) | 21.7 (14.1-29.3) | 18.7 (8.0-29.5) | 23.9 (15.7-32.2) | 45.5 (28.4-62.5) | 37.4 (25.4-49.3) | 16.8 (5.1-28.5) |
| Northern Mariana Islands | NA | 11.8 (9.2-14.3) | NA | 26.3 (23.2-29.5) | 32.8 (27.6-38.0) | 33.9 (26.7-41.1) | 10.3 (8.5-12.2) |
| Palau | 14.6 (12.1-17.1) | 13.8 (11.1-16.5) | 21.6 (19.0-24.3) | 24.9 (21.7-28.1) | 27.6 (23.8-31.4) | NA | NA |
| Papua New Guinea | 27.5 (20.6-34.4) | 28.6 (22.7-34.5) | 17.8 (14.9-20.7) | 46.5 (41.7-51.4) | 25.7 (23.1-28.4) | 47.9 (42.6-53.1) | 15.8 (12.9-18.7) |
| Philippines | 16.1 (14.3-17.8) | 13.0 (11.3-14.6) | 18.3 (17.0-19.6) | 34.9 (32.9-36.9) | 17.5 (15.7-19.3) | 34.1 (32.0-36.2) | 13.9 (12.3-15.5) |
| Samoa | 24.2 (19.5-28.9) | 7.8 (6.4-9.2) | NA | 69.7 (63.9-75.6) | 35.0 (31.4-38.5) | NA | NA |
| South Korea | NA | 2.8 (2.3-3.4) | NA | 24.6 (22.6-26.5) | 8.9 (7.8-9.9) | NA | NA |
| Tokelau | 20.4 (6.7-34.1) | 19.9 (9.7-30.0) | 30.9 (20.0-41.7) | 32.5 (21.0-43.9) | 44.3 (30.7-57.8) | 44.2 (31.0-57.5) | 30.8 (14.9-46.7) |
| Tonga | NA | 29.5 (25.3-33.6) | 27.9 (24.1-31.6) | 55.8 (50.9-60.8) | 37.5 (32.5-42.5) | 65.2 (60.1-70.4) | 30.0 (24.1-35.9) |
| Tuvalu | 9.9 (6.2-13.6) | 26.1 (19.2-33.0) | 44.9 (38.1-51.7) | 42.9 (37.0-48.9) | 23.2 (17.2-29.3) | NA | NA |
| Vanuatu | NA | 43.8 (36.5-51.2) | 14.0 (11.0-17.1) | 46.7 (41.6-51.7) | 39.0 (33.6-44.3) | 69.3 (65.1-73.5) | 26.9 (21.3-32.5) |
| Viet Nam | 6.9 (5.3-8.5) | 4.7 (3.6-5.8) | 89.1 (86.4-91.8) | 23.4 (20.8-26.0) | 28.1 (25.2-31.1) | 20.7 (17.7-23.7) | 7.6 (6.1-9.0) |

Abbreviation: NA, not available.

Data are presented as %(95%CI).

Current secondhand smoke exposure was defined as exposure to secondhand smoke in any place (at home or in public places) on at least 1 day during the past 7 days.

**Table S8 Proportions of incorrect beliefs and positive attitudes toward tobacco smoking among adolescents aged 12–16 years who are not exposed to secondhand smoke, by country/territory.**

| **Country/territory** | **Tobacco smoking is not harmful** | **Secondhand smoke exposure is not harmful** | **It is safe to smoke one or two years then quit** | **Once someone smoke, it is easy to quit** | **Tobacco smoking helps people feel more comfortable** | **Tobacco smoking makes more friends** | **Tobacco smoking is more attractive** |
| --- | --- | --- | --- | --- | --- | --- | --- |
| **Africa** |  |  |  |  |  |  |  |
| Algeria | NA | 11.9 (9.6-14.2) | NA | 40.1 (36.5-43.7) | 25.0 (21.7-28.2) | NA | NA |
| Angola | 38.2 (29.4-47.1) | 34.9 (25.7-44.0) | NA | 82.5 (74.5-90.5) | NA | NA | 53.1 (43.0-63.1) |
| Cameroon | NA | 48.7 (40.8-56.5) | NA | 64.2 (56.3-72.1) | 26.0 (18.2-33.9) | NA | NA |
| Chad | NA | 39.2 (35.4-43.0) | NA | NA | 23.6 (20.2-26.9) | 19.6 (16.5-22.8) | 22.0 (18.7-25.3) |
| Comoros | NA | 48.3 (41.4-55.2) | NA | 56.2 (51.3-61.1) | 28.5 (23.2-33.7) | NA | NA |
| Congo | NA | 40.0 (34.7-45.2) | NA | 67.8 (63.1-72.6) | 14.2 (11.9-16.5) | 24.5 (21.8-27.1) | 24.7 (22.1-27.2) |
| Gabon | NA | 29.3 (20.5-38.1) | NA | 30.5 (24.6-36.5) | 16.3 (12.1-20.6) | NA | NA |
| Gambia | 44.4 (37.3-51.4) | 38.7 (32.2-45.2) | 15.8 (13.0-18.7) | 65.1 (61.4-68.9) | 24.5 (21.1-28.0) | 19.2 (16.4-22.0) | 10.4 (8.2-12.6) |
| Ghana | NA | 31.9 (26.9-36.9) | NA | 52.0 (46.8-57.1) | 16.8 (12.7-20.8) | NA | NA |
| Kenya | NA | 21.0 (13.3-28.7) | NA | 55.7 (49.6-61.9) | 12.9 (9.9-15.8) | NA | NA |
| Madagascar | NA | 2.8 (0.3-5.4) | NA | 40.1 (22.9-57.3) | 3.4 (1.4-5.4) | NA | NA |
| Mauritania | 33.2 (25.0-41.4) | 32.6 (25.9-39.2) | 41.0 (34.5-47.5) | 72.2 (67.4-76.9) | 19.2 (15.3-23.1) | 23.8 (20.9-26.6) | 25.1 (21.9-28.2) |
| Mauritius | 15.8 (10.2-21.4) | 17.7 (11.7-23.7) | 14.8 (12.0-17.6) | 30.9 (26.0-35.8) | 11.4 (8.8-14.0) | 21.5 (16.3-26.8) | 10.4 (6.6-14.1) |
| Mozambique | NA | 36.3 (31.2-41.5) | NA | 53.1 (48.8-57.3) | 21.8 (18.3-25.3) | NA | NA |
| Sao Tome and Principe | 22.7 (21.1-24.2) | 26.0 (24.4-27.7) | 38.6 (36.8-40.4) | 61.7 (59.9-63.5) | 32.9 (31.1-34.7) | 19.4 (17.9-20.8) | 28.9 (27.2-30.6) |
| Senegal | NA | 65.0 (49.7-80.3) | NA | 57.2 (49.5-65.0) | 21.3 (14.4-28.2) | NA | NA |
| Seychelles | NA | 34.4 (28.9-39.9) | NA | 46.6 (40.8-52.5) | 21.2 (17.9-24.6) | NA | 14.6 (11.8-17.5) |
| Sierra Leone | 36.1 (26.9-45.3) | 25.6 (16.3-34.9) | NA | 68.0 (59.8-76.3) | 30.5 (22.0-38.9) | NA | NA |
| South Africa | 35.9 (30.4-41.3) | 40.2 (35.5-44.8) | 11.5 (9.6-13.3) | 57.4 (51.1-63.8) | 46.0 (42.3-49.6) | 57.3 (51.9-62.6) | 27.5 (24.2-30.7) |
| Togo | 32.1 (19.2-44.9) | 27.1 (18.1-36.0) | NA | 47.7 (38.8-56.5) | 8.3 (3.7-12.8) | 14.3 (10.5-18.2) | 11.2 (8.0-14.5) |
| Uganda | 35.6 (25.5-45.7) | 23.9 (17.3-30.6) | 7.2 (2.8-11.5) | 55.3 (48.1-62.6) | 15.7 (12.9-18.4) | 25.9 (21.6-30.2) | 14.9 (10.3-19.6) |
| United Republic of Tanzania | 12.6 (9.8-15.5) | 18.4 (13.2-23.6) | NA | 48.9 (44.4-53.5) | 22.4 (18.0-26.8) | NA | NA |
| Zambia | 39.8 (37.0-42.6) | 45.1 (42.3-48.0) | 11.2 (9.4-13.0) | 46.3 (43.4-49.1) | 27.4 (24.8-30.1) | 34.5 (31.7-37.2) | 21.9 (19.5-24.3) |
| Zimbabwe | 51.0 (40.6-61.3) | 49.4 (39.9-58.8) | NA | 65.0 (58.2-71.9) | 18.1 (14.8-21.4) | 20.3 (13.3-27.4) | 15.2 (10.0-20.5) |
| **Americas** |  |  |  |  |  |  |  |
| Antigua and Barbuda | 16.8 (13.5-20.1) | 15.7 (12.7-18.7) | 10.7 (8.6-12.7) | 32.9 (29.1-36.7) | 31.3 (27.8-34.8) | 24.4 (21.3-27.4) | 7.4 (5.3-9.6) |
| Argentina | 24.8 (17.3-32.3) | 20.1 (14.4-25.8) | NA | 38.4 (30.4-46.4) | 26.5 (20.8-32.3) | 11.6 (8.8-14.4) | 7.5 (4.3-10.8) |
| Bahamas | 20.1 (11.3-28.9) | 21.9 (16.2-27.6) | NA | 32.6 (24.4-40.9) | 27.5 (23.3-31.6) | NA | 7.7 (4.2-11.1) |
| Barbados | NA | 17.3 (14.1-20.5) | NA | 33.5 (28.9-38.2) | 39.7 (35.9-43.5) | NA | NA |
| Belize | NA | 16.6 (13.5-19.6) | NA | 33.4 (28.7-38.2) | 29.2 (25.5-32.8) | NA | NA |
| Bolivia | 28.4 (25.1-31.7) | 21.5 (19.1-24.0) | 16.8 (15.2-18.4) | 49.6 (46.1-53.0) | 27.3 (24.9-29.8) | 27.1 (22.1-32.0) | 14.5 (12.1-16.9) |
| Brazil | 8.9 (6.6-11.3) | 10.4 (8.1-12.6) | 7.3 (5.7-8.9) | 39.2 (35.7-42.7) | NA | NA | NA |
| Chile | NA | 11.1 (8.7-13.5) | 14.1 (11.6-16.6) | 31.6 (29.1-34.1) | 33.6 (31.3-35.9) | NA | NA |
| Costa Rica | 22.5 (18.7-26.4) | 17.8 (14.5-21.1) | 11.2 (9.6-12.8) | 34.6 (30.0-39.2) | 24.0 (20.7-27.2) | 18.7 (15.3-22.1) | 6.8 (4.7-9.0) |
| Cuba | 23.7 (19.3-28.0) | 15.3 (12.8-17.7) | 14.1 (12.2-16.1) | 51.6 (49.1-54.2) | 17.4 (15.5-19.4) | 13.4 (10.9-15.8) | 8.6 (6.8-10.4) |
| Dominican Republic | 18.1 (6.7-29.5) | 43.1 (34.8-51.3) | 64.6 (60.4-68.8) | 48.2 (41.1-55.2) | 30.3 (24.1-36.5) | 18.8 (11.0-26.6) | 8.7 (3.4-13.9) |
| Ecuador | NA | 37.9 (33.6-42.2) | NA | 50.1 (46.4-53.7) | 32.2 (28.5-36.0) | NA | NA |
| El Salvador | NA | 6.7 (5.3-8.0) | NA | 36.1 (32.3-40.0) | 27.2 (23.5-31.0) | NA | NA |
| Grenada | NA | 14.8 (11.3-18.3) | NA | 29.8 (25.4-34.2) | 25.3 (21.4-29.2) | NA | NA |
| Guatemala | 34.4 (30.6-38.2) | 14.2 (11.8-16.7) | 22.0 (19.8-24.2) | 42.1 (37.5-46.7) | 30.4 (27.5-33.3) | 31.0 (27.8-34.2) | 14.9 (12.2-17.6) |
| Guyana | 18.9 (12.8-25.0) | 29.8 (23.6-35.9) | 10.7 (8.3-13.2) | 53.9 (46.9-60.9) | 26.8 (21.2-32.4) | 43.3 (37.3-49.4) | 14.8 (9.9-19.7) |
| Honduras | NA | 29.5 (25.3-33.7) | NA | 48.7 (44.3-53.1) | 19.5 (16.4-22.6) | NA | NA |
| Jamaica | 16.1 (8.3-23.8) | 18.8 (15.1-22.4) | 11.4 (8.8-14.0) | 38.0 (33.4-42.6) | 37.8 (33.1-42.5) | 39.4 (32.9-46.0) | 10.8 (6.0-15.6) |
| Mexico | 6.0 (2.6-9.3) | 8.5 (5.7-11.2) | 21.1 (17.7-24.6) | 40.5 (36.3-44.7) | 27.7 (23.2-32.2) | 29.4 (26.3-32.6) | 17.7 (14.2-21.3) |
| Nicaragua | NA | 12.8 (11.0-14.6) | 9.1 (8.0-10.2) | 48.5 (45.3-51.6) | 19.7 (17.9-21.4) | 23.6 (21.8-25.5) | 10.7 (9.2-12.2) |
| Panama | 16.0 (13.4-18.6) | 10.1 (8.1-12.2) | 18.6 (16.9-20.4) | 52.5 (47.3-57.7) | 19.8 (16.8-22.8) | 14.8 (12.8-16.7) | 11.4 (8.7-14.1) |
| Paraguay | 41.7 (35.8-47.7) | 35.6 (31.3-40.0) | 15.5 (13.8-17.3) | 45.2 (40.2-50.1) | 27.5 (24.0-30.9) | 19.0 (16.1-21.9) | NA |
| Peru | NA | 10.6 (8.0-13.2) | NA | 37.5 (34.6-40.3) | 20.3 (17.7-22.9) | NA | NA |
| Saint Kitts and Nevis | 14.1 (8.8-19.5) | 20.6 (14.6-26.6) | 11.7 (7.8-15.6) | 33.3 (26.0-40.7) | 25.5 (20.4-30.6) | 37.1 (28.6-45.7) | 10.8 (5.9-15.7) |
| Saint Lucia | 17.9 (13.7-22.2) | 16.6 (12.5-20.6) | 10.0 (6.5-13.4) | 43.9 (39.2-48.6) | 31.9 (27.3-36.4) | 24.1 (19.9-28.4) | 10.9 (7.5-14.2) |
| Saint Vincent and the Grenadines | 17.9 (12.6-23.2) | 17.6 (13.4-21.8) | 11.9 (8.1-15.8) | 38.3 (32.5-44.1) | 35.3 (29.6-41.0) | 35.9 (30.2-41.5) | 6.8 (4.2-9.5) |
| Suriname | 11.0 (8.1-13.9) | 9.5 (5.4-13.6) | NA | 29.2 (25.6-32.8) | 40.6 (35.1-46.1) | 39.2 (34.0-44.4) | NA |
| Trinidad and Tobago | 17.2 (12.9-21.4) | 23.0 (19.0-27.1) | 11.8 (9.7-14.0) | 34.9 (29.4-40.4) | 28.7 (24.6-32.7) | 31.2 (27.3-35.1) | 8.8 (5.6-12.0) |
| Uruguay | NA | 12.6 (10.6-14.5) | 13.6 (11.8-15.4) | 37.8 (34.9-40.7) | 18.6 (16.0-21.2) | NA | NA |
| Venezuela | NA | 36.3 (31.1-41.5) | NA | 50.7 (45.0-56.3) | 20.7 (17.7-23.7) | NA | NA |
| **Eastern Mediterranean** |  |  |  |  |  |  |  |
| Afghanistan | 20.1 (10.4-29.9) | 25.9 (19.7-32.1) | 50.0 (36.2-63.8) | 41.4 (35.9-46.8) | 19.6 (15.6-23.6) | 30.9 (17.7-44.1) | 51.5 (41.9-61.1) |
| Bahrain | 24.2 (16.5-31.9) | 22.7 (17.6-27.8) | NA | 37.7 (32.6-42.7) | 17.9 (14.8-21.0) | 27.6 (24.2-31.1) | 19.4 (15.2-23.5) |
| Djibouti | NA | 59.3 (50.0-68.7) | NA | 66.0 (59.4-72.6) | 35.3 (26.7-43.8) | NA | NA |
| Egypt | 15.8 (5.2-26.4) | 12.8 (5.6-20.0) | 22.0 (5.8-38.2) | 75.6 (64.6-86.6) | 14.3 (6.2-22.3) | 25.7 (14.9-36.6) | 9.6 (0.6-18.6) |
| Gaza Strip | NA | 25.3 (16.6-34.1) | NA | 56.4 (48.0-64.8) | 17.0 (10.4-23.6) | NA | 16.3 (8.9-23.7) |
| Iraq | 24.1 (16.9-31.3) | 28.6 (23.0-34.2) | NA | 60.8 (55.4-66.2) | 19.9 (17.3-22.4) | 24.4 (16.2-32.6) | 13.1 (7.3-19.0) |
| Jordan | 22.4 (16.5-28.4) | 27.0 (18.8-35.2) | NA | 50.4 (43.3-57.5) | 23.5 (17.3-29.8) | 36.0 (30.0-42.1) | 20.2 (14.5-26.0) |
| Kuwait | 14.1 (8.2-19.9) | 15.8 (11.3-20.3) | NA | 35.4 (29.2-41.6) | 16.5 (13.0-20.0) | 22.7 (17.5-28.0) | 15.8 (11.6-19.9) |
| Lebanon | 7.1 (4.1-10.1) | 5.9 (3.0-8.8) | NA | 27.9 (21.9-33.9) | 16.8 (11.7-21.9) | 19.1 (15.4-22.8) | 12.9 (8.5-17.2) |
| Libyan Arab Jamahiriya | 7.8 (6.0-9.6) | 22.1 (18.7-25.5) | 37.3 (33.2-41.3) | NA | 9.8 (7.0-12.6) | NA | 18.0 (14.7-21.2) |
| Morocco | 13.6 (10.3-16.9) | 18.1 (15.7-20.6) | 75.3 (72.6-78.0) | 51.9 (48.8-55.0) | 15.3 (12.6-18.1) | NA | 18.3 (15.6-20.9) |
| Oman | 9.2 (6.5-12.0) | 15.3 (7.5-23.1) | 45.4 (41.8-49.0) | 48.0 (41.2-54.7) | 14.7 (11.1-18.4) | 20.8 (17.0-24.5) | 15.3 (10.4-20.1) |
| Pakistan | 25.5 (19.0-32.0) | 12.9 (9.6-16.2) | 27.8 (24.2-31.4) | 60.9 (56.5-65.3) | 63.0 (57.3-68.7) | 18.9 (15.7-22.2) | 39.8 (34.1-45.6) |
| Qatar | NA | 26.2 (22.1-30.3) | NA | 42.1 (34.6-49.7) | 18.0 (13.9-22.1) | NA | NA |
| Saudi Arabia | 6.4 (4.2-8.7) | 15.1 (11.8-18.5) | 38.8 (34.3-43.2) | NA | 8.5 (6.1-10.8) | NA | 22.7 (17.2-28.3) |
| Syrian Arab Republic | 11.5 (8.3-14.6) | 22.7 (17.3-28.2) | 46.8 (42.0-51.5) | NA | 13.6 (9.8-17.5) | NA | 17.6 (13.8-21.5) |
| Tunisia | 9.0 (5.4-12.5) | 24.1 (19.0-29.2) | 36.6 (32.1-41.1) | 53.7 (47.7-59.8) | 16.5 (12.8-20.2) | NA | 19.7 (15.7-23.8) |
| United Arab Emirates | NA | 16.5 (13.5-19.5) | 10.5 (8.7-12.4) | 34.1 (30.1-38.1) | 20.3 (17.5-23.1) | 21.7 (19.6-23.7) | NA |
| UNRWA GAZA | NA | 22.9 (14.6-31.2) | NA | 47.6 (38.1-57.1) | 14.9 (8.5-21.4) | 28.9 (21.9-36.0) | NA |
| UNRWA Jordan | NA | 25.7 (19.6-31.9) | NA | 51.8 (46.2-57.4) | 18.6 (9.8-27.3) | 32.9 (22.0-43.8) | NA |
| UNRWA Lebanon | NA | 19.5 (11.3-27.7) | NA | 54.1 (44.2-64.0) | 16.2 (9.6-22.9) | 30.3 (24.7-35.8) | NA |
| UNRWA West bank | NA | 26.4 (16.5-36.3) | NA | 45.8 (34.8-56.7) | 20.6 (9.5-31.7) | 30.7 (22.5-39.0) | NA |
| West BANK | NA | 17.9 (12.0-23.7) | NA | 52.4 (43.5-61.2) | 19.3 (11.5-27.2) | NA | 18.0 (12.8-23.2) |
| Yemen | NA | 22.2 (13.7-30.6) | NA | 46.9 (33.5-60.3) | 18.1 (10.4-25.8) | NA | 15.6 (7.6-23.7) |
| **Europe** |  |  |  |  |  |  |  |
| Albania | 19.6 (16.8-22.3) | 15.2 (12.5-17.9) | 44.3 (42.1-46.6) | 34.4 (31.5-37.4) | 23.5 (20.6-26.4) | 20.4 (18.0-22.8) | NA |
| Azerbaijan | 9.1 (4.6-13.5) | 8.7 (5.8-11.5) | 35.8 (30.7-41.0) | 58.9 (52.8-65.0) | 20.6 (16.8-24.4) | 14.9 (11.3-18.5) | 24.7 (19.4-29.9) |
| Belarus | 5.2 (3.3-7.2) | 8.7 (6.0-11.3) | NA | 16.4 (13.4-19.5) | 16.8 (13.6-19.9) | NA | 3.1 (1.8-4.4) |
| Bosnia and Herzegovina | 9.4 (6.7-12.1) | 11.3 (8.9-13.7) | NA | 24.0 (20.3-27.8) | 41.8 (37.0-46.5) | 14.2 (12.4-16.1) | 15.9 (13.8-17.9) |
| Bulgaria | NA | 22.0 (15.7-28.4) | NA | 44.0 (37.3-50.7) | 36.1 (30.0-42.2) | NA | NA |
| Croatia (Hrvatska) | 6.8 (3.3-10.2) | 10.6 (5.5-15.7) | 40.2 (33.1-47.4) | 20.5 (14.5-26.4) | 38.1 (30.2-46.1) | 23.8 (16.9-30.7) | 18.1 (12.6-23.7) |
| Cyprus | 21.1 (15.6-26.6) | 26.1 (20.3-32.0) | 15.1 (10.3-19.9) | NA | 26.5 (20.5-32.5) | 30.7 (24.6-36.9) | 31.2 (25.0-37.4) |
| Czech Republic | 6.8 (4.5-9.1) | 8.4 (6.0-10.7) | 16.3 (10.9-21.8) | 11.8 (8.9-14.7) | 36.6 (31.9-41.3) | 31.0 (26.0-36.0) | 10.7 (5.1-16.3) |
| Finland | NA | NA | NA | NA | NA | NA | NA |
| Georgia | 14.0 (9.3-18.7) | 12.5 (8.8-16.2) | NA | 78.0 (70.7-85.3) | 27.3 (17.9-36.7) | 12.7 (6.8-18.6) | 9.5 (6.0-13.0) |
| Greece | NA | 10.4 (6.6-14.3) | NA | 26.4 (21.8-30.9) | 21.2 (18.2-24.2) | NA | NA |
| Italy | 2.9 (0.0-5.7) | 9.4 (4.4-14.4) | NA | 11.6 (5.4-17.9) | 37.7 (29.4-46.0) | 17.0 (9.3-24.7) | 11.1 (6.2-15.9) |
| Kazakhstan | 7.5 (4.0-11.0) | 8.9 (5.5-12.4) | 48.1 (41.0-55.3) | 26.5 (21.0-31.9) | 9.6 (7.0-12.2) | 9.5 (6.1-13.0) | 12.4 (8.0-16.8) |
| Kosovo | 11.3 (9.0-13.5) | 15.1 (13.4-16.8) | NA | 36.0 (33.3-38.7) | 22.7 (19.4-26.0) | 12.5 (10.1-14.9) | 16.7 (14.5-18.9) |
| Kyrgyzstan | NA | 11.0 (9.2-12.8) | NA | 36.8 (33.8-39.8) | 28.3 (24.6-32.0) | NA | NA |
| Latvia | 9.2 (6.4-12.0) | 9.1 (6.0-12.2) | 37.0 (33.1-40.8) | 20.6 (15.9-25.2) | 20.1 (17.3-23.0) | 24.2 (22.0-26.4) | 9.4 (5.4-13.4) |
| Lithuania | 18.9 (14.8-22.9) | 21.9 (18.8-25.0) | NA | 58.9 (53.1-64.7) | 26.0 (21.4-30.6) | 34.5 (30.2-38.9) | 11.3 (8.1-14.4) |
| Macedonia | 17.3 (14.1-20.4) | 11.2 (8.7-13.7) | NA | 36.0 (31.9-40.2) | 21.5 (19.4-23.6) | NA | 17.4 (15.0-19.7) |
| Malta | NA | 13.2 (9.8-16.7) | NA | 20.9 (16.8-25.0) | 29.0 (24.4-33.7) | NA | NA |
| Montenegro | 10.8 (7.2-14.3) | 14.2 (11.9-16.6) | NA | 33.5 (29.8-37.2) | 30.6 (27.5-33.7) | 15.1 (12.0-18.2) | 13.2 (10.6-15.9) |
| Poland | 19.3 (14.6-24.0) | 20.6 (17.3-23.9) | 13.2 (10.4-16.1) | 30.1 (25.0-35.1) | 34.5 (30.6-38.3) | 25.6 (20.7-30.6) | 9.1 (5.3-13.0) |
| Portugal | NA | 10.4 (8.2-12.6) | NA | 24.4 (21.0-27.7) | 25.7 (23.8-27.6) | NA | 8.2 (7.1-9.4) |
| Republic of Moldova | NA | 18.8 (15.8-21.7) | NA | 36.0 (31.9-40.1) | 25.5 (22.1-28.8) | NA | NA |
| Romania | 14.2 (11.7-16.8) | 20.7 (17.8-23.7) | NA | 31.8 (28.8-34.8) | 19.7 (17.2-22.1) | 16.0 (14.2-17.9) | 10.2 (8.3-12.0) |
| Russian Federation | NA | 11.7 (8.3-15.1) | NA | 17.0 (13.3-20.6) | 22.2 (16.9-27.5) | NA | NA |
| San Marino | 6.5 (0.6-12.4) | 16.3 (9.6-23.1) | 16.8 (9.1-24.4) | 4.2 (-0.1-8.5) | 41.4 (33.1-49.8) | 16.9 (10.7-23.2) | 11.6 (5.6-17.6) |
| Serbia | 10.3 (8.0-12.6) | 12.4 (9.9-14.9) | NA | 31.7 (28.2-35.2) | 48.7 (44.9-52.5) | NA | NA |
| Slovakia | 7.4 (5.1-9.7) | 9.5 (7.7-11.4) | 13.2 (10.9-15.4) | 21.0 (18.1-23.8) | 43.7 (39.4-47.9) | 17.2 (14.5-19.9) | 9.4 (7.1-11.7) |
| Slovenia | 4.7 (2.4-7.1) | 9.2 (6.7-11.8) | 21.8 (18.5-25.1) | 24.4 (21.1-27.7) | 51.3 (47.2-55.4) | 27.3 (23.0-31.5) | 7.9 (4.6-11.2) |
| Tajikistan | 9.5 (7.1-11.8) | 17.5 (10.0-25.0) | NA | 26.8 (22.2-31.3) | 23.5 (20.5-26.5) | NA | 50.9 (43.5-58.4) |
| Turkey | 10.3 (9.1-11.6) | 10.1 (9.4-10.9) | 74.0 (71.3-76.8) | 43.8 (42.7-45.0) | 27.3 (26.2-28.3) | 29.6 (28.3-30.9) | 14.5 (10.3-18.7) |
| Turkmenistan | NA | 2.7 (1.7-3.7) | NA | 6.6 (4.1-9.1) | 7.3 (3.2-11.4) | NA | NA |
| Ukraine | 13.6 (8.8-18.3) | 21.0 (14.7-27.4) | 48.2 (40.6-55.8) | 30.7 (25.7-35.7) | 21.0 (14.0-28.1) | 30.2 (26.0-34.4) | 7.2 (5.1-9.4) |
| **South-East Asia** |  |  |  |  |  |  |  |
| Bangladesh | NA | 9.3 (4.9-13.8) | NA | 48.4 (38.0-58.9) | 25.1 (17.0-33.1) | NA | NA |
| Bhutan | NA | 11.3 (8.7-14.0) | NA | 31.9 (28.4-35.5) | 14.5 (12.2-16.7) | NA | NA |
| Indonesia | 10.3 (8.0-12.6) | 9.0 (6.5-11.5) | 21.6 (18.2-24.9) | 26.9 (22.8-31.0) | 10.3 (8.5-12.1) | 15.3 (9.5-21.2) | 4.2 (2.2-6.2) |
| Maldives | 6.2 (4.2-8.3) | 20.2 (16.5-24.0) | NA | 37.0 (32.8-41.2) | 16.8 (14.4-19.3) | 34.1 (27.0-41.2) | 12.5 (9.8-15.2) |
| Myanmar | 4.7 (3.5-5.9) | 4.6 (3.2-6.0) | 51.1 (46.9-55.4) | 25.7 (22.3-29.1) | 22.4 (19.0-25.9) | 39.4 (35.0-43.9) | 38.6 (32.8-44.4) |
| Nepal | 14.8 (9.6-20.0) | 24.0 (18.8-29.2) | 30.9 (25.5-36.2) | 32.9 (25.9-40.0) | 53.3 (45.5-61.1) | 50.9 (43.7-58.1) | 49.9 (43.8-56.0) |
| Sri Lanka | 8.0 (5.3-10.6) | 8.4 (5.3-11.4) | 12.3 (10.0-14.6) | 67.9 (60.6-75.1) | 24.7 (18.5-31.0) | 49.5 (46.1-53.0) | 12.3 (10.0-14.5) |
| Thailand | NA | 13.3 (10.7-16.0) | NA | 51.1 (45.1-57.2) | 27.8 (25.3-30.4) | 20.9 (16.7-25.0) | 14.5 (11.1-17.9) |
| Timor-Leste | 81.3 (77.5-85.1) | 58.2 (51.5-64.9) | NA | 72.2 (66.4-78.1) | 43.9 (36.2-51.7) | NA | NA |
| **Western Pacific** |  |  |  |  |  |  |  |
| Brunei Darussalam | 6.9 (3.2-10.6) | 7.3 (3.4-11.2) | 23.0 (18.8-27.3) | 16.7 (11.4-22.1) | 10.9 (6.6-15.1) | 15.1 (12.0-18.1) | 5.0 (2.4-7.6) |
| Cambodia | 9.4 (6.8-11.9) | 10.5 (8.5-12.4) | 87.6 (85.0-90.2) | 27.5 (23.1-31.8) | 10.3 (7.4-13.2) | 8.9 (6.4-11.4) | 12.2 (8.6-15.7) |
| Cook Islands | 21.5 (14.5-28.5) | 30.4 (22.5-38.2) | 17.8 (11.2-24.3) | 34.8 (26.7-43.0) | 30.8 (22.9-38.8) | 20.1 (13.3-27.0) | 10.5 (5.2-15.8) |
| Fiji | 24.4 (16.7-32.2) | 22.8 (18.6-27.1) | 16.2 (10.9-21.4) | 42.8 (37.1-48.5) | 21.7 (15.6-27.7) | 35.3 (30.7-40.0) | 15.4 (9.9-20.8) |
| Guam | 15.6 (11.4-19.9) | 21.8 (17.8-25.8) | 10.3 (3.5-17.0) | 33.3 (28.6-38.0) | 24.7 (20.3-29.1) | 31.2 (24.9-37.6) | 12.4 (8.4-16.4) |
| Kiribati | 19.3 (14.6-24.1) | 19.5 (14.1-24.9) | 21.6 (17.5-25.6) | 46.8 (41.7-52.0) | 27.6 (23.2-31.9) | NA | NA |
| Laos | 14.9 (12.4-17.4) | 18.0 (15.1-20.9) | 25.1 (22.8-27.5) | 72.5 (68.4-76.7) | 42.1 (38.6-45.6) | 30.6 (27.2-33.9) | 23.9 (21.5-26.4) |
| Macao (China) | 6.8 (4.4-9.1) | 6.3 (3.4-9.3) | 14.8 (11.1-18.6) | 19.2 (15.4-23.1) | 7.7 (4.6-10.8) | 6.8 (4.6-8.9) | 7.2 (5.2-9.1) |
| Marshall Islands | 60.5 (56.3-64.6) | 53.7 (49.4-57.9) | NA | 61.8 (58.0-65.7) | 34.2 (30.3-38.1) | 34.3 (30.5-38.2) | 31.6 (27.9-35.4) |
| Micronesia | 48.2 (44.5-51.9) | 46.7 (43.2-50.2) | 22.7 (20.0-25.4) | 57.2 (54.2-60.2) | 31.6 (28.7-34.6) | 46.4 (43.8-49.1) | 29.7 (27.0-32.4) |
| Mongolia | 13.2 (10.7-15.7) | 19.3 (15.8-22.8) | NA | 71.8 (67.7-75.9) | 9.6 (6.7-12.5) | 14.9 (11.5-18.4) | 4.9 (3.5-6.3) |
| New Caledonia | 3.9 (0.2-7.7) | 11.8 (1.7-22.0) | 62.8 (55.7-70.0) | 23.7 (16.0-31.5) | 49.0 (39.5-58.5) | 28.7 (19.9-37.5) | 8.6 (2.8-14.4) |
| New Zealand | 7.0 (3.9-10.1) | 6.1 (3.0-9.2) | NA | 13.8 (9.5-18.1) | NA | NA | NA |
| Niue | 31.3 (14.3-48.4) | 26.8 (12.8-40.8) | 12.4 (1.5-23.3) | 25.1 (13.0-37.3) | 29.6 (14.1-45.2) | 23.4 (9.8-36.9) | 7.7 (-3.5-18.9) |
| Northern Mariana Islands | NA | 17.4 (14.3-20.6) | NA | 31.0 (26.5-35.6) | 19.6 (16.3-23.0) | 20.4 (17.3-23.4) | 5.3 (3.2-7.4) |
| Palau | 14.1 (10.2-18.0) | 18.1 (13.2-23.0) | 13.0 (9.3-16.6) | 33.2 (26.9-39.5) | 23.1 (17.9-28.3) | NA | NA |
| Papua New Guinea | 50.0 (40.9-59.1) | 52.3 (41.5-63.2) | 11.4 (6.9-15.9) | 60.2 (53.9-66.5) | 23.7 (18.1-29.4) | 35.9 (29.0-42.8) | 22.0 (15.3-28.8) |
| Philippines | 24.8 (22.0-27.6) | 21.0 (18.7-23.4) | 14.7 (12.8-16.7) | 50.4 (47.2-53.6) | 15.8 (14.0-17.6) | 31.0 (28.4-33.6) | 16.0 (13.4-18.5) |
| Samoa | 31.4 (22.9-39.9) | 16.3 (10.6-21.9) | NA | 72.6 (62.8-82.4) | 31.5 (26.7-36.4) | NA | NA |
| South Korea | NA | 4.6 (3.7-5.6) | NA | 32.6 (29.7-35.6) | 10.7 (8.5-12.9) | NA | NA |
| Tokelau | NA | NA | NA | NA | NA | NA | NA |
| Tonga | NA | 36.4 (29.7-43.0) | 20.1 (14.8-25.4) | 60.2 (52.7-67.6) | 32.3 (26.5-38.2) | 51.3 (44.6-58.0) | 20.6 (12.6-28.6) |
| Tuvalu | 17.3 (11.4-23.2) | 39.4 (30.6-48.3) | 41.5 (33.8-49.2) | 46.1 (36.2-56.0) | 26.8 (18.9-34.7) | NA | NA |
| Vanuatu | NA | 50.4 (42.7-58.1) | 10.2 (4.0-16.3) | 50.2 (43.1-57.4) | 33.1 (26.9-39.2) | 63.9 (57.4-70.5) | 25.2 (18.5-32.0) |
| Viet Nam | 5.8 (3.5-8.2) | 7.2 (4.4-9.9) | 90.0 (87.2-92.8) | 28.3 (24.1-32.5) | 21.0 (14.9-27.1) | 16.0 (12.9-19.1) | 6.7 (3.8-9.6) |

Abbreviation: NA, not available.

Data are presented as %(95%CI).

Current secondhand smoke exposure was defined as exposure to secondhand smoke in any place (at home or in public places) on at least 1 day during the past 7 days.

**Table S9 Proportions of incorrect beliefs and positive attitudes toward tobacco smoking among adolescents aged 12–16 years who are not exposed to secondhand smoke, stratified by age, sex, WHO region, World Bank income category, current tobacco use status, FCTC ratification, tobacco use monitoring, tobacco warning policies, and enforcement of tobacco advertising bans.**

|  | **Tobacco smoking is not harmful** | **Secondhand smoke exposure is not harmful** | **It is safe to smoke one or two years then quit** | **Once someone smoke, it is easy to quit** | **Tobacco smoking helps people feel more comfortable** | **Tobacco smoking makes more friends** | **Tobacco smoking is more attractive** |
| --- | --- | --- | --- | --- | --- | --- | --- |
| **Total** | 12.7 (10.2-15.2) | 15.0 (14.1-16.0) | 41.4 (34.6-48.2) | 40.0 (38.9-41.2) | 24.6 (23.5-25.7) | 25.8 (24.0-27.6) | 15.0 (13.6-16.3) |
| **Sex** |  |  |  |  |  |  |  |
| Boys | 14.7 (13.1-16.2) | 15.3 (14.0-16.5) | 41.9 (38.5-45.3) | 40.0 (38.3-41.6) | 29.5 (27.7-31.3) | 29.5 (27.5-31.5) | 19.3 (17.9-20.8) |
| Girls | 9.6 (8.2-11.1) | 11.6 (10.5-12.6) | 40.5 (33.6-47.4) | 31.8 (30.0-33.7) | 25.2 (23.7-26.8) | 28.5 (25.5-31.5) | 14.2 (12.0-16.3) |
| *P* value | <0.001 | <0.001 | 0.58 | <0.001 | <0.001 | 0.59 | <0.001 |
| **Age group** |  |  |  |  |  |  |  |
| 12-14 years | 12.9 (11.4-14.3) | 13.3 (12.2-14.4) | 42.8 (37.3-48.3) | 35.4 (33.7-37.1) | 26.4 (24.7-28.1) | 27.4 (25.3-29.6) | 15.7 (14.0-17.3) |
| 15-16 years | 10.7 (9.5-11.9) | 13.8 (12.7-14.9) | 37.8 (33.6-42.0) | 37.1 (35.7-38.5) | 29.2 (28.0-30.5) | 32.4 (30.0-34.9) | 19.1 (16.7-21.4) |
| *P* value | 0.05 | 0.42 | 0.02 | 0.08 | 0.002 | 0.001 | 0.02 |
| **WHO region** |  |  |  |  |  |  |  |
| Africa | 20.5 (17.4-23.7) | 19.9 (17.7-22.1) | 16.5 (14.0-19.0) | 44.6 (42.6-46.6) | 22.5 (21.4-23.6) | 41.2 (37.4-45.0) | 21.1 (19.1-23.1) |
| Americas | 14.8 (12.6-17.0) | 15.1 (13.5-16.8) | 48.7 (38.7-58.8) | 32.9 (30.2-35.6) | 29.7 (27.3-32.1) | 24.6 (21.9-27.3) | 12.7 (9.8-15.7) |
| Eastern Mediterranean | 12.4 (10.8-13.9) | 16.6 (13.6-19.5) | 37.4 (32.0-42.7) | 46.2 (41.2-51.3) | 22.7 (19.9-25.6) | 33.3 (25.6-41.0) | 19.0 (16.9-21.2) |
| Europe | 7.1 (6.2-8.0) | 11.2 (10.3-12.1) | 49.8 (47.9-51.8) | 21.7 (20.0-23.4) | 36.1 (34.4-37.8) | 23.5 (21.9-25.1) | 14.3 (13.1-15.5) |
| South-East Asia | 7.9 (7.0-8.9) | 8.4 (6.8-10.0) | 30.9 (29.0-32.9) | 34.8 (32.5-37.2) | 31.1 (27.4-34.9) | 35.3 (32.3-38.2) | 23.1 (20.1-26.1) |
| Western Pacific | 11.2 (10.3-12.2) | 8.1 (7.5-8.8) | 48.6 (44.3-52.9) | 29.1 (28.0-30.3) | 18.0 (16.9-19.0) | 27.1 (25.5-28.8) | 12.3 (11.3-13.4) |
| *P* value | <0.001 | <0.001 | <0.001 | <0.001 | <0.001 | <0.001 | <0.001 |
| **World Bank income category** |  |  |  |  |  |  |  |
| Low income | 13.5 (11.3-15.8) | 12.3 (10.5-14.2) | 31.2 (27.1-35.3) | 40.4 (38.0-42.7) | 30.5 (26.6-34.5) | 50.5 (46.6-54.4) | 45.2 (41.7-48.7) |
| Lower-Middle income | 11.0 (9.9-12.0) | 14.0 (12.0-16.0) | 35.9 (33.2-38.7) | 38.2 (35.8-40.7) | 21.8 (20.0-23.5) | 29.8 (25.5-34.1) | 15.0 (13.5-16.4) |
| Upper-Middle income | 14.3 (12.5-16.2) | 14.4 (13.2-15.5) | 50.5 (42.8-58.2) | 35.7 (33.6-37.8) | 28.8 (27.1-30.5) | 25.7 (23.6-27.8) | 13.5 (11.5-15.6) |
| High income | 6.5 (5.6-7.4) | 11.6 (10.6-12.5) | 32.4 (30.4-34.3) | 16.9 (15.7-18.1) | 36.7 (34.8-38.7) | 23.0 (21.1-24.9) | 15.2 (13.6-16.8) |
| *P* value | <0.001 | 0.14 | <0.001 | <0.001 | <0.001 | <0.001 | <0.001 |
| **Current any tobacco use**^*^ |  |  |  |  |  |  |  |
| Yes | 17.0 (13.7-20.2) | 18.9 (17.4-20.3) | 50.0 (47.7-52.3) | 35.6 (32.8-38.3) | 36.8 (34.2-39.4) | 31.9 (28.0-35.7) | 21.9 (19.0-24.8) |
| No | 10.4 (9.1-11.6) | 12.0 (10.9-13.1) | 40.0 (33.3-46.7) | 35.8 (34.4-37.2) | 25.9 (24.4-27.4) | 28.1 (25.9-30.3) | 15.4 (13.7-17.0) |
| *P* value | <0.001 | <0.001 | 0.02 | 0.85 | <0.001 | 0.11 | <0.001 |
| **FCTC ratification** |  |  |  |  |  |  |  |
| Yes | 12.2 (11.1-13.2) | 13.8 (12.8-14.8) | 36.2 (34.1-38.4) | 37.4 (35.9-38.8) | 28.5 (27.1-30.0) | 31.0 (28.8-33.3) | 19.1 (17.9-20.4) |
| No | 12.0 (9.8-14.2) | 10.8 (9.1-12.5) | 50.7 (41.6-59.7) | 25.3 (22.9-27.8) | 18.2 (16.2-20.1) | 24.2 (20.9-27.4) | 10.4 (7.0-13.8) |
| *P* value | 0.89 | 0.01 | 0.002 | <0.001 | <0.001 | 0.001 | <0.001 |
| **Monitoring tobacco use** |  |  |  |  |  |  |  |
| Yes | 9.6 (8.4-10.7) | 10.0 (8.4-11.6) | 24.6 (21.6-27.6) | 30.7 (28.6-32.8) | 24.8 (23.1-26.6) | 28.6 (24.5-32.7) | 13.0 (11.3-14.8) |
| No | 13.5 (12.2-14.8) | 15.4 (14.3-16.5) | 48.7 (43.8-53.6) | 39.2 (37.7-40.7) | 28.9 (27.1-30.6) | 29.2 (27.4-31.1) | 18.2 (16.4-20.1) |
| *P* value | <0.001 | <0.001 | <0.001 | <0.001 | 0.002 | 0.78 | <0.001 |
| **Warning about the dangers of tobacco** |  |  |  |  |  |  |  |
| Yes | 11.6 (9.7-13.4) | 12.5 (10.9-14.1) | 26.4 (23.1-29.7) | 35.3 (33.1-37.5) | 27.7 (25.8-29.5) | 27.3 (24.1-30.6) | 13.6 (12.0-15.2) |
| No | 12.5 (11.4-13.5) | 14.2 (13.1-15.3) | 47.3 (42.2-52.4) | 36.6 (35.3-37.9) | 27.2 (25.5-29.0) | 30.4 (28.3-32.5) | 18.7 (16.6-20.8) |
| *P* value | 0.41 | 0.09 | <0.001 | 0.36 | 0.75 | 0.11 | <0.001 |
| **Enforcing tobacco advertising bans** |  |  |  |  |  |  |  |
| Yes | 11.1 (9.7-12.5) | 14.7 (13.1-16.4) | 33.2 (30.6-35.7) | 46.7 (44.8-48.7) | 17.1 (15.7-18.5) | 26.7 (25.3-28.2) | 14.4 (12.8-16.1) |
| No | 12.1 (11.2-13.1) | 13.3 (12.3-14.4) | 41.3 (36.4-46.2) | 34.7 (33.3-36.1) | 28.6 (27.2-30.1) | 29.1 (27.2-30.9) | 16.9 (15.5-18.2) |
| *P* value | 0.23 | 0.14 | 0.004 | <0.001 | <0.001 | 0.06 | 0.04 |

Abbreviation: WHO, World Health Organization.

Data are presented as %(95%CI).

^*^Current any tobacco use was defined as using either cigarettes or other tobacco products (e.g., chewing tobacco, snuff, dip, cigars, cigarillos, pipe, e-cigarettes) on at least 1 day during the past 30 days.

**Table S10 Proportions of incorrect beliefs and positive attitudes toward tobacco smoking among adolescents aged 12–16 years who are exposed to secondhand smoke, stratified by age, sex, WHO region, World Bank income category, current tobacco use status, FCTC ratification, tobacco use monitoring, tobacco warning policies, and enforcement of tobacco advertising bans.**

|  | **Tobacco smoking is not harmful** | **Secondhand smoke exposure is not harmful** | **It is safe to smoke one or two years then quit** | **Once someone smoke, it is easy to quit** | **Tobacco smoking helps people feel more comfortable** | **Tobacco smoking makes more friends** | **Tobacco smoking is more attractive** |
| --- | --- | --- | --- | --- | --- | --- | --- |
| **Total** | 16.4 (12.6-20.3) | 20.2 (19.0-21.4) | 44.2 (37.5-50.9) | 47.1 (45.7-48.5) | 23.1 (22.1-24.2) | 23.6 (20.4-26.8) | 15.2 (12.9-17.5) |
| **Sex** |  |  |  |  |  |  |  |
| Boys | 20.9 (15.9-25.9) | 22.7 (21.4-24.1) | 40.8 (34.4-47.2) | 51.0 (49.1-52.9) | 24.8 (23.5-26.1) | 24.8 (21.5-28.1) | 18.2 (15.6-20.9) |
| Girls | 12.7 (9.9-15.5) | 17.7 (16.2-19.1) | 46.9 (39.6-54.2) | 43.0 (41.2-44.9) | 21.4 (20.1-22.8) | 22.6 (18.9-26.4) | 12.6 (10.1-15.1) |
| *P* value | <0.001 | <0.001 | 0.003 | <0.001 | <0.001 | 0.19 | <0.001 |
| **Age group** |  |  |  |  |  |  |  |
| 12-14 years | 16.0 (11.1-20.9) | 19.2 (18.0-20.4) | 48.0 (40.1-56.0) | 46.6 (45.0-48.1) | 22.6 (21.5-23.8) | 22.1 (17.7-26.4) | 13.1 (10.8-15.5) |
| 15-16 years | 17.7 (15.4-20.0) | 22.3 (20.4-24.3) | 33.2 (28.6-37.9) | 48.0 (46.1-50.0) | 24.2 (22.7-25.7) | 28.3 (26.2-30.4) | 21.3 (17.5-25.1) |
| *P* value | 0.48 | 0.001 | 0.001 | 0.17 | 0.08 | 0.02 | <0.001 |
| **WHO region** |  |  |  |  |  |  |  |
| Africa | 25.6 (22.5-28.8) | 30.2 (26.6-33.7) | 10.4 (8.2-12.6) | 53.9 (51.7-56.1) | 19.8 (18.0-21.6) | 34.2 (31.5-36.9) | 20.7 (18.5-22.9) |
| Americas | 17.6 (10.4-24.7) | 22.8 (20.2-25.3) | 53.1 (45.5-60.8) | 43.6 (41.5-45.7) | 25.5 (23.8-27.2) | 20.1 (15.1-25.1) | 10.1 (6.7-13.5) |
| Eastern Mediterranean | 14.4 (11.6-17.2) | 18.8 (16.5-21.0) | 40.6 (36.1-45.0) | 60.3 (55.7-65.0) | 18.3 (16.0-20.6) | 25.0 (19.6-30.4) | 18.8 (16.0-21.6) |
| Europe | 9.5 (8.5-10.6) | 12.9 (11.5-14.3) | 51.0 (48.5-53.4) | 29.5 (27.7-31.4) | 25.1 (23.4-26.8) | 20.3 (18.4-22.3) | 16.0 (14.3-17.8) |
| South-East Asia | 10.8 (8.8-12.8) | 12.4 (10.9-14.0) | 26.7 (24.5-28.8) | 44.7 (41.4-48.0) | 28.6 (26.0-31.1) | 30.6 (27.1-34.1) | 23.9 (20.9-26.9) |
| Western Pacific | 18.2 (16.4-19.9) | 14.2 (13.1-15.4) | 41.8 (37.5-46.1) | 40.5 (38.6-42.4) | 15.8 (14.7-17.0) | 25.2 (23.3-27.1) | 14.5 (13.0-16.0) |
| *P* value | 0.02 | <0.001 | <0.001 | <0.001 | <0.001 | 0.001 | <0.001 |
| **World Bank income category** |  |  |  |  |  |  |  |
| Low income | 19.0 (16.6-21.5) | 18.1 (16.1-20.0) | 25.8 (22.2-29.3) | 46.2 (43.5-49.0) | 23.6 (21.2-26.1) | 38.0 (34.0-42.1) | 36.9 (33.7-40.0) |
| Lower-Middle income | 15.1 (13.2-17.0) | 20.9 (18.3-23.4) | 35.0 (32.1-37.9) | 53.2 (50.5-55.8) | 20.4 (18.8-22.0) | 25.4 (22.8-28.0) | 16.0 (14.2-17.9) |
| Upper-Middle income | 17.5 (10.9-24.0) | 22.1 (20.1-24.0) | 53.2 (46.2-60.2) | 44.9 (43.1-46.7) | 25.4 (24.0-26.8) | 21.1 (16.8-25.4) | 11.4 (8.5-14.3) |
| High income | 7.8 (6.7-9.0) | 13.4 (12.0-14.9) | 32.3 (29.8-34.8) | 26.8 (24.5-29.1) | 21.3 (19.3-23.4) | 21.1 (17.9-24.4) | 16.5 (13.6-19.4) |
| *P* value | 0.12 | 0.001 | <0.001 | <0.001 | <0.001 | <0.001 | <0.001 |
| **Current any tobacco use**^*^ |  |  |  |  |  |  |  |
| Yes | 18.2 (9.9-26.6) | 27.6 (24.4-30.8) | 60.1 (47.4-72.9) | 55.9 (50.3-61.6) | 32.4 (28.9-35.9) | 27.6 (21.8-33.4) | 16.2 (11.0-21.5) |
| No | 16.0 (11.9-20.1) | 19.5 (18.2-20.7) | 42.1 (34.8-49.4) | 46.3 (44.8-47.7) | 22.4 (21.4-23.5) | 22.9 (19.1-26.7) | 14.5 (12.0-17.0) |
| *P* value | 0.63 | <0.001 | 0.01 | 0.001 | <0.001 | 0.07 | 0.39 |
| **FCTC ratification** |  |  |  |  |  |  |  |
| Yes | 15.9 (14.7-17.2) | 19.4 (18.3-20.6) | 32.2 (30.1-34.2) | 47.6 (46.1-49.0) | 23.4 (22.3-24.4) | 27.3 (25.8-28.9) | 19.2 (17.9-20.5) |
| No | 17.3 (7.9-26.6) | 28.3 (22.6-34.1) | 60.4 (55.9-64.9) | 41.4 (36.5-46.4) | 20.7 (16.7-24.7) | 18.5 (12.2-24.7) | 8.6 (4.5-12.7) |
| *P* value | 0.77 | 0.001 | <0.001 | 0.02 | 0.23 | 0.02 | <0.001 |
| **Monitoring tobacco use** |  |  |  |  |  |  |  |
| Yes | 13.5 (11.0-16.1) | 12.3 (11.0-13.5) | 21.8 (16.3-27.2) | 43.6 (40.4-46.8) | 20.0 (18.4-21.7) | 23.1 (20.3-25.9) | 12.2 (9.8-14.5) |
| No | 17.1 (12.6-21.6) | 23.1 (21.6-24.7) | 48.2 (42.0-54.5) | 48.4 (46.9-50.0) | 24.3 (23.1-25.5) | 23.8 (19.7-27.8) | 15.9 (13.0-18.9) |
| *P* value | 0.16 | <0.001 | <0.001 | 0.009 | <0.001 | 0.79 | 0.05 |
| **Warning about the dangers of tobacco** |  |  |  |  |  |  |  |
| Yes | 14.1 (11.8-16.4) | 16.2 (14.8-17.6) | 20.7 (17.0-24.4) | 47.5 (45.1-49.9) | 23.0 (21.5-24.6) | 23.6 (21.5-25.7) | 12.8 (10.8-14.7) |
| No | 17.3 (12.3-22.3) | 23.0 (21.4-24.7) | 51.5 (45.9-57.1) | 46.7 (45.1-48.3) | 23.2 (21.9-24.5) | 23.6 (19.0-28.3) | 16.2 (12.8-19.5) |
| *P* value | 0.23 | <0.001 | <0.001 | 0.60 | 0.84 | 0.99 | 0.08 |
| **Enforcing tobacco advertising bans** |  |  |  |  |  |  |  |
| Yes | 17.0 (12.8-21.2) | 21.7 (19.3-24.0) | 28.9 (26.5-31.2) | 50.8 (48.1-53.5) | 14.1 (12.8-15.5) | 20.1 (18.1-22.2) | 15.8 (13.1-18.5) |
| No | 16.4 (12.5-20.3) | 20.0 (18.6-21.3) | 44.5 (37.8-51.2) | 46.5 (44.9-48.0) | 24.5 (23.4-25.6) | 23.7 (20.4-27.0) | 15.2 (12.8-17.6) |
| *P* value | 0.84 | 0.22 | <0.001 | 0.007 | <0.001 | 0.07 | 0.73 |

Abbreviation: WHO, World Health Organization.

Data are presented as %(95%CI).

^*^Current any tobacco use was defined as using either cigarettes or other tobacco products (e.g., chewing tobacco, snuff, dip, cigars, cigarillos, pipe, e-cigarettes) on at least 1 day during the past 30 days.

**Table S11 Trends in the proportions of adolescents aged 12–16 years who believe that tobacco smoking is not harmful and that secondhand smoke exposure is not harmful, from 1999 to 2019, by country/territory.**

| **Country/territory** | **Tobacco smoking is not harmful** | | | | | | |  | **Secondhand smoke exposure is not harmful** | | | | | |
| --- | --- | --- | --- | --- | --- | --- | --- | --- | --- | --- | --- | --- | --- | --- |
|  | **Representativeness** | **Survey year** | **First year, %**  **(95%CI)** | **Last year, %**  **(95%CI)** | **Total absolute change, %** | **Absolute change/5-years, %** | ***P* for trend** |  | **Survey year** | **First year, %**  **(95%CI)** | **Last year, %**  **(95%CI)** | **Total absolute change, %** | **Absolute change/5-years, %** | ***P* for trend** |
| **Africa** |  |  |  |  |  |  |  |  |  |  |  |  |  |  |
| Burkina Faso | Subnational | 2001,2006,2009 | 20.6  (14.5-26.6) | 24.1  (18.7-29.5) | 3.5 | 2.2 | 0.27 |  | 2001,2006,2009 | 23.8  (17.0-30.7) | 31.0  (27.2-34.8) | 7.2 | 4.5 | 0.05 |
| Congo | National | NA | NA | NA | NA | NA | NA |  | 2006,2009,2019 | 46.7  (39.6-53.9) | 39.0  (33.6-44.4) | -7.7 | -3.0 | 0.20 |
| Ghana | National | 2000,2006,2009 | 40.4  (33.0-47.8) | 47.5  (40.6-54.4) | 7.1 | 3.9 | 0.17 |  | 2000,2006,2009,2017 | 47.5  (37.9-57.0) | 29.3  (25.5-33.1) | -18.2 | -5.4 | <0.001 |
| Kenya | National | NA | NA | NA | NA | NA | NA |  | 2001,2007,2013 | 33.5  (27.8-39.1) | 16.5  (10.6-22.3) | -17.0 | -7.1 | <0.001 |
| Malawi | National | 2000,2005,2009 | 7.9  (5.7-10.0) | 4.9  (1.6-8.1) | -3.0 | -1.7 | 0.03 |  | 2000,2005,2009 | 10.4  (6.9-13.9) | 14.4  (11.9-16.9) | 4.0 | 2.2 | 0.06 |
| Mauritania | National | 2001,2006,2009,2018 | 30.9  (26.0-35.9) | 28.3  (23.0-33.6) | -2.6 | -0.8 | 0.006 |  | 2001,2006,2009,2018 | 38.3  (34.9-41.8) | 29.5  (24.5-34.6) | -8.8 | -2.6 | <0.001 |
| Mauritius | National | 2003,2008,2016 | 19.8  (14.5-25.1) | 13.7  (9.3-18.1) | -6.1 | -2.3 | 0.15 |  | 2003,2008,2016 | 22.4  (16.7-28.2) | 15.5  (10.5-20.5) | -6.9 | -2.7 | 0.12 |
| Mozambique | National | NA | NA | NA | NA | NA | NA |  | 2002,2007,2013 | 27.8  (25.1-30.6) | 31.0  (27.0-35.0) | 3.2 | 1.5 | 0.08 |
| Niger | National | 2001,2006,2009 | 37.2  (31.1-43.2) | 20.1  (13.9-26.3) | -17.1 | -10.7 | <0.001 |  | 2001,2006,2009 | 34.4  (27.2-41.6) | 19.6  (13.6-25.6) | -14.8 | -9.2 | 0.001 |
| Senegal | National | NA | NA | NA | NA | NA | NA |  | 2002,2007,2013 | 29.1  (25.9-32.2) | 49.5  (35.6-63.5) | 20.4 | 9.3 | 0.10 |
| Seychelles | National | NA | NA | NA | NA | NA | NA |  | 2002,2007,2015 | 26.0  (20.1-31.9) | 28.7  (24.9-32.4) | 2.7 | 1.0 | 0.32 |
| South Africa | National | 1999,2002,2008,2011 | 30.2  (23.1-37.3) | 29.6  (25.5-33.7) | -0.6 | -0.2 | 0.58 |  | 1999,2002,2008,2011 | 30.6  (23.3-37.8) | 32.1  (28.4-35.7) | 1.5 | 0.6 | 0.30 |
| Swaziland | National | 2001,2005,2009 | 14.1  (12.3-15.9) | 29.3  (22.9-35.8) | 15.2 | 9.5 | <0.001 |  | 2001,2005,2009 | 22.7  (20.5-24.8) | 27.5  (22.0-33.0) | 4.8 | 3.0 | 0.04 |
| Togo | National | 2002,2007,2013,2019 | 30.5  (23.4-37.7) | 29.1  (18.9-39.4) | -1.4 | -0.4 | 0.93 |  | 2002,2007,2013,2019 | 23.6  (18.7-28.4) | 24.1  (16.2-31.9) | 0.5 | 0.1 | 0.36 |
| Uganda | National | 2002,2007,2011 | 17.2  (13.4-20.9) | 34.1  (27.1-41.1) | 16.9 | 9.4 | <0.001 |  | 2002,2007,2011,2018 | 22.1  (18.1-26.1) | 21.3  (16.8-25.8) | -0.8 | -0.2 | 0.003 |
| United Republic of Tanzania | Subnational | 2003,2008,2016 | 5.7  (4.5-6.8) | 11.8  (9.4-14.2) | 6.1 | 2.3 | <0.001 |  | 2003,2008,2016 | 8.6  (7.2-10.1) | 17.5  (13.1-22.0) | 8.9 | 3.4 | <0.001 |
| Zambia | National | 2002,2007,2011 | 45.0  (40.0-50.0) | 37.5  (35.7-39.3) | -7.5 | -4.2 | 0.32 |  | 2002,2007,2011 | 52.2  (47.4-57.0) | 42.4  (40.6-44.3) | -9.8 | -5.4 | 0.07 |
| Zimbabwe | National | 1999,2003,2008,2014 | 29.2  (23.1-35.2) | 43.7  (37.4-50.0) | 14.5 | 4.8 | <0.001 |  | 1999,2003,2008,2014 | 36.8  (31.3-42.4) | 41.0  (33.1-48.9) | 4.2 | 1.4 | 0.22 |
| **Americas** |  |  |  |  |  |  |  |  |  |  |  |  |  |  |
| Antigua and Barbuda | National | 2000,2004,2009,2017 | 11.9  (8.4-15.4) | 14.2  (11.8-16.6) | 2.3 | 0.7 | 0.95 |  | 2000,2004,2009,2017 | 18.4  (14.4-22.3) | 14.1  (11.8-16.4) | -4.3 | -1.3 | 0.008 |
| Argentina | National | 2000,2007,2018 | 2.0  (1.3-2.7) | 21.9  (13.7-30.2) | 19.9 | 5.5 | <0.001 |  | 2000,2007,2012,2018 | 7.7  (6.3-9.2) | 16.2  (11.4-21.0) | 8.5 | 2.4 | <0.001 |
| Bahamas | National | 2000,2004,2009,2013 | 10.4  (7.6-13.3) | 14.3  (8.5-20.1) | 3.9 | 1.5 | 0.14 |  | 2000,2004,2009,2013 | 14.6  (12.1-17.1) | 17.3  (12.5-22.1) | 2.7 | 1.0 | 0.22 |
| Barbados | National | 1999,2002,2007 | 3.6  (2.8-4.3) | 10.4  (8.1-12.7) | 6.8 | 4.2 | <0.001 |  | 1999,2002,2007,2013 | 11.5  (8.8-14.1) | 17.3  (14.5-20.1) | 5.8 | 2.1 | 0.02 |
| Belize | National | NA | NA | NA | NA | NA | NA |  | 2002,2008,2014 | 17.8  (12.7-22.9) | 15.9  (13.3-18.5) | -1.9 | -0.8 | 0.86 |
| Bolivia | Subnational | 2000,2003,2012,2018 | 6.0  (5.3-6.6) | 26.9  (23.4-30.3) | 20.9 | 5.8 | <0.001 |  | 2000,2003,2012,2018 | 9.2  (8.4-9.9) | 19.8  (17.3-22.3) | 10.6 | 2.9 | <0.001 |
| Brazil | Subnational | 2002,2005,2006,2007,2012 | 4.8  (4.0-5.6) | 7.8  (6.3-9.3) | 3.0 | 1.5 | <0.001 |  | 2002,2005,2006,2007,2012 | 9.0  (8.0-10.1) | 10.0  (8.4-11.6) | 1.0 | 0.5 | 0.27 |
| Chile | Subnational | 2000,2003,2008 | 4.6  (4.0-5.2) | 3.7  (3.2-4.1) | -0.9 | -0.6 | 0.03 |  | 2000,2003,2008,2016 | 7.8  (7.0-8.7) | 11.4  (9.9-13.0) | 3.6 | 1.1 | 0.007 |
| Costa Rica | National | 1999,2002,2008,2013 | 3.5  (2.8-4.1) | 18.9  (16.1-21.8) | 15.4 | 5.5 | <0.001 |  | 1999,2002,2008,2013 | 5.9  (5.0-6.7) | 15.0  (12.7-17.2) | 9.1 | 3.2 | <0.001 |
| Cuba | National | 2000,2004,2010,2018 | 5.6  (4.6-6.6) | 17.4  (14.2-20.6) | 11.8 | 3.3 | <0.001 |  | 2000,2004,2010,2018 | 10.2  (8.9-11.6) | 10.9  (9.3-12.6) | 0.7 | 0.2 | 0.04 |
| Dominica | National | 2000,2004,2009 | 16.8  (13.5-20.1) | 15.4  (11.7-19.0) | -1.4 | -0.8 | 0.55 |  | 2000,2004,2009 | 17.3  (14.5-20.2) | 16.1  (12.4-19.8) | -1.2 | -0.7 | 0.42 |
| Dominican Republic | National | NA | NA | NA | NA | NA | NA |  | 2004,2011,2016 | 10.9  (9.1-12.7) | 36.7  (30.1-43.2) | 25.8 | 10.8 | <0.001 |
| Ecuador | Subnational | NA | NA | NA | NA | NA | NA |  | 2001,2007,2016 | 10.3  (8.5-12.2) | 32.5  (28.4-36.7) | 22.2 | 7.4 | <0.001 |
| El Salvador | National | NA | NA | NA | NA | NA | NA |  | 2003,2009,2015 | 5.8  (3.9-7.8) | 6.9  (5.5-8.2) | 1.1 | 0.5 | 0.45 |
| Grenada | National | 2000,2004,2009 | 17.3  (14.6-20.0) | 14.4  (11.7-17.0) | -2.9 | -1.6 | 0.04 |  | 2000,2004,2009,2016 | 19.5  (16.2-22.8) | 12.4  (10.0-14.7) | -7.1 | -2.2 | 0.002 |
| Guatemala | National | 2002,2008,2015 | 3.3  (2.5-4.1) | 31.0  (27.7-34.4) | 27.7 | 10.7 | <0.001 |  | 2002,2008,2015 | 5.5  (4.2-6.8) | 13.0  (11.1-14.9) | 7.5 | 2.9 | 0.03 |
| Guyana | National | 2000,2004,2010 | 11.2  (7.3-15.1) | 15.0  (11.2-18.8) | 3.8 | 1.9 | 0.16 |  | 2000,2004,2010,2015 | 18.2  (12.3-24.0) | 26.8  (19.9-33.7) | 8.6 | 2.9 | 0.26 |
| Jamaica | National | 2000,2006,2010 | 15.7  (10.8-20.5) | 13.6  (9.6-17.5) | -2.1 | -1.0 | 0.87 |  | 2000,2006,2010,2017 | 17.3  (12.7-22.0) | 15.1  (13.2-17.0) | -2.2 | -0.6 | 0.48 |
| Mexico | Subnational | 2003,2005,2006,2008,2011 | 4.5  (3.8-5.2) | 6.2  (4.3-8.1) | 1.7 | 1.1 | 0.10 |  | 2003,2005,2006,2008,2011 | 7.6  (6.8-8.3) | 7.4  (5.7-9.1) | -0.2 | -0.1 | 0.95 |
| Nicaragua | National | NA | NA | NA | NA | NA | NA |  | 2003,2014,2019 | 9.8  (8.0-11.5) | 12.3  (10.7-13.9) | 2.5 | 0.8 | 0.06 |
| Panama | National | 2002,2008,2012 | 7.3  (5.6-8.9) | 14.1  (12.0-16.2) | 6.8 | 3.4 | 0.001 |  | 2002,2008,2012,2017 | 8.5  (6.9-10.1) | 9.2  (7.5-10.9) | 0.7 | 0.2 | 0.18 |
| Paraguay | National | 2003,2008,2014,2019 | 7.7  (6.9-8.5) | 38.5  (33.5-43.5) | 30.8 | 9.6 | <0.001 |  | 2003,2008,2014,2019 | 10.4  (9.4-11.4) | 30.1  (26.3-33.9) | 19.7 | 6.2 | <0.001 |
| Peru | National | 2000,2002,2003,2007 | 4.1  (3.0-5.2) | 5.9  (5.0-6.9) | 1.8 | 1.3 | 0.03 |  | 2000,2002,2003,2007,2014,2019 | 9.8  (8.4-11.2) | 9.7  (7.9-11.6) | -0.1 | -0.0 | 0.02 |
| Saint Lucia | National | 2000,2007,2011,2017 | 10.8  (8.0-13.7) | 17.8  (14.9-20.8) | 7.0 | 2.1 | 0.001 |  | 2000,2007,2011 | 12.6  (9.1-16.0) | 16.2  (13.3-19.1) | 3.6 | 1.6 | 0.14 |
| Saint Vincent and the Grenadines | National | 2000,2007,2011,2018 | 12.5  (9.5-15.6) | 16.2  (12.9-19.6) | 3.7 | 1.0 | 0.25 |  | 2000,2007,2011,2018 | 15.4  (12.3-18.4) | 16.4  (13.7-19.0) | 1.0 | 0.3 | 0.72 |
| Suriname | National | 2000,2004,2009,2016 | 3.7  (2.4-5.1) | 9.4  (7.6-11.2) | 5.7 | 1.8 | <0.001 |  | 2000,2004,2009,2016 | 15.6  (12.5-18.7) | 9.4  (7.6-11.2) | -6.2 | -1.9 | 0.003 |
| Trinidad and Tobago | National | 2000,2007,2011,2017 | 5.0  (3.9-6.1) | 14.3  (11.4-17.3) | 9.3 | 2.7 | <0.001 |  | 2000,2007,2011,2017 | 9.7  (8.0-11.3) | 20.2  (17.4-23.1) | 10.5 | 3.1 | <0.001 |
| Uruguay | National | NA | NA | NA | NA | NA | NA |  | 2000,2007,2014 | 9.0  (7.5-10.6) | 9.7  (8.4-11.0) | 0.7 | 0.2 | 0.009 |
| Venezuela | National | 1999,2001,2003,2008 | 7.8  (6.6-9.0) | 4.8  (2.9-6.8) | -3.0 | -1.7 | 0.63 |  | 1999,2001,2003,2008,2019 | 9.0  (8.1-10.0) | 31.1  (27.2-34.9) | 22.1 | 5.5 | <0.001 |
| **Eastern Mediterranean** | |  |  |  |  |  |  |  |  |  |  |  |  |  |
| Afghanistan | Subnational | NA | NA | NA | NA | NA | NA |  | 2004,2010,2017 | 5.8  (4.5-7.1) | 22.3  (17.9-26.6) | 16.5 | 6.3 | 0.72 |
| Djibouti | National | NA | NA | NA | NA | NA | NA |  | 2003,2009,2013 | 44.0  (39.4-48.6) | 51.2  (44.8-57.6) | 7.2 | 3.6 | 0.05 |
| Egypt | National | 2001,2005,2009,2014 | 10.0  (7.0-13.1) | 14.3  (10.4-18.3) | 4.3 | 1.7 | 0.03 |  | 2001,2005,2009,2014 | 13.3  (9.7-16.9) | 16.2  (9.2-23.3) | 2.9 | 1.1 | 0.46 |
| Gaza Strip | Subnational | NA | NA | NA | NA | NA | NA |  | 2000,2005,2013,2019 | 8.1  (4.5-11.8) | 20.1  (13.7-26.6) | 12.0 | 3.2 | 0.002 |
| Iraq | National | 2006,2008,2014 | 11.0  (8.4-13.6) | 18.9  (13.4-24.3) | 7.9 | 4.9 | 0.008 |  | 2006,2008,2014,2019 | 16.1  (11.8-20.4) | 24.6  (21.4-27.8) | 8.5 | 3.3 | 0.05 |
| Jordan | National | 1999,2003,2007,2009,2014 | 10.6  (9.3-12.0) | 15.2  (11.9-18.6) | 4.6 | 1.5 | 0.01 |  | 1999,2003,2007,2009,2014 | 13.9  (12.2-15.6) | 16.1  (11.9-20.4) | 2.2 | 0.7 | 0.26 |
| Kuwait | National | 2001,2005,2009,2016 | 7.0  (6.0-7.9) | 12.9  (10.0-15.7) | 5.9 | 2.0 | <0.001 |  | 2001,2005,2009,2016 | 12.2  (10.5-13.9) | 14.1  (11.9-16.2) | 1.9 | 0.6 | 0.10 |
| Lebanon | National | 2001,2005,2011 | 4.6  (3.8-5.5) | 5.4  (4.0-6.8) | 0.8 | 0.4 | 0.67 |  | 2001,2005,2011 | 7.4  (6.4-8.4) | 5.5  (4.0-7.1) | -1.9 | -1.0 | 0.01 |
| Libyan Arab Jamahiriya | National | 2003,2007,2010 | 15.9  (12.9-18.9) | 8.0  (6.3-9.7) | -7.9 | -5.6 | <0.001 |  | 2003,2007,2010 | 19.0  (16.2-21.9) | 18.6  (15.7-21.6) | -0.4 | -0.3 | 0.87 |
| Morocco | National | 2001,2006,2010,2016 | 6.8  (5.4-8.1) | 11.6  (9.5-13.7) | 4.8 | 1.6 | 0.001 |  | 2001,2006,2010,2016 | 8.2  (6.9-9.4) | 15.1  (12.7-17.5) | 6.9 | 2.3 | <0.001 |
| Oman | National | NA | NA | NA | NA | NA | NA |  | 2002,2010,2016 | 14.2  (11.3-17.0) | 15.0  (9.0-21.0) | 0.8 | 0.3 | 0.72 |
| Pakistan | National | 2004,2008,2013 | 9.8  (7.1-12.6) | 22.6  (17.3-27.9) | 12.8 | 7.1 | <0.001 |  | 2004,2008,2013 | 22.3  (18.5-26.0) | 12.7  (9.6-15.8) | -9.6 | -5.3 | <0.001 |
| Qatar | National | NA | NA | NA | NA | NA | NA |  | 2004,2007,2013,2018 | 12.5  (9.9-15.0) | 23.5  (20.3-26.8) | 11.0 | 3.9 | <0.001 |
| Saudi Arabia | National | 2001,2007,2010 | 9.3  (7.1-11.6) | 7.2  (5.2-9.1) | -2.1 | -1.2 | 0.85 |  | 2001,2007,2010 | 10.5  (8.1-13.0) | 15.3  (12.5-18.2) | 4.8 | 2.7 | 0.001 |
| Sudan | National | 2001,2005,2009 | 10.9  (8.7-13.2) | 11.0  (7.7-14.3) | 0.1 | 0.1 | 0.91 |  | 2001,2005,2009 | 15.7  (12.8-18.6) | 13.9  (8.7-19.1) | -1.8 | -1.1 | 0.28 |
| Syrian Arab Republic | National | 2002,2007,2010 | 7.7  (5.0-10.5) | 10.5  (7.8-13.2) | 2.8 | 1.8 | 0.08 |  | 2002,2007,2010 | 8.8  (6.9-10.6) | 19.0  (13.5-24.6) | 10.2 | 6.4 | 0.001 |
| Tunisia | National | 2001,2007,2010 | 5.7  (4.8-6.6) | 5.7  (3.9-7.4) | 0.0 | 0.0 | 0.85 |  | 2001,2007,2010,2017 | 9.2  (7.9-10.6) | 12.5  (10.2-14.8) | 3.3 | 1.0 | 0.01 |
| United Arab Emirates | National | NA | NA | NA | NA | NA | NA |  | 2002,2005,2013 | 14.8  (12.6-17.0) | 14.1  (11.9-16.3) | -0.7 | -0.3 | 0.73 |
| West BANK | regional | 2000,2005,2009 | 5.4  (4.4-6.3) | 8.7  (6.2-11.3) | 3.3 | 1.8 | 0.06 |  | 2000,2005,2009,2016 | 7.1  (5.9-8.3) | 17.4  (14.5-20.4) | 10.3 | 3.2 | 0.001 |
| Yemen | National | NA | NA | NA | NA | NA | NA |  | 2003,2008,2014 | 12.3  (11.0-13.6) | 18.3  (13.0-23.6) | 6.0 | 2.7 | 0.90 |
| **Europe** |  |  |  |  |  |  |  |  |  |  |  |  |  |  |
| Albania | National | 2004,2009,2015 | 10.4  (8.7-12.1) | 15.8  (13.5-18.1) | 5.4 | 2.5 | <0.001 |  | 2004,2009,2015 | 11.6  (10.2-12.9) | 10.8  (9.0-12.7) | -0.8 | -0.4 | 0.63 |
| Bosnia and Herzegovina | National | 2003,2008,2013,2019 | 5.4  (4.5-6.3) | 7.9  (6.4-9.4) | 2.5 | 0.8 | 0.04 |  | 2003,2008,2013,2019 | 11.1  (9.8-12.4) | 10.6  (9.3-12.0) | -0.5 | -0.2 | <0.001 |
| Bulgaria | National | NA | NA | NA | NA | NA | NA |  | 2002,2008,2015 | 9.8  (8.0-11.6) | 14.8  (11.4-18.2) | 5.0 | 1.9 | 0.009 |
| Croatia (Hrvatska) | National | 2003,2007,2011,2016 | 4.0  (3.5-4.5) | 7.1  (5.4-8.7) | 3.1 | 1.2 | <0.001 |  | 2003,2007,2011,2016 | 10.5  (9.3-11.6) | 9.2  (7.1-11.3) | -1.3 | -0.5 | 0.08 |
| Czech Republic | National | 2002,2007,2011,2016 | 3.7  (2.9-4.4) | 7.0  (6.0-8.0) | 3.3 | 1.2 | <0.001 |  | 2002,2007,2011,2016 | 12.9  (11.4-14.4) | 10.0  (8.7-11.4) | -2.9 | -1.0 | 0.003 |
| Georgia | National | 2003,2008,2014,2017 | 8.1  (6.0-10.2) | 11.4  (8.8-13.9) | 3.3 | 1.2 | 0.01 |  | 2003,2008,2014,2017 | 37.4  (35.0-39.8) | 9.3  (6.8-11.7) | -28.1 | -10.0 | <0.001 |
| Italy | National | 2010,2014,2018 | 3.1  (2.1-4.1) | 3.6  (2.3-4.9) | 0.5 | 0.3 | 0.63 |  | 2010,2014,2018 | 7.6  (5.9-9.3) | 9.8  (8.2-11.4) | 2.2 | 1.4 | 0.17 |
| Kazakhstan | National | 2004,2009,2014 | 13.0  (11.5-14.4) | 8.5  (5.0-12.1) | -4.5 | -2.2 | 0.01 |  | 2004,2009,2014 | 14.1  (12.7-15.6) | 8.8  (6.4-11.3) | -5.3 | -2.6 | 0.003 |
| Kyrgyzstan | National | NA | NA | NA | NA | NA | NA |  | 2004,2008,2014,2019 | 17.3  (14.7-19.8) | 12.6  (10.8-14.3) | -4.7 | -1.6 | <0.001 |
| Latvia | National | 2002,2007,2011,2014,2019 | 3.3  (2.6-4.0) | 7.6  (6.0-9.2) | 4.3 | 1.3 | <0.001 |  | 2002,2007,2011,2014,2019 | 9.4  (7.7-11.2) | 9.7  (8.0-11.5) | 0.3 | 0.1 | 0.96 |
| Lithuania | National | 2001,2005,2009,2014,2018 | 4.8  (3.8-5.9) | 13.6  (11.5-15.8) | 8.8 | 2.6 | <0.001 |  | 2001,2005,2009,2014,2018 | 11.0  (9.5-12.6) | 17.3  (15.1-19.5) | 6.3 | 1.9 | <0.001 |
| Macedonia | National | 2003,2008,2016 | 7.2  (5.1-9.3) | 12.9  (10.5-15.2) | 5.7 | 2.2 | <0.001 |  | 2003,2008,2016 | 12.4  (10.6-14.2) | 11.0  (9.1-12.9) | -1.4 | -0.5 | 0.41 |
| Montenegro | National | 2004,2008,2014,2018 | 5.5  (4.0-6.9) | 9.2  (7.1-11.3) | 3.7 | 1.3 | 0.04 |  | 2004,2008,2014,2018 | 26.2  (23.0-29.4) | 11.7  (10.3-13.0) | -14.5 | -5.2 | <0.001 |
| Poland | National | 1999,2003,2009,2016 | 9.0  (8.0-10.0) | 16.1  (13.8-18.3) | 7.1 | 2.1 | <0.001 |  | 1999,2003,2009,2016 | 13.8  (12.6-15.1) | 18.7  (16.9-20.5) | 4.9 | 1.4 | <0.001 |
| Republic of Moldova | National | NA | NA | NA | NA | NA | NA |  | 2004,2008,2013,2019 | 15.7  (13.6-17.8) | 15.0  (13.3-16.8) | -0.7 | -0.2 | 0.39 |
| Romania | National | 2004,2009,2013,2017 | 5.8  (4.3-7.2) | 11.9  (9.5-14.3) | 6.1 | 2.3 | 0.02 |  | 2004,2009,2013,2017 | 13.6  (11.2-16.0) | 17.9  (15.5-20.2) | 4.3 | 1.7 | 0.34 |
| Russian Federation | Subnational | 1999,2002,2004 | 7.0  (6.1-7.9) | 9.0  (8.0-9.9) | 2.0 | 2.0 | 0.04 |  | 1999,2002,2004,2015 | 14.6  (12.9-16.3) | 13.3  (11.7-14.8) | -1.3 | -0.4 | 0.10 |
| San Marino | National | 2010,2014,2018 | 1.2  (0.6-1.9) | 5.5  (2.0-9.0) | 4.3 | 2.7 | 0.01 |  | 2010,2014,2018 | 5.5  (4.0-7.1) | 13.1  (10.3-16.0) | 7.6 | 4.8 | <0.001 |
| Serbia | National | 2008,2013,2017 | 5.6  (4.4-6.8) | 8.0  (7.1-8.8) | 2.4 | 1.3 | 0.05 |  | 2008,2013,2017 | 11.1  (9.3-12.8) | 9.4  (8.4-10.3) | -1.7 | -0.9 | 0.001 |
| Slovakia | National | 2003,2007,2011 | 6.1  (5.1-7.1) | 6.8  (5.6-8.1) | 0.7 | 0.4 | <0.001 |  | 2003,2007,2011,2016 | 11.3  (9.7-12.9) | 10.4  (9.0-11.8) | -0.9 | -0.3 | 0.41 |
| Slovenia | National | 2003,2007,2011 | 4.9  (4.1-5.6) | 4.8  (2.4-7.2) | -0.1 | -0.1 | 0.89 |  | 2003,2007,2011,2017 | 14.8  (12.8-16.8) | 7.2  (5.1-9.2) | -7.6 | -2.7 | <0.001 |
| Tajikistan | National | 2004,2014,2019 | 13.4  (9.7-17.0) | 9.4  (7.3-11.5) | -4.0 | -1.3 | <0.001 |  | 2004,2014,2019 | 16.3  (11.7-21.0) | 17.6  (10.9-24.4) | 1.3 | 0.4 | 0.36 |
| Turkey | National | 2003,2009,2012,2017 | 4.7  (4.1-5.2) | 9.7  (8.9-10.5) | 5.0 | 1.8 | <0.001 |  | 2003,2009,2012,2017 | 7.5  (6.9-8.1) | 8.5  (8.0-9.0) | 1.0 | 0.4 | <0.001 |
| Ukraine | National | 1999,2005,2011,2017 | 5.5  (3.8-7.3) | 10.8  (7.7-13.8) | 5.3 | 1.5 | 0.51 |  | 1999,2005,2011,2017 | 15.3  (13.9-16.6) | 15.2  (11.6-18.8) | -0.1 | -0.0 | 0.69 |
| South-East Asia |  |  |  |  |  |  |  |  |  |  |  |  |  |  |
| Bangladesh | National | NA | NA | NA | NA | NA | NA |  | 2004,2007,2013 | 4.8  (3.6-6.0) | 9.2  (5.1-13.4) | 4.4 | 2.4 | 0.55 |
| Bhutan | National | 2004,2006,2009 | 13.3  (10.5-16.1) | 12.1  (8.8-15.4) | -1.2 | -1.2 | 0.21 |  | 2004,2006,2009,2013,2019 | 20.3  (17.4-23.1) | 10.6  (8.7-12.4) | -9.7 | -3.2 | <0.001 |
| India | National | 2000,2002,2003,2004,2006,2009 | 12.8  (11.9-13.7) | 17.0  (14.9-19.1) | 4.2 | 2.3 | 0.29 |  | 2000,2002,2003,2004,2006,2009 | 15.7  (14.2-17.3) | 21.2  (18.8-23.5) | 5.5 | 3.1 | 0.02 |
| Indonesia | National | 2000,2004,2005,2006,2009,2014,2019 | 2.5  (1.8-3.1) | 7.5  (6.4-8.7) | 5.0 | 1.3 | <0.001 |  | 2000,2004,2005,2006,2009,2014,2019 | 7.6  (6.1-9.2) | 5.7  (4.6-6.9) | -1.9 | -0.5 | 0.003 |
| Maldives | National | 2004,2007,2011 | 33.1  (29.4-36.8) | 5.2  (3.9-6.5) | -27.9 | -19.9 | <0.001 |  | 2004,2007,2011,2019 | 30.2  (26.9-33.6) | 14.5  (12.5-16.4) | -15.7 | -5.2 | 0.77 |
| Myanmar | National | 2001,2004,2007,2011,2016 | 4.2  (3.2-5.3) | 6.6  (5.2-7.9) | 2.4 | 0.8 | 0.12 |  | 2001,2004,2007,2011,2016 | 10.0  (8.3-11.6) | 4.7  (3.5-5.9) | -5.3 | -1.8 | <0.001 |
| Nepal | National | 2001,2004,2007,2011 | 12.6  (9.5-15.7) | 10.6  (7.7-13.6) | -2.0 | -1.0 | 0.04 |  | 2001,2004,2007,2011 | 10.1  (7.4-12.7) | 17.9  (14.8-21.1) | 7.8 | 3.9 | 0.001 |
| Sri Lanka | National | 1999,2003,2007,2011,2015 | 11.3  (9.3-13.3) | 6.6  (4.6-8.6) | -4.7 | -1.5 | <0.001 |  | 1999,2003,2007,2011,2015 | 9.9  (8.2-11.5) | 6.5  (5.0-8.0) | -3.4 | -1.1 | <0.001 |
| Thailand | National | NA | NA | NA | NA | NA | NA |  | 2005,2009,2015 | 13.7  (11.6-15.8) | 9.4  (7.4-11.4) | -4.3 | -2.1 | 0.002 |
| Timor-Leste | National | 2006,2009,2013 | 50.7  (44.8-56.6) | 67.8  (60.0-75.6) | 17.1 | 12.2 | <0.001 |  | 2006,2009,2013,2019 | 47.5  (43.8-51.1) | 35.6  (32.0-39.2) | -11.9 | -4.6 | 0.001 |
| **Western Pacific** | |  |  |  |  |  |  |  |  |  |  |  |  |  |
| Cambodia | National | 2003,2010,2016 | 5.0  (3.2-6.9) | 6.9  (5.2-8.7) | 1.9 | 0.7 | 0.82 |  | 2003,2010,2016 | 4.8  (2.6-7.0) | 6.2  (5.2-7.3) | 1.4 | 0.5 | 0.40 |
| Cook Islands | National | 2003,2008,2016 | 23.2  (17.7-28.6) | 21.0  (17.3-24.6) | -2.2 | -0.8 | 0.50 |  | 2003,2008,2016 | 25.7  (20.2-31.2) | 26.1  (22.2-30.1) | 0.4 | 0.2 | 0.53 |
| Fiji | National | 1999,2005,2009,2016 | 18.7  (14.8-22.5) | 21.8  (16.9-26.7) | 3.1 | 0.9 | 0.32 |  | 1999,2005,2009,2016 | 19.7  (15.1-24.2) | 19.2  (15.8-22.5) | -0.5 | -0.1 | 0.25 |
| Laos | National | 2003,2007,2011,2016 | 10.1  (8.8-11.5) | 12.2  (10.1-14.2) | 2.1 | 0.8 | 0.45 |  | 2003,2007,2011,2016 | 13.1  (11.6-14.5) | 14.5  (11.3-17.8) | 1.4 | 0.5 | 0.43 |
| Macau (China) | National | 2001,2005,2010,2015 | 1.7  (0.9-2.5) | 3.8  (2.6-5.0) | 2.1 | 0.7 | <0.001 |  | 2001,2005,2010,2015 | 5.4  (3.9-7.0) | 4.3  (3.2-5.4) | -1.1 | -0.4 | 0.96 |
| Mongolia | National | 2003,2007,2014,2019 | 6.6  (5.6-7.7) | 8.1  (6.6-9.5) | 1.5 | 0.5 | 0.001 |  | 2003,2007,2014,2019 | 5.9  (4.6-7.2) | 11.6  (9.9-13.3) | 5.7 | 1.8 | <0.001 |
| Micronesia | National | 2007,2013,2019 | 40.3  (34.1-46.5) | 40.6  (38.1-43.1) | 0.3 | 0.1 | 0.64 |  | 2007,2013,2019 | 41.8  (35.9-47.7) | 38.0  (35.6-40.4) | -3.8 | -1.6 | 0.20 |
| New Zealand | National | 2007,2008,2010 | 7.2  (1.9-12.6) | 7.2  (4.5-9.9) | 0.0 | 0.0 | 0.88 |  | 2007,2008,2010 | 6.5  (1.9-11.1) | 8.7  (6.2-11.1) | 2.2 | 3.7 | 0.23 |
| Philippines | National | 2000,2004,2007,2011,2019 | 24.7  (21.3-28.0) | 19.6  (17.6-21.6) | -5.1 | -1.3 | 0.76 |  | 2000,2004,2007,2011,2015,2019 | 29.0  (25.2-32.7) | 16.3  (14.5-18.0) | -12.7 | -3.3 | 0.001 |
| South Korea | National | NA | NA | NA | NA | NA | NA |  | 2005,2008,2013 | 2.8  (2.3-3.2) | 3.4  (2.9-3.9) | 0.6 | 0.4 | 0.09 |

Abbreviation: NA, not available.

**Table S12 Trends in the proportions of adolescents aged 12–16 years who believe that it is safe to smoke for one or two years and then quit, and that quitting is easy once someone starts smoking, from 1999 to 2019, by country/territory.**

| **Country/territory** | **It is safe to smoke one or two years then quit** | | | | | | |  | **Once someone smoke, it is easy to quit** | | | | | |
| --- | --- | --- | --- | --- | --- | --- | --- | --- | --- | --- | --- | --- | --- | --- |
|  | **Representativeness** | **Survey year** | **First year, %**  **(95%CI)** | **Last year, %**  **(95%CI)** | **Total absolute change, %** | **Absolute change/5-years, %** | ***P* for trend** |  | **Survey year** | **First year, %**  **(95%CI)** | **Last year, %**  **(95%CI)** | **Total absolute change, %** | **Absolute change/5-years, %** | ***P* for trend** |
| **Africa** |  |  |  |  |  |  |  |  |  |  |  |  |  |  |
| Burkina Faso | Subnational | 2001,2006,2009 | 47.8 (42.6-53.0) | 44.2 (40.4-48.1) | -3.6 | -2.2 | 0.24 |  | 2001,2006,2009 | 59.0 (52.7-65.2) | 54.7 (50.1-59.3) | -4.3 | -2.7 | 0.39 |
| Congo | National | NA | NA | NA | NA | NA | NA |  | 2006,2009,2019 | 69.2 (63.0-75.4) | 66.4 (62.0-70.9) | -2.8 | -1.1 | 0.22 |
| Ghana | National | 2000,2006,2009 | 14.1 (10.8-17.4) | 13.7 (11.8-15.6) | -0.4 | -0.2 | 0.83 |  | 2000,2006,2009,2017 | 61.0 (54.4-67.7) | 50.6 (46.8-54.5) | -10.4 | -3.1 | 0.007 |
| Kenya | National | NA | NA | NA | NA | NA | NA |  | 2001,2007,2013 | 60.4 (55.1-65.7) | 52.0 (47.6-56.4) | -8.4 | -3.5 | 0.01 |
| Malawi | National | 2000,2005,2009 | 10.1 (8.5-11.7) | 7.2 (5.4-9.1) | -2.9 | -1.6 | <0.001 |  | 2000,2005,2009 | 59.1 (54.9-63.4) | 80.4 (76.9-84.0) | 21.3 | 11.8 | <0.001 |
| Mauritania | National | 2001,2006,2009,2018 | 47.9 (44.0-51.8) | 46.1 (40.5-51.7) | -1.8 | -0.5 | 0.22 |  | 2001,2006,2009,2018 | 62.3 (57.8-66.8) | 67.8 (64.2-71.4) | 5.5 | 1.6 | 0.03 |
| Mauritius | National | 2003,2008,2016 | 18.7 (16.2-21.1) | 17.5 (15.6-19.5) | -1.2 | -0.5 | 0.50 |  | 2003,2008,2016 | 35.3 (30.6-40.1) | 28.3 (24.5-32.1) | -7.0 | -2.7 | 0.01 |
| Mozambique | National | NA | NA | NA | NA | NA | NA |  | 2002,2007,2013 | 35.4 (30.7-40.1) | 47.0 (43.3-50.6) | 11.6 | 5.3 | <0.001 |
| Niger | National | 2001,2006,2009 | 54.4 (49.9-59.0) | 41.5 (32.2-50.8) | -12.9 | -8.1 | 0.01 |  | 2001,2006,2009 | 64.2 (60.7-67.7) | 55.5 (47.6-63.4) | -8.7 | -5.4 | 0.04 |
| Senegal | National | NA | NA | NA | NA | NA | NA |  | 2002,2007,2013 | 48.8 (45.7-52.0) | 44.1 (35.5-52.7) | -4.7 | -2.1 | 0.001 |
| Seychelles | National | NA | NA | NA | NA | NA | NA |  | 2002,2007,2015 | 45.0 (40.1-49.9) | 38.5 (34.9-42.1) | -6.5 | -2.5 | 0.01 |
| South Africa | National | 1999,2002,2008,2011 | 20.2 (15.4-25.0) | 13.3 (12.0-14.5) | -6.9 | -2.9 | 0.005 |  | 1999,2002,2008,2011 | 53.2 (45.5-61.0) | 50.2 (45.5-54.8) | -3.0 | -1.2 | 0.41 |
| Swaziland | National | 2001,2005,2009 | 9.9 (8.9-10.9) | 23.7 (17.7-29.7) | 13.8 | 8.6 | <0.001 |  | 2001,2005,2009 | 49.2 (47.0-51.4) | 44.0 (39.8-48.3) | -5.2 | -3.3 | 0.07 |
| Togo | National | NA | NA | NA | NA | NA | NA |  | 2002,2007,2013,2019 | 56.4 (52.4-60.5) | 47.9 (38.4-57.4) | -8.5 | -2.5 | 0.08 |
| Uganda | National | 2002,2007,2011 | 12.4 (10.5-14.3) | 9.8 (5.1-14.5) | -2.6 | -1.4 | 0.13 |  | 2002,2007,2011,2018 | 46.6 (44.0-49.3) | 53.6 (48.4-58.8) | 7.0 | 2.2 | 0.90 |
| United Republic of Tanzania | Subnational | NA | NA | NA | NA | NA | NA |  | 2003,2008,2016 | 83.8 (82.3-85.2) | 44.2 (40.9-47.5) | -39.6 | -15.2 | <0.001 |
| Zambia | National | 2002,2007,2011 | 15.1 (13.7-16.4) | 18.0 (16.6-19.4) | 2.9 | 1.6 | 0.65 |  | 2002,2007,2011 | 53.2 (49.5-57.0) | 41.2 (39.4-43.0) | -12.0 | -6.7 | 0.001 |
| Zimbabwe | National | 1999,2003,2008 | 13.6 (11.5-15.7) | 33.4 (29.6-37.1) | 19.8 | 11.0 | <0.001 |  | 1999,2003,2008,2014 | 51.7 (47.8-55.6) | 53.8 (48.8-58.9) | 2.1 | 0.7 | 0.20 |
| **Americas** |  |  |  |  |  |  |  |  |  |  |  |  |  |  |
| Antigua and Barbuda | National | 2000,2004,2009,2017 | 10.4 (9.0-11.7) | 13.0 (11.3-14.6) | 2.6 | 0.8 | 0.02 |  | 2000,2004,2009,2017 | 33.1 (29.7-36.5) | 29.2 (26.6-31.9) | -3.9 | -1.1 | <0.001 |
| Argentina | National | NA | NA | NA | NA | NA | NA |  | 2000,2007,2012,2018 | 26.8 (23.5-30.1) | 31.8 (24.5-39.1) | 5.0 | 1.4 | 0.44 |
| Bahamas | National | 2000,2004,2009 | 12.0 (10.0-13.9) | 10.9 (9.3-12.5) | -1.1 | -0.6 | 0.51 |  | 2000,2004,2009,2013 | 33.9 (31.4-36.4) | 42.5 (28.9-56.1) | 8.6 | 3.3 | 0.13 |
| Barbados | National | 1999,2002,2007 | 13.5 (10.6-16.5) | 17.5 (15.0-19.9) | 4.0 | 2.5 | 0.12 |  | 1999,2002,2007,2013 | 26.2 (21.4-31.0) | 30.5 (27.7-33.4) | 4.3 | 1.5 | 0.66 |
| Belize | National | NA | NA | NA | NA | NA | NA |  | 2002,2008,2014 | 36.6 (33.7-39.5) | 31.1 (28.3-33.9) | -5.5 | -2.3 | 0.01 |
| Bolivia | Subnational | 2000,2003,2012,2018 | 32.2 (30.8-33.6) | 19.4 (18.0-20.7) | -12.8 | -3.6 | <0.001 |  | 2000,2003,2012,2018 | 45.4 (43.7-47.1) | 45.5 (41.6-49.4) | 0.1 | 0.0 | 0.93 |
| Brazil | Subnational | 2002,2005,2006,2007,2012 | 11.1 (10.3-12.0) | 9.7 (8.3-11.0) | -1.4 | -0.7 | 0.36 |  | 2002,2005,2006,2007,2012 | 33.8 (32.1-35.6) | 35.2 (32.5-37.9) | 1.4 | 0.7 | 0.14 |
| Chile | Subnational | 2000,2003,2008,2016 | 75.1 (73.3-76.8) | 18.6 (17.3-20.0) | -56.5 | -17.7 | <0.001 |  | 2000,2003,2008,2016 | 36.1 (34.2-37.9) | 27.9 (26.4-29.4) | -8.2 | -2.6 | <0.001 |
| Costa Rica | National | 1999,2002,2008,2013 | 61.6 (59.7-63.5) | 12.7 (11.3-14.1) | -48.9 | -17.5 | <0.001 |  | 1999,2002,2008,2013 | 44.1 (42.4-45.7) | 29.0 (25.6-32.3) | -15.1 | -5.4 | <0.001 |
| Cuba | National | 2000,2004,2010,2018 | 22.0 (18.9-25.1) | 18.5 (17.2-19.8) | -3.5 | -1.0 | 0.17 |  | 2000,2004,2010,2018 | 64.3 (61.0-67.5) | 42.3 (39.8-44.8) | -22.0 | -6.1 | <0.001 |
| Dominica | National | 2000,2004,2009 | 13.8 (11.4-16.1) | 11.6 (9.7-13.5) | -2.2 | -1.2 | 0.29 |  | 2000,2004,2009 | 41.6 (38.2-44.9) | 33.9 (30.1-37.8) | -7.7 | -4.3 | 0.003 |
| Dominican Republic | National | NA | NA | NA | NA | NA | NA |  | 2004,2011,2016 | 51.1 (48.4-53.9) | 44.8 (38.3-51.3) | -6.3 | -2.6 | 0.08 |
| Ecuador | Subnational | NA | NA | NA | NA | NA | NA |  | 2001,2007,2016 | 54.6 (50.7-58.5) | 42.8 (39.1-46.4) | -11.8 | -3.9 | <0.001 |
| El Salvador | National | NA | NA | NA | NA | NA | NA |  | 2003,2009,2015 | 56.1 (44.4-67.8) | 33.3 (30.6-36.0) | -22.8 | -9.5 | <0.001 |
| Grenada | National | 2000,2004,2009 | 11.1 (8.6-13.6) | 12.4 (10.8-14.1) | 1.3 | 0.7 | 0.36 |  | 2000,2004,2009,2016 | 40.1 (37.0-43.2) | 28.9 (26.0-31.9) | -11.2 | -3.5 | <0.001 |
| Guatemala | National | 2002,2008,2015 | 68.7 (65.2-72.2) | 24.6 (23.1-26.1) | -44.1 | -17.0 | <0.001 |  | 2002,2008,2015 | 41.3 (37.2-45.4) | 36.3 (32.9-39.7) | -5.0 | -1.9 | 0.002 |
| Guyana | National | 2000,2004,2010 | 13.1 (9.5-16.6) | 12.6 (10.8-14.5) | -0.5 | -0.2 | 0.65 |  | 2000,2004,2010,2015 | 37.4 (32.3-42.6) | 48.7 (42.5-54.9) | 11.3 | 3.8 | 0.17 |
| Jamaica | National | 2000,2006,2010 | 14.7 (12.0-17.4) | 18.3 (14.5-22.1) | 3.6 | 1.8 | 0.09 |  | 2000,2006,2010,2017 | 39.8 (34.4-45.1) | 34.3 (31.8-36.8) | -5.5 | -1.6 | 0.30 |
| Mexico | Subnational | 2003,2005,2006,2008,2011 | 35.3 (33.3-37.3) | 23.3 (20.8-25.7) | -12.0 | -7.5 | <0.001 |  | 2003,2005,2006,2008,2011 | 40.6 (38.5-42.6) | 39.6 (36.6-42.7) | -1.0 | -0.6 | 0.36 |
| Nicaragua | National | 2003,2014,2019 | 75.3 (73.4-77.3) | 12.2 (11.0-13.5) | -63.1 | -19.7 | <0.001 |  | 2003,2014,2019 | 49.6 (47.3-52.0) | 44.2 (41.4-47.0) | -5.4 | -1.7 | <0.001 |
| Panama | National | 2002,2008,2012 | 24.7 (21.7-27.7) | 20.0 (18.4-21.5) | -4.7 | -2.3 | 0.001 |  | 2002,2008,2012,2017 | 67.7 (65.1-70.3) | 47.9 (43.7-52.2) | -19.8 | -6.6 | <0.001 |
| Paraguay | National | 2003,2008,2014,2019 | 81.2 (80.0-82.4) | 16.2 (14.7-17.8) | -65.0 | -20.3 | <0.001 |  | 2003,2008,2014,2019 | 50.0 (48.1-51.8) | 41.6 (38.6-44.5) | -8.4 | -2.6 | <0.001 |
| Peru | National | 2000,2002,2003,2007 | 86.1 (84.8-87.5) | 86.5 (84.6-88.4) | 0.4 | 0.3 | 0.51 |  | 2000,2002,2003,2007,2014,2019 | 61.3 (58.4-64.3) | 34.1 (31.7-36.5) | -27.2 | -7.2 | <0.001 |
| Saint Lucia | National | 2000,2007,2011 | 12.2 (10.7-13.7) | 15.0 (12.2-17.8) | 2.8 | 1.3 | 0.09 |  | 2000,2007,2011,2017 | 32.1 (29.1-35.2) | 38.8 (35.7-41.9) | 6.7 | 2.0 | 0.11 |
| Saint Vincent and the Grenadines | National | 2000,2007,2011 | 14.0 (12.0-16.0) | 18.1 (15.7-20.6) | 4.1 | 1.9 | 0.01 |  | 2000,2007,2011,2018 | 34.8 (32.0-37.7) | 35.2 (32.1-38.3) | 0.4 | 0.1 | 0.13 |
| Suriname | National | 2000,2004,2009 | 25.3 (22.6-27.9) | 18.9 (15.6-22.2) | -6.4 | -3.6 | 0.005 |  | 2000,2004,2009,2016 | 32.3 (29.3-35.2) | 26.7 (23.7-29.6) | -5.6 | -1.7 | 0.79 |
| Trinidad and Tobago | National | 2000,2007,2011,2017 | 13.1 (11.8-14.5) | 16.0 (14.5-17.5) | 2.9 | 0.9 | <0.001 |  | 2000,2007,2011,2017 | 27.8 (24.6-31.0) | 30.0 (25.6-34.3) | 2.2 | 0.6 | 0.43 |
| Uruguay | National | 2000,2007,2014 | 71.0 (69.1-73.0) | 17.1 (15.8-18.3) | -53.9 | -19.2 | <0.001 |  | 2000,2007,2014 | 36.4 (34.2-38.7) | 33.4 (31.6-35.2) | -3.0 | -1.1 | 0.25 |
| Venezuela | National | 1999,2001,2003,2008 | 82.3 (81.1-83.6) | 85.7 (83.3-88.2) | 3.4 | 1.9 | 0.48 |  | 1999,2001,2003,2008,2019 | 53.4 (51.3-55.6) | 40.4 (36.4-44.4) | -13.0 | -3.2 | <0.001 |
| **Eastern Mediterranean** | |  |  |  |  |  |  |  |  |  |  |  |  |  |
| Afghanistan | Subnational | NA | NA | NA | NA | NA | NA |  | 2004,2010,2017 | 57.2 (49.8-64.6) | 37.3 (33.4-41.1) | -19.9 | -7.7 | <0.001 |
| Djibouti | National | NA | NA | NA | NA | NA | NA |  | 2003,2009,2013 | 55.0 (50.8-59.2) | 58.3 (53.4-63.3) | 3.3 | 1.6 | 0.52 |
| Egypt | National | 2001,2005,2009,2014 | 21.4 (18.6-24.2) | 19.8 (12.0-27.5) | -1.6 | -0.6 | 0.98 |  | 2001,2005,2014 | 56.8 (51.4-62.3) | 57.1 (45.1-69.0) | 0.3 | 0.1 | 0.85 |
| Gaza Strip | Subnational | NA | NA | NA | NA | NA | NA |  | 2000,2005,2013,2019 | 52.5 (47.7-57.2) | 50.2 (43.6-56.7) | -2.3 | -0.6 | 0.82 |
| Iraq | National | NA | NA | NA | NA | NA | NA |  | 2006,2008,2014,2019 | 76.0 (73.0-78.9) | 54.7 (50.8-58.5) | -21.3 | -8.2 | <0.001 |
| Jordan | National | 1999,2003,2007,2009 | 13.8 (12.3-15.4) | 49.7 (47.2-52.2) | 35.9 | 18.0 | <0.001 |  | 1999,2003,2007,2014 | 54.3 (51.8-56.7) | 35.7 (31.5-40.0) | -18.6 | -6.2 | <0.001 |
| Kuwait | National | 2001,2005,2009 | 25.7 (24.2-27.2) | 69.0 (67.1-70.9) | 43.3 | 27.1 | <0.001 |  | 2001,2005,2016 | 50.6 (48.8-52.4) | 32.1 (28.8-35.4) | -18.5 | -6.2 | <0.001 |
| Lebanon | National | NA | NA | NA | NA | NA | NA |  | 2001,2005,2011 | 24.4 (22.7-26.1) | 21.7 (18.8-24.6) | -2.7 | -1.3 | 0.005 |
| Libyan Arab Jamahiriya | National | 2003,2007,2010 | 49.1 (45.5-52.8) | 40.0 (36.5-43.4) | -9.1 | -6.5 | 0.001 |  | NA | NA | NA | NA | NA | NA |
| Morocco | National | 2001,2006,2010 | 12.3 (10.7-13.9) | 76.1 (74.0-78.2) | 63.8 | 35.4 | <0.001 |  | 2001,2006,2016 | 66.1 (63.0-69.1) | 44.1 (41.1-47.1) | -22.0 | -7.3 | <0.001 |
| Oman | National | NA | NA | NA | NA | NA | NA |  | NA | NA | NA | NA | NA | NA |
| Pakistan | National | 2004,2008,2013 | 69.6 (64.5-74.7) | 32.3 (27.7-36.9) | -37.3 | -20.7 | <0.001 |  | 2004,2008,2013 | 70.8 (65.7-75.9) | 61.2 (56.7-65.7) | -9.6 | -5.3 | 0.007 |
| Qatar | National | NA | NA | NA | NA | NA | NA |  | 2004,2007,2013,2018 | 43.0 (41.1-45.0) | 36.3 (31.5-41.1) | -6.7 | -2.4 | <0.001 |
| Saudi Arabia | National | 2001,2007,2010 | 16.0 (13.1-19.0) | 44.8 (41.7-48.0) | 28.8 | 16.0 | <0.001 |  | NA | NA | NA | NA | NA | NA |
| Sudan | National | 2001,2005,2009 | 21.4 (19.5-23.3) | 68.3 (65.1-71.5) | 46.9 | 29.3 | <0.001 |  | NA | NA | NA | NA | NA | NA |
| Syrian Arab Republic | National | 2002,2007,2010 | 9.0 (7.4-10.7) | 55.7 (51.2-60.1) | 46.7 | 29.2 | <0.001 |  | NA | NA | NA | NA | NA | NA |
| Tunisia | National | NA | NA | NA | NA | NA | NA |  | 2001,2007,2017 | 44.5 (42.0-47.0) | 43.3 (39.7-46.8) | -1.2 | -0.4 | 0.50 |
| United Arab Emirates | National | 2002,2005,2013 | 41.0 (38.1-43.8) | 14.4 (12.7-16.0) | -26.6 | -12.1 | <0.001 |  | 2002,2005,2013 | 43.6 (40.3-46.9) | 29.3 (26.3-32.2) | -14.3 | -6.5 | <0.001 |
| West BANK | regional | 2000,2005,2009 | 48.0 (45.9-50.2) | 76.6 (74.2-79.0) | 28.6 | 15.9 | <0.001 |  | 2000,2005,2016 | 55.2 (53.6-56.7) | 42.9 (37.0-48.7) | -12.3 | -3.8 | 0.007 |
| Yemen | National | NA | NA | NA | NA | NA | NA |  | 2003,2008,2014 | 60.4 (58.2-62.5) | 47.1 (40.4-53.8) | -13.3 | -6.0 | <0.001 |
| **Europe** |  |  |  |  |  |  |  |  |  |  |  |  |  |  |
| Albania | National | 2004,2009,2015 | 59.0 (56.7-61.3) | 47.5 (45.4-49.6) | -11.5 | -5.2 | <0.001 |  | NA | NA | NA | NA | NA | NA |
| Croatia (Hrvatska) | National | 2003,2007,2011 | 55.0 (52.9-57.2) | 41.9 (39.6-44.2) | -13.1 | -8.2 | <0.001 |  | NA | NA | NA | NA | NA | NA |
| Czech Republic | National | 2002,2007,2011 | 22.5 (21.0-24.0) | 21.2 (19.5-23.0) | -1.3 | -0.7 | 0.15 |  | 2002,2007,2016 | 21.6 (19.8-23.3) | 11.6 (10.1-13.1) | -10.0 | -3.6 | <0.001 |
| Kazakhstan | National | 2004,2009,2014 | 38.0 (35.8-40.2) | 44.5 (37.4-51.5) | 6.5 | 3.2 | 0.11 |  | NA | NA | NA | NA | NA | NA |
| Latvia | National | 2002,2007,2011,2014 | 42.7 (39.9-45.4) | 39.9 (38.1-41.7) | -2.8 | -1.2 | 0.08 |  | 2002,2014,2019 | 25.2 (22.5-27.9) | 17.9 (15.6-20.1) | -7.3 | -2.1 | 0.001 |
| Lithuania | National | 2001,2005,2009 | 19.1 (16.7-21.5) | 16.8 (14.9-18.7) | -2.3 | -1.4 | 0.07 |  | 2001,2005,2009,2014,2018 | 49.2 (46.7-51.6) | 51.1 (48.3-53.9) | 1.9 | 0.6 | 0.27 |
| Poland | National | 1999,2003,2009,2016 | 23.3 (21.5-25.0) | 16.7 (15.4-18.0) | -6.6 | -1.9 | 0.004 |  | NA | NA | NA | NA | NA | NA |
| Russian Federation | Subnational | 1999,2002,2004 | 36.1 (34.0-38.1) | 29.1 (26.4-31.8) | -7.0 | -7.0 | <0.001 |  | 1999,2002,2015 | 24.3 (22.9-25.7) | 15.7 (14.0-17.5) | -8.6 | -2.7 | <0.001 |
| Slovakia | National | 2003,2007,2011,2016 | 22.2 (20.8-23.6) | 18.4 (17.3-19.4) | -3.8 | -1.5 | <0.001 |  | NA | NA | NA | NA | NA | NA |
| Slovenia | National | 2003,2007,2011 | 29.0 (27.2-30.8) | 26.4 (24.1-28.8) | -2.6 | -1.6 | 0.05 |  | NA | NA | NA | NA | NA | NA |
| Turkey | National | 2003,2009,2012 | 46.0 (44.8-47.2) | 70.1 (67.5-72.6) | 24.1 | 13.4 | <0.001 |  | NA | NA | NA | NA | NA | NA |
| Ukraine | National | 1999,2005,2011,2017 | 37.0 (34.9-39.1) | 46.3 (41.7-51.0) | 9.3 | 2.6 | <0.001 |  | NA | NA | NA | NA | NA | NA |
| **South-East Asia** | |  |  |  |  |  |  |  |  |  |  |  |  |  |
| Bangladesh | National | NA | NA | NA | NA | NA | NA |  | 2004,2007,2013 | 58.6 (55.2-62.0) | 45.0 (40.0-50.1) | -13.6 | -7.6 | <0.001 |
| Bhutan | National | 2004,2006,2009 | 17.2 (13.8-20.6) | 26.0 (22.1-29.9) | 8.8 | 8.8 | 0.001 |  | 2004,2006,2009,2013,2019 | 48.2 (43.4-52.9) | 30.5 (27.6-33.3) | -17.7 | -5.9 | <0.001 |
| India | National | 2000,2002,2003,2004,2009 | 41.2 (39.3-43.2) | 48.7 (45.9-51.4) | 7.5 | 4.2 | <0.001 |  | 2000,2002,2003,2004,2006,2009 | 83.7 (81.8-85.7) | 57.1 (53.5-60.7) | -26.6 | -14.8 | <0.001 |
| Indonesia | National | 2000,2004,2005,2006,2009,2014,2019 | 18.7 (16.6-20.8) | 28.1 (26.0-30.2) | 9.4 | 2.5 | 0.003 |  | 2000,2004,2005,2006,2009,2014,2019 | 31.1 (27.7-34.6) | 20.4 (18.2-22.5) | -10.7 | -2.8 | <0.001 |
| Maldives | National | NA | NA | NA | NA | NA | NA |  | 2004,2007,2011,2019 | 30.1 (26.6-33.6) | 29.4 (27.1-31.7) | -0.7 | -0.2 | <0.001 |
| Myanmar | National | 2001,2004,2007,2011 | 48.8 (46.5-51.1) | 49.5 (46.3-52.7) | 0.7 | 0.4 | 0.006 |  | 2001,2007,2011,2016 | 46.4 (43.9-48.9) | 28.7 (25.6-31.8) | -17.7 | -5.9 | <0.001 |
| Nepal | National | 2001,2004,2007,2011 | 54.3 (46.2-62.5) | 32.9 (28.3-37.6) | -21.4 | -10.7 | <0.001 |  | 2001,2004,2007,2011 | 32.8 (29.1-36.5) | 29.9 (26.2-33.6) | -2.9 | -1.4 | <0.001 |
| Sri Lanka | National | 1999,2003,2007,2011,2015 | 16.9 (13.5-20.2) | 12.9 (10.6-15.1) | -4.0 | -1.2 | 0.71 |  | 1999,2003,2007,2011,2015 | 59.4 (56.8-62.0) | 63.3 (57.9-68.7) | 3.9 | 1.2 | <0.001 |
| Thailand | National | NA | NA | NA | NA | NA | NA |  | 2005,2009,2015 | 63.4 (61.1-65.7) | 47.7 (43.1-52.2) | -15.7 | -7.8 | <0.001 |
| Timor-Leste | National | NA | NA | NA | NA | NA | NA |  | 2006,2009,2013,2019 | 55.5 (51.6-59.3) | 53.3 (50.3-56.4) | -2.2 | -0.8 | <0.001 |
| **Western Pacific** | |  |  |  |  |  |  |  |  |  |  |  |  |  |
| Cambodia | National | NA | NA | NA | NA | NA | NA |  | 2003,2010,2016 | 31.5 (27.6-35.5) | 24.1 (21.4-26.9) | -7.4 | -2.8 | 0.004 |
| Cook Islands | National | 2003,2008,2016 | 28.6 (24.6-32.7) | 21.6 (17.9-25.3) | -7.0 | -2.7 | <0.001 |  | 2003,2008,2016 | 45.5 (40.8-50.1) | 36.3 (32.0-40.6) | -9.2 | -3.5 | 0.02 |
| Fiji | National | 1999,2005,2009,2016 | 19.5 (16.9-22.1) | 18.1 (14.6-21.5) | -1.4 | -0.4 | 1.00 |  | 1999,2005,2009,2016 | 41.9 (37.2-46.6) | 40.0 (35.4-44.6) | -1.9 | -0.6 | 0.43 |
| Guam | National | NA | NA | NA | NA | NA | NA |  | 2011,2014,2017 | 27.6 (23.7-31.5) | 28.5 (25.5-31.5) | 0.9 | 0.7 | 0.18 |
| Laos | National | 2003,2007,2011 | 40.9 (39.1-42.7) | 31.0 (27.6-34.4) | -9.9 | -6.2 | <0.001 |  | 2003,2007,2011,2016 | 50.5 (48.6-52.4) | 68.6 (65.3-72.0) | 18.1 | 7.0 | <0.001 |
| Macau (China) | National | 2001,2005,2010,2015 | 43.6 (41.1-46.1) | 13.2 (10.8-15.6) | -30.4 | -10.9 | <0.001 |  | 2001,2005,2010,2015 | 26.1 (23.2-29.1) | 15.4 (12.8-18.0) | -10.7 | -3.8 | <0.001 |
| Mongolia | National | NA | NA | NA | NA | NA | NA |  | 2003,2007,2014,2019 | 33.7 (31.2-36.3) | 60.0 (56.8-63.3) | 26.3 | 8.2 | <0.001 |
| Micronesia | National | NA | NA | NA | NA | NA | NA |  | 2007,2013,2019 | 52.8 (46.4-59.1) | 48.0 (45.9-50.0) | -4.8 | -2.0 | 0.14 |
| New Zealand | National | NA | NA | NA | NA | NA | NA |  | 2007,2008,2010 | 17.2 (10.6-23.8) | 15.9 (11.6-20.2) | -1.3 | -2.2 | 0.88 |
| Philippines | National | 2000,2004,2007,2011,2015,2019 | 20.9 (19.4-22.5) | 16.9 (15.6-18.2) | -4.0 | -1.1 | <0.001 |  | 2000,2004,2007,2011,2015,2019 | 55.6 (53.6-57.6) | 41.1 (39.1-43.1) | -14.5 | -3.8 | <0.001 |
| South Korea | National | NA | NA | NA | NA | NA | NA |  | 2005,2008,2013 | 32.0 (29.4-34.6) | 27.1 (24.9-29.4) | -4.9 | -3.1 | <0.001 |

Abbreviation: NA, not available.

**Table S13 Trends in the proportions of adolescents aged 12–16 years who believe that tobacco smoking helps people feel more comfortable and makes it easier to make friends, from 1999 to 2019, by country/territory.**

| **Country/territory** | **Tobacco smoking helps people feel more comfortable** | | | | | | |  | **Tobacco smoking makes more friends** | | | | | |
| --- | --- | --- | --- | --- | --- | --- | --- | --- | --- | --- | --- | --- | --- | --- |
|  | **Representativeness** | **Survey year** | **First year, %**  **(95%CI)** | **Last year, %**  **(95%CI)** | **Total absolute change, %** | **Absolute change/5-years, %** | ***P* for trend** |  | **Survey year** | **First year, %**  **(95%CI)** | **Last year, %**  **(95%CI)** | **Total absolute change, %** | **Absolute change/5-years, %** | ***P* for trend** |
| **Africa** |  |  |  |  |  |  |  |  |  |  |  |  |  |  |
| Burkina Faso | Subnational | 2001,2006,2009 | 30.6 (25.7-35.6) | 21.7 (19.8-23.6) | -8.9 | -5.6 | 0.001 |  | 2001,2006,2009 | 43.4 (36.5-50.3) | 27.6 (25.1-30.1) | -15.8 | -9.9 | <0.001 |
| Congo | National | 2006,2009,2019 | 32.5 (27.0-38.0) | 15.5 (12.7-18.4) | -17.0 | -6.5 | <0.001 |  | 2006,2009,2019 | 44.4 (41.2-47.5) | 26.1 (23.2-29.0) | -18.3 | -7.0 | <0.001 |
| Ghana | National | 2000,2006,2009,2017 | 27.3 (21.2-33.5) | 17.9 (15.2-20.6) | -9.4 | -2.8 | <0.001 |  | 2000,2006,2009 | 45.9 (40.8-51.1) | 43.0 (40.5-45.5) | -2.9 | -1.6 | 0.26 |
| Kenya | National | 2001,2007,2013 | 17.2 (12.8-21.5) | 15.3 (13.1-17.6) | -1.9 | -0.8 | 0.28 |  | NA | NA | NA | NA | NA | NA |
| Malawi | National | 2000,2005,2009 | 30.7 (27.1-34.3) | 21.5 (17.6-25.4) | -9.2 | -5.1 | 0.09 |  | 2000,2005,2009 | 46.9 (42.7-51.0) | 34.2 (28.3-40.1) | -12.7 | -7.1 | 0.08 |
| Mauritania | National | 2001,2006,2009,2018 | 30.6 (27.2-33.9) | 21.0 (15.4-26.5) | -9.6 | -2.8 | 0.001 |  | 2001,2006,2009,2018 | 47.3 (42.7-51.8) | 27.7 (25.0-30.5) | -19.6 | -5.8 | <0.001 |
| Mauritius | National | 2003,2008,2016 | 24.4 (22.4-26.4) | 17.7 (14.3-21.0) | -6.7 | -2.6 | 0.001 |  | 2003,2008,2016 | 36.6 (32.2-40.9) | 28.0 (22.8-33.1) | -8.6 | -3.3 | 0.006 |
| Mozambique | National | 2002,2007,2013 | 33.7 (31.0-36.3) | 23.0 (20.9-25.2) | -10.7 | -4.9 | <0.001 |  | NA | NA | NA | NA | NA | NA |
| Niger | National | 2001,2006,2009 | 34.6 (29.7-39.5) | 42.0 (35.9-48.0) | 7.4 | 4.6 | 0.04 |  | 2001,2006,2009 | 42.5 (38.4-46.6) | 46.2 (39.1-53.3) | 3.7 | 2.3 | 0.31 |
| Senegal | National | 2002,2007,2013 | 25.1 (22.4-27.8) | 24.1 (19.0-29.1) | -1.0 | -0.5 | 0.43 |  | NA | NA | NA | NA | NA | NA |
| Seychelles | National | 2002,2007,2015 | 31.1 (26.1-36.1) | 27.2 (24.5-29.9) | -3.9 | -1.5 | 0.11 |  | NA | NA | NA | NA | NA | NA |
| South Africa | National | 1999,2002,2008,2011 | 33.9 (28.8-39.0) | 46.4 (43.4-49.3) | 12.5 | 5.2 | <0.001 |  | 1999,2002,2008,2011 | 54.6 (49.1-60.2) | 58.2 (54.2-62.2) | 3.6 | 1.5 | 0.18 |
| Swaziland | National | 2001,2005,2009 | 34.9 (32.6-37.1) | 40.1 (36.8-43.4) | 5.2 | 3.3 | 0.009 |  | 2001,2005,2009 | 61.3 (58.9-63.7) | 61.2 (57.4-65.1) | -0.1 | -0.1 | 0.87 |
| Togo | National | 2002,2007,2013,2019 | 16.8 (13.4-20.3) | 8.0 (4.4-11.6) | -8.8 | -2.6 | <0.001 |  | 2002,2007,2013,2019 | 21.6 (18.3-25.0) | 14.3 (11.0-17.6) | -7.3 | -2.1 | <0.001 |
| Uganda | National | 2002,2007,2011,2018 | 24.4 (22.6-26.3) | 16.7 (14.3-19.1) | -7.7 | -2.4 | 0.06 |  | 2002,2007,2011 | 32.9 (30.1-35.6) | 30.3 (25.6-34.9) | -2.6 | -1.4 | 0.39 |
| United Republic of Tanzania | Subnational | 2003,2008,2016 | 44.8 (42.1-47.6) | 23.6 (20.2-27.0) | -21.2 | -8.2 | <0.001 |  | NA | NA | NA | NA | NA | NA |
| Zambia | National | 2002,2007,2011 | 28.8 (25.8-31.8) | 31.7 (30.0-33.5) | 2.9 | 1.6 | 0.02 |  | 2002,2007,2011 | 41.8 (38.0-45.5) | 37.9 (36.1-39.8) | -3.9 | -2.2 | 0.002 |
| Zimbabwe | National | 1999,2003,2008,2014 | 28.5 (26.3-30.7) | 22.2 (18.1-26.4) | -6.3 | -2.1 | 0.01 |  | 1999,2003,2008,2014 | 47.8 (44.1-51.5) | 22.2 (19.2-25.1) | -25.6 | -8.5 | <0.001 |
| **Americas** |  |  |  |  |  |  |  |  |  |  |  |  |  |  |
| Antigua and Barbuda | National | 2000,2004,2009,2017 | 27.5 (24.6-30.3) | 34.6 (32.0-37.3) | 7.1 | 2.1 | 0.002 |  | 2000,2004,2009,2017 | 30.3 (27.6-33.0) | 27.4 (25.0-29.8) | -2.9 | -0.9 | 0.21 |
| Argentina | National | 2000,2007,2012,2018 | 20.3 (17.8-22.9) | 24.4 (18.7-30.0) | 4.1 | 1.1 | 0.29 |  | 2000,2007,2018 | 12.0 (9.6-14.4) | 12.8 (9.5-16.0) | 0.8 | 0.2 | 0.002 |
| Bahamas | National | 2000,2004,2009,2013 | 37.9 (33.8-42.1) | 28.1 (21.8-34.5) | -9.8 | -3.8 | 0.005 |  | 2000,2004,2009 | 37.9 (34.8-41.0) | 45.3 (42.8-47.9) | 7.4 | 4.1 | 0.76 |
| Barbados | National | 1999,2002,2007,2013 | 38.4 (34.8-42.0) | 42.8 (39.4-46.2) | 4.4 | 1.6 | 0.008 |  | 1999,2002,2007 | 28.5 (24.4-32.5) | 45.3 (42.0-48.7) | 16.8 | 10.5 | <0.001 |
| Belize | National | 2002,2008,2014 | 34.4 (29.6-39.1) | 33.1 (29.5-36.6) | -1.3 | -0.5 | 0.57 |  | NA | NA | NA | NA | NA | NA |
| Bolivia | Subnational | 2000,2003,2012,2018 | 33.7 (32.4-34.9) | 30.5 (28.2-32.8) | -3.2 | -0.9 | 0.002 |  | 2000,2003,2012 | 22.4 (21.2-23.7) | 29.2 (23.8-34.7) | 6.8 | 2.8 | 0.06 |
| Brazil | Subnational | 2002,2005,2006,2007 | 30.5 (29.1-31.9) | 34.1 (30.1-38.0) | 3.6 | 3.6 | <0.001 |  | 2002,2005,2006,2007 | 16.6 (15.1-18.0) | 21.5 (17.8-25.3) | 4.9 | 4.9 | <0.001 |
| Chile | Subnational | 2000,2003,2008,2016 | 40.7 (37.1-44.2) | 37.3 (35.8-38.8) | -3.4 | -1.1 | 0.50 |  | 2000,2003,2008 | 21.9 (20.0-23.8) | 29.1 (27.9-30.3) | 7.2 | 4.5 | <0.001 |
| Costa Rica | National | 1999,2002,2008,2013 | 16.2 (14.9-17.5) | 27.9 (25.5-30.3) | 11.7 | 4.2 | <0.001 |  | 1999,2002,2008,2013 | 20.9 (19.3-22.6) | 18.7 (16.4-21.0) | -2.2 | -0.8 | 0.05 |
| Cuba | National | 2000,2004,2010,2018 | 20.6 (17.1-24.0) | 17.5 (15.5-19.5) | -3.1 | -0.9 | <0.001 |  | 2000,2004,2010,2018 | 12.0 (10.3-13.8) | 12.8 (11.3-14.3) | 0.8 | 0.2 | 0.15 |
| Dominica | National | 2000,2004,2009 | 30.5 (27.6-33.4) | 35.1 (32.3-37.9) | 4.6 | 2.6 | 0.006 |  | 2000,2004,2009 | 35.6 (32.3-38.9) | 41.4 (38.0-44.7) | 5.8 | 3.2 | 0.02 |
| Dominican Republic | National | 2004,2011,2016 | 22.5 (20.8-24.2) | 32.1 (27.6-36.6) | 9.6 | 4.0 | 0.02 |  | NA | NA | NA | NA | NA | NA |
| Ecuador | Subnational | 2001,2007,2016 | 22.3 (19.5-25.0) | 35.5 (32.2-38.7) | 13.2 | 4.4 | <0.001 |  | NA | NA | NA | NA | NA | NA |
| El Salvador | National | 2003,2009,2015 | 25.2 (18.9-31.5) | 32.8 (30.3-35.3) | 7.6 | 3.2 | 0.02 |  | NA | NA | NA | NA | NA | NA |
| Grenada | National | 2000,2004,2009,2016 | 22.8 (20.1-25.6) | 32.1 (29.6-34.7) | 9.3 | 2.9 | <0.001 |  | 2000,2004,2009 | 31.2 (27.9-34.5) | 44.7 (42.6-46.7) | 13.5 | 7.5 | <0.001 |
| Guatemala | National | 2002,2008,2015 | 32.1 (29.2-35.0) | 36.5 (34.1-38.8) | 4.4 | 1.7 | 0.03 |  | 2002,2008,2015 | 25.6 (22.0-29.3) | 30.5 (28.1-32.8) | 4.9 | 1.9 | 0.22 |
| Guyana | National | 2000,2004,2010,2015 | 27.4 (21.0-33.8) | 27.4 (23.9-31.0) | 0.0 | 0.0 | 0.06 |  | 2000,2004,2010 | 30.1 (23.8-36.4) | 41.8 (36.9-46.6) | 11.7 | 5.8 | 0.002 |
| Jamaica | National | 2000,2006,2010,2017 | 30.7 (26.8-34.7) | 45.7 (43.0-48.3) | 15.0 | 4.4 | 0.001 |  | 2000,2006,2010 | 36.6 (32.0-41.2) | 46.1 (40.6-51.7) | 9.5 | 4.8 | 0.005 |
| Mexico | Subnational | 2003,2005,2006,2008,2011 | 13.8 (12.7-14.9) | 30.7 (27.5-34.0) | 16.9 | 10.6 | <0.001 |  | 2003,2005,2006,2008,2011 | 42.8 (40.5-45.2) | 31.5 (28.8-34.1) | -11.3 | -7.1 | <0.001 |
| Nicaragua | National | 2003,2014,2019 | 30.2 (28.7-31.8) | 21.6 (20.0-23.2) | -8.6 | -2.7 | <0.001 |  | 2003,2014,2019 | 28.2 (25.7-30.7) | 25.5 (23.4-27.7) | -2.7 | -0.8 | 0.10 |
| Panama | National | 2002,2008,2012,2017 | 22.2 (20.0-24.4) | 21.6 (19.2-23.9) | -0.6 | -0.2 | 0.28 |  | 2002,2008,2012 | 24.0 (22.1-26.0) | 17.1 (15.4-18.7) | -6.9 | -3.4 | <0.001 |
| Paraguay | National | 2003,2008,2014,2019 | 23.1 (21.4-24.8) | 29.0 (27.1-30.9) | 5.9 | 1.8 | <0.001 |  | 2003,2008,2014,2019 | 15.6 (13.9-17.2) | 20.1 (18.2-22.0) | 4.5 | 1.4 | <0.001 |
| Peru | National | 2000,2002,2003,2007,2014,2019 | 38.2 (35.4-41.0) | 23.3 (21.0-25.5) | -14.9 | -3.9 | <0.001 |  | 2000,2002,2003,2007 | 16.8 (14.7-18.9) | 23.4 (21.4-25.4) | 6.6 | 4.7 | <0.001 |
| Saint Lucia | National | 2000,2007,2011,2017 | 26.9 (24.2-29.6) | 33.8 (30.9-36.7) | 6.9 | 2.0 | <0.001 |  | 2000,2007,2011,2017 | 38.6 (35.3-41.9) | 29.0 (25.8-32.1) | -9.6 | -2.8 | <0.001 |
| Saint Vincent and the Grenadines | National | 2000,2007,2011,2018 | 28.0 (24.9-31.0) | 40.3 (36.4-44.1) | 12.3 | 3.4 | <0.001 |  | 2000,2007,2011 | 32.7 (29.8-35.5) | 41.3 (38.6-43.9) | 8.6 | 3.9 | <0.001 |
| Suriname | National | 2000,2004,2009,2016 | 29.8 (25.8-33.8) | 44.8 (41.6-48.1) | 15.0 | 4.7 | <0.001 |  | 2000,2004,2009,2016 | 32.6 (28.8-36.3) | 41.0 (37.7-44.3) | 8.4 | 2.6 | <0.001 |
| Trinidad and Tobago | National | 2000,2007,2011,2017 | 33.9 (31.4-36.4) | 31.7 (28.7-34.7) | -2.2 | -0.6 | 0.54 |  | 2000,2007,2011 | 34.4 (31.7-37.2) | 34.9 (31.9-37.9) | 0.5 | 0.2 | 1.00 |
| Uruguay | National | 2000,2007,2014 | 20.4 (18.1-22.7) | 22.9 (20.8-25.0) | 2.5 | 0.9 | 0.45 |  | NA | NA | NA | NA | NA | NA |
| Venezuela | National | 1999,2001,2003,2008,2019 | 20.9 (19.3-22.5) | 25.5 (22.8-28.3) | 4.6 | 1.2 | 0.03 |  | 1999,2001,2003,2008,2010 | 14.0 (12.5-15.5) | 11.6 (9.8-13.3) | -2.4 | -1.1 | 0.05 |
| **Eastern Mediterranean** | |  |  |  |  |  |  |  |  |  |  |  |  |  |
| Djibouti | National | 2003,2009,2013 | 35.1 (31.8-38.4) | 32.5 (27.6-37.4) | -2.6 | -1.3 | 0.38 |  | NA | NA | NA | NA | NA | NA |
| Egypt | National | 2005,2009,2014 | 9.1 (7.4-10.9) | 20.2 (13.1-27.3) | 11.1 | 6.2 | 0.008 |  | 2001,2005,2014 | 29.2 (25.4-33.0) | 34.0 (23.6-44.5) | 4.8 | 1.8 | 0.35 |
| Gaza Strip | Subnational | 2005,2013,2019 | 20.4 (17.0-23.9) | 18.7 (14.2-23.2) | -1.7 | -0.6 | 0.54 |  | NA | NA | NA | NA | NA | NA |
| Iraq | National | 2006,2008,2014,2019 | 20.8 (17.7-24.0) | 23.4 (19.2-27.7) | 2.6 | 1.0 | 0.08 |  | 2006,2008,2014 | 36.8 (33.0-40.6) | 25.8 (20.6-31.1) | -11.0 | -6.9 | 0.007 |
| Jordan | National | 1999,2003,2007,2009,2014 | 20.0 (18.2-21.7) | 24.4 (21.0-27.8) | 4.4 | 1.5 | 0.11 |  | 1999,2003,2007,2014 | 37.9 (35.1-40.7) | 36.6 (33.5-39.6) | -1.3 | -0.4 | 0.35 |
| Kuwait | National | 2001,2005,2009,2016 | 18.4 (16.8-19.9) | 23.3 (21.5-25.0) | 4.9 | 1.6 | <0.001 |  | 2001,2005,2009,2016 | 32.3 (30.3-34.3) | 25.0 (22.2-27.7) | -7.3 | -2.4 | <0.001 |
| Lebanon | National | 2001,2005,2011 | 25.8 (22.7-28.9) | 18.6 (16.7-20.5) | -7.2 | -3.6 | <0.001 |  | 2001,2005,2011 | 19.8 (18.2-21.5) | 20.9 (17.5-24.2) | 1.1 | 0.5 | 0.31 |
| Libyan Arab Jamahiriya | National | 2003,2007,2010 | 19.5 (16.9-22.2) | 13.5 (10.6-16.3) | -6.0 | -4.3 | 0.004 |  | NA | NA | NA | NA | NA | NA |
| Oman | National | 2002,2010,2016 | 17.3 (14.8-19.8) | 18.1 (13.9-22.3) | 0.8 | 0.3 | 0.82 |  | NA | NA | NA | NA | NA | NA |
| Pakistan | National | 2004,2008,2013 | 8.0 (6.0-10.0) | 62.4 (54.9-69.9) | 54.4 | 30.2 | <0.001 |  | 2004,2008,2013 | 26.5 (23.2-29.7) | 19.6 (15.9-23.2) | -6.9 | -3.8 | 0.006 |
| Qatar | National | 2004,2007,2013,2018 | 18.1 (16.6-19.7) | 22.7 (20.1-25.3) | 4.6 | 1.6 | <0.001 |  | NA | NA | NA | NA | NA | NA |
| Saudi Arabia | National | 2001,2007,2010 | 9.4 (7.8-11.1) | 14.9 (12.1-17.8) | 5.5 | 3.1 | 0.30 |  | NA | NA | NA | NA | NA | NA |
| Sudan | National | 2001,2005,2009 | 31.3 (28.9-33.8) | 14.7 (12.8-16.5) | -16.6 | -10.4 | <0.001 |  | NA | NA | NA | NA | NA | NA |
| Syrian Arab Republic | National | 2002,2007,2010 | 26.7 (23.7-29.8) | 14.9 (12.2-17.5) | -11.8 | -7.4 | <0.001 |  | NA | NA | NA | NA | NA | NA |
| Tunisia | National | 2001,2007,2010,2017 | 21.2 (19.5-22.8) | 20.6 (17.9-23.3) | -0.6 | -0.2 | 0.48 |  | NA | NA | NA | NA | NA | NA |
| United Arab Emirates | National | 2002,2005,2013 | 17.0 (15.2-18.8) | 27.0 (23.7-30.2) | 10.0 | 4.5 | <0.001 |  | 2002,2005,2013 | 33.1 (30.3-36.0) | 25.3 (23.5-27.2) | -7.8 | -3.5 | <0.001 |
| West BANK | regional | 2005,2009,2016 | 18.6 (16.5-20.6) | 20.8 (15.9-25.7) | 2.2 | 1.0 | 0.72 |  | NA | NA | NA | NA | NA | NA |
| Yemen | National | 2003,2008,2014 | 21.6 (19.9-23.3) | 18.1 (13.9-22.3) | -3.5 | -1.6 | 0.81 |  | NA | NA | NA | NA | NA | NA |
| **Europe** |  |  |  |  |  |  |  |  |  |  |  |  |  |  |
| Albania | National | 2004,2009,2015 | 19.8 (17.4-22.1) | 24.6 (22.3-26.8) | 4.8 | 2.2 | 0.07 |  | 2004,2009,2015 | 15.7 (13.6-17.9) | 22.1 (19.9-24.2) | 6.4 | 2.9 | <0.001 |
| Bosnia and Herzegovina | National | 2003,2008,2013,2019 | 30.8 (28.3-33.3) | 45.6 (43.8-47.4) | 14.8 | 4.6 | <0.001 |  | 2003,2008,2013 | 16.2 (13.8-18.6) | 16.6 (15.2-18.0) | 0.4 | 0.2 | 0.59 |
| Bulgaria | National | 2002,2008,2015 | 25.1 (22.7-27.4) | 29.7 (27.6-31.7) | 4.6 | 1.8 | 0.001 |  | NA | NA | NA | NA | NA | NA |
| Croatia (Hrvatska) | National | 2003,2007,2011,2016 | 31.3 (28.5-34.0) | 43.6 (40.2-47.0) | 12.3 | 4.7 | <0.001 |  | 2003,2007,2011,2016 | 23.9 (21.3-26.5) | 26.7 (23.0-30.4) | 2.8 | 1.1 | 0.01 |
| Czech Republic | National | 2002,2007,2011,2016 | 46.2 (44.3-48.1) | 39.8 (36.9-42.7) | -6.4 | -2.3 | 0.005 |  | 2002,2007,2011 | 26.1 (24.0-28.1) | 33.8 (30.1-37.5) | 7.7 | 4.3 | <0.001 |
| Georgia | National | 2003,2008,2014,2017 | 17.3 (15.7-18.9) | 32.3 (26.8-37.9) | 15.0 | 5.4 | <0.001 |  | 2003,2008,2014,2017 | 23.9 (20.7-27.1) | 16.3 (12.0-20.5) | -7.6 | -2.7 | 0.03 |
| Italy | National | 2010,2014,2018 | 38.1 (35.1-41.1) | 40.8 (37.5-44.2) | 2.7 | 1.7 | 0.16 |  | 2010,2014,2018 | 19.3 (16.9-21.7) | 21.0 (18.2-23.7) | 1.7 | 1.1 | 0.03 |
| Kazakhstan | National | 2004,2009,2014 | 16.9 (15.3-18.6) | 12.4 (9.6-15.2) | -4.5 | -2.2 | 0.03 |  | 2004,2009,2014 | 21.9 (19.9-23.9) | 10.9 (7.7-14.1) | -11.0 | -5.5 | <0.001 |
| Kyrgyzstan | National | 2004,2008,2014,2019 | 22.0 (17.6-26.5) | 28.0 (25.4-30.6) | 6.0 | 2.0 | 0.004 |  | NA | NA | NA | NA | NA | NA |
| Latvia | National | 2002,2007,2011,2014,2019 | 27.8 (25.5-30.1) | 26.8 (24.8-28.7) | -1.0 | -0.3 | 0.14 |  | 2002,2007,2011,2014,2019 | 27.6 (25.0-30.1) | 23.9 (21.7-26.2) | -3.7 | -1.1 | 0.001 |
| Lithuania | National | 2001,2005,2009,2014,2018 | 28.2 (26.2-30.2) | 31.0 (28.3-33.6) | 2.8 | 0.8 | 0.19 |  | 2001,2005,2009,2014,2018 | 38.6 (35.1-42.1) | 32.4 (30.5-34.4) | -6.2 | -1.8 | <0.001 |
| Montenegro | National | 2004,2008,2014,2018 | 14.6 (12.0-17.1) | 37.6 (35.5-39.7) | 23.0 | 8.2 | <0.001 |  | 2004,2008,2014,2018 | 15.8 (12.7-18.9) | 16.6 (14.6-18.6) | 0.8 | 0.3 | 0.33 |
| Poland | National | 1999,2003,2009,2016 | 33.8 (31.8-35.9) | 41.5 (39.8-43.2) | 7.7 | 2.3 | 0.33 |  | 1999,2003,2009,2016 | 25.5 (23.6-27.4) | 22.7 (20.3-25.2) | -2.8 | -0.8 | 0.09 |
| Republic of Moldova | National | 2004,2008,2013,2019 | 18.8 (16.0-21.6) | 29.6 (27.4-31.9) | 10.8 | 3.6 | <0.001 |  | NA | NA | NA | NA | NA | NA |
| Romania | National | 2004,2009,2013,2017 | 49.2 (44.4-54.0) | 25.5 (23.6-27.4) | -23.7 | -9.1 | <0.001 |  | 2004,2009,2013,2017 | 24.3 (21.2-27.4) | 17.1 (15.6-18.7) | -7.2 | -2.8 | <0.001 |
| Russian Federation | Subnational | 1999,2002,2004,2015 | 28.7 (26.7-30.7) | 24.8 (20.6-28.9) | -3.9 | -1.2 | 0.07 |  | 1999,2002,2004 | 26.5 (25.1-28.0) | 30.4 (28.7-32.2) | 3.9 | 3.9 | 0.003 |
| San Marino | National | 2010,2014,2018 | 41.4 (36.8-46.0) | 44.7 (40.7-48.6) | 3.3 | 2.1 | 0.35 |  | 2010,2014,2018 | 17.3 (14.2-20.3) | 18.1 (14.3-22.0) | 0.8 | 0.5 | 0.81 |
| Serbia | National | 2008,2013,2017 | 37.9 (35.9-39.9) | 53.6 (52.0-55.2) | 15.7 | 8.7 | <0.001 |  | NA | NA | NA | NA | NA | NA |
| Slovakia | National | 2003,2007,2011,2016 | 48.7 (46.5-50.9) | 47.8 (45.0-50.7) | -0.9 | -0.3 | 0.56 |  | 2003,2007,2011,2016 | 25.9 (24.2-27.7) | 19.3 (17.4-21.2) | -6.6 | -2.5 | 0.001 |
| Slovenia | National | 2003,2007,2011,2017 | 45.7 (43.5-47.9) | 53.3 (50.5-56.1) | 7.6 | 2.7 | <0.001 |  | 2003,2007,2011 | 23.7 (20.9-26.5) | 27.7 (24.8-30.6) | 4.0 | 2.5 | 0.06 |
| Tajikistan | National | 2004,2014,2019 | 18.1 (14.0-22.2) | 24.3 (21.7-26.8) | 6.2 | 2.1 | 1.00 |  | NA | NA | NA | NA | NA | NA |
| Turkey | National | 2003,2009,2012,2017 | 15.0 (14.3-15.8) | 27.7 (26.8-28.5) | 12.7 | 4.5 | <0.001 |  | 2003,2009,2012,2017 | 28.4 (27.2-29.6) | 32.7 (31.8-33.5) | 4.3 | 1.5 | <0.001 |
| Ukraine | National | 1999,2005,2011,2017 | 26.1 (24.3-27.9) | 25.7 (20.4-30.9) | -0.4 | -0.1 | 0.67 |  | 1999,2005,2011 | 26.5 (24.1-28.9) | 32.8 (29.4-36.2) | 6.3 | 2.6 | 0.24 |
| **South-East Asia** |  |  |  |  |  |  |  |  |  |  |  |  |  |  |
| Bangladesh | National | 2004,2007,2013 | 34.6 (31.9-37.3) | 33.1 (23.1-43.0) | -1.5 | -0.8 | 0.82 |  | NA | NA | NA | NA | NA | NA |
| Bhutan | National | 2004,2006,2009,2013,2019 | 17.9 (15.9-19.8) | 16.4 (14.6-18.2) | -1.5 | -0.5 | 0.003 |  | 2004,2006,2009 | 40.4 (35.4-45.4) | 61.9 (57.3-66.6) | 21.5 | 21.5 | <0.001 |
| India | National | 2000,2002,2003,2004,2006,2009 | 35.0 (33.3-36.8) | 22.9 (20.9-25.0) | -12.1 | -6.7 | <0.001 |  | 2000,2002,2003,2004,2006,2009 | 36.4 (34.4-38.4) | 29.7 (27.3-32.1) | -6.7 | -3.7 | <0.001 |
| Indonesia | National | 2000,2004,2005,2006,2009,2014,2019 | 24.4 (21.0-27.8) | 12.8 (11.6-13.9) | -11.6 | -3.1 | <0.001 |  | 2000,2004,2005,2006,2009,2014 | 13.1 (11.1-15.1) | 19.5 (15.8-23.2) | 6.4 | 2.3 | 0.90 |
| Maldives | National | 2004,2007,2011,2019 | 25.6 (22.3-28.8) | 18.5 (16.6-20.3) | -7.1 | -2.4 | <0.001 |  | 2004,2007,2011 | 42.8 (38.8-46.8) | 36.8 (32.8-40.8) | -6.0 | -4.3 | 0.37 |
| Myanmar | National | 2001,2004,2007,2011,2016 | 9.0 (7.7-10.3) | 25.8 (20.5-31.1) | 16.8 | 5.6 | <0.001 |  | 2001,2004,2007,2011 | 58.7 (56.0-61.4) | 40.5 (36.2-44.7) | -18.2 | -9.1 | <0.001 |
| Nepal | National | 2001,2004,2007,2011 | 26.5 (22.6-30.3) | 53.7 (49.3-58.0) | 27.2 | 13.6 | <0.001 |  | 2001,2004,2007,2011 | 38.9 (34.8-42.9) | 55.8 (50.6-61.1) | 16.9 | 8.4 | <0.001 |
| Sri Lanka | National | 1999,2003,2007,2011,2015 | 42.2 (39.7-44.8) | 24.3 (18.7-29.8) | -17.9 | -5.6 | <0.001 |  | 1999,2003,2007,2011 | 52.3 (49.1-55.5) | 53.8 (51.5-56.0) | 1.5 | 0.6 | 0.01 |
| Thailand | National | 2005,2009,2015 | 28.0 (25.0-31.0) | 28.1 (26.0-30.2) | 0.1 | 0.1 | 0.19 |  | 2005,2009,2015 | 39.8 (36.8-42.7) | 23.9 (20.2-27.6) | -15.9 | -7.9 | <0.001 |
| Timor-Leste | National | 2006,2009,2013,2019 | 37.0 (32.0-42.0) | 39.1 (36.3-41.8) | 2.1 | 0.8 | 0.78 |  | NA | NA | NA | NA | NA | NA |
| Western Pacific |  |  |  |  |  |  |  |  |  |  |  |  |  |  |
| Cambodia | National | 2003,2010,2016 | 18.9 (16.2-21.5) | 10.8 (8.6-12.9) | -8.1 | -3.1 | 0.01 |  | 2003,2010,2016 | 18.9 (15.5-22.2) | 9.4 (7.1-11.7) | -9.5 | -3.7 | <0.001 |
| Cook Islands | National | 2003,2008,2016 | 36.0 (31.7-40.4) | 29.7 (25.5-33.9) | -6.3 | -2.4 | 0.10 |  | 2003,2008,2016 | 51.8 (47.4-56.2) | 27.4(23.4-31.4) | -24.4 | -9.4 | <0.001 |
| Fiji | National | 1999,2005,2009,2016 | 39.5 (34.4-44.6) | 27.2 (24.1-30.3) | -12.3 | -3.6 | <0.001 |  | 1999,2005,2009,2016 | 54.7 (46.7-62.7) | 40.3 (35.3-45.4) | -14.4 | -4.2 | <0.001 |
| Guam | National | 2011,2014,2017 | 30.5 (26.6-34.4) | 26.5 (24.3-28.7) | -4.0 | -3.3 | 0.35 |  | NA | NA | NA | NA | NA | NA |
| Laos | National | 2003,2007,2011,2016 | 20.7 (18.9-22.5) | 41.8 (39.4-44.2) | 21.1 | 8.1 | <0.001 |  | 2003,2007,2011,2016 | 21.2 (18.7-23.6) | 35.2 (31.9-38.6) | 14.0 | 5.4 | <0.001 |
| Macau (China) | National | 2001,2005,2010,2015 | 6.7 (5.4-8.0) | 7.9 (3.6-12.3) | 1.2 | 0.4 | 0.40 |  | 2001,2005,2010,2015 | 16.4 (13.6-19.3) | 6.3 (5.0-7.6) | -10.1 | -3.6 | <0.001 |
| Mongolia | National | 2003,2007,2014,2019 | 20.4 (18.6-22.2) | 6.8 (5.4-8.2) | -13.6 | -4.2 | <0.001 |  | 2003,2007,2019 | 30.3 (27.5-33.1) | 18.7 (16.5-20.9) | -11.6 | -3.6 | <0.001 |
| Micronesia | National | 2007,2013,2019 | 35.0 (32.1-37.8) | 32.2 (30.7-33.8) | -2.8 | -1.2 | 0.10 |  | 2007,2013,2019 | 50.2 (45.7-54.7) | 50.7 (48.8-52.5) | 0.5 | 0.2 | 0.93 |
| Philippines | National | 2000,2004,2007,2011,2015,2019 | 23.1 (21.6-24.5) | 16.9 (15.4-18.3) | -6.2 | -1.6 | 0.04 |  | 2000,2004,2007,2011,2015,2019 | 27.4 (25.2-29.5) | 33.0 (31.0-34.9) | 5.6 | 1.5 | 0.03 |
| South Korea | National | 2005,2008,2013 | 5.9 (5.2-6.5) | 9.5 (8.4-10.5) | 3.6 | 2.2 | <0.001 |  | NA | NA | NA | NA | NA | NA |

Abbreviation: NA, not available.

**Table S14 Trends in the proportions of adolescents aged 12–16 years who believe that tobacco smoking makes people more attractive, from 1999 to 2019, by country/territory.**

| **Country/territory** | **Tobacco smoking makes more friends** | | | | | | |
| --- | --- | --- | --- | --- | --- | --- | --- |
|  | **Representativeness** | **Survey year** | **First year, %**  **(95%CI)** | **Last year, %**  **(95%CI)** | **Total absolute change, %** | **Absolute change/5-years, %** | ***P* for trend** |
|  |  |  |  |  |  |  |  |
| **Africa** |  |  |  |  |  |  |  |
| Burkina Faso | Subnational | 2001,2006,2009 | 21.5  (19.1-24.0) | 20.3  (18.1-22.6) | -1.2 | -0.7 | 0.33 |
| Congo | National | 2006,2009,2019 | 39.2  (34.1-44.3) | 25.4  (22.3-28.5) | -13.8 | -5.3 | <0.001 |
| Ghana | National | 2000,2006,2009 | 21.7  (16.9-26.5) | 18.2  (14.8-21.6) | -3.5 | -1.9 | 0.18 |
| Malawi | National | 2000,2005,2009 | 4.1  (2.7-5.4) | 2.6  (1.5-3.8) | -1.5 | -0.8 | 0.02 |
| Mauritania | National | 2001,2006,2009,2018 | 36.6  (33.6-39.6) | 28.8  (26.4-31.2) | -7.8 | -2.3 | <0.001 |
| Mauritius | National | 2003,2008,2016 | 15.7  (12.8-18.5) | 10.8  (8.0-13.5) | -4.9 | -1.9 | 0.01 |
| Niger | National | 2001,2006,2009 | 34.9  (30.1-39.6) | 40.1  (31.4-48.8) | 5.2 | 3.3 | 0.20 |
| Seychelles | National | 2002,2007,2015 | 17.7  (13.9-21.4) | 13.1  (11.3-14.9) | -4.6 | -1.8 | 0.007 |
| South Africa | National | 1999,2002,2008,2011 | 24.3  (19.6-29.1) | 26.0  (23.8-28.3) | 1.7 | 0.7 | 0.07 |
| Swaziland | National | 2001,2005,2009 | 16.5  (14.7-18.3) | 20.5  (18.3-22.7) | 4.0 | 2.5 | 0.002 |
| Togo | National | 2002,2007,2013,2019 | 12.5  (9.3-15.7) | 11.1  (8.1-14.1) | -1.4 | -0.4 | 0.05 |
| Uganda | National | 2002,2007,2011 | 11.4  (9.3-13.5) | 16.6  (12.6-20.6) | 5.2 | 2.9 | 0.03 |
| Zambia | National | 2002,2007,2011 | 30.7  (26.9-34.4) | 26.9  (25.2-28.5) | -3.8 | -2.1 | 0.10 |
| Zimbabwe | National | 1999,2003,2008,2014 | 21.2  (17.9-24.5) | 17.1  (13.1-21.0) | -4.1 | -1.4 | 0.06 |
| **Americas** |  |  |  |  |  |  |  |
| Antigua and Barbuda | National | 2000,2004,2009,2017 | 8.1  (6.2-9.9) | 8.1  (6.5-9.6) | 0.0 | 0.0 | 0.97 |
| Argentina | National | 2000,2007,2018 | 15.1  (13.1-17.2) | 8.7  (3.9-13.4) | -6.4 | -1.8 | <0.001 |
| Bahamas | National | 2000,2004,2009,2013 | 9.7  (7.8-11.6) | 16.0  (2.7-29.4) | 6.3 | 2.4 | 0.38 |
| Barbados | National | 1999,2002,2007 | 7.2  (5.2-9.1) | 14.0  (12.0-16.0) | 6.8 | 4.2 | 0.007 |
| Bolivia | Subnational | 2000,2003,2012,2018 | 18.6  (17.5-19.7) | 14.3  (12.1-16.5) | -4.3 | -1.2 | <0.001 |
| Brazil | Subnational | 2002,2005,2006,2007 | 7.3  (6.5-8.1) | 9.7  (6.1-13.3) | 2.4 | 2.4 | 0.75 |
| Chile | Subnational | 2000,2003,2008 | 15.0  (13.5-16.5) | 13.0  (12.1-14.0) | -2.0 | -1.2 | 0.002 |
| Costa Rica | National | 1999,2002,2008,2013 | 6.5  (5.7-7.2) | 6.8  (5.5-8.1) | 0.3 | 0.1 | 0.74 |
| Cuba | National | 2000,2004,2010,2018 | 11.9  (10.0-13.7) | 7.6  (6.2-9.0) | -4.3 | -1.2 | 0.009 |
| Dominica | National | 2000,2004,2009 | 13.1  (11.2-15.1) | 15.4  (12.4-18.4) | 2.3 | 1.3 | 0.37 |
| Grenada | National | 2000,2004,2009 | 11.4  (10.1-12.7) | 11.9  (10.0-13.9) | 0.5 | 0.3 | 1.00 |
| Guatemala | National | 2002,2008,2015 | 11.6  (9.3-13.9) | 15.1  (12.8-17.3) | 3.5 | 1.3 | 0.65 |
| Guyana | National | 2000,2004,2010 | 9.3  (6.5-12.1) | 14.4  (11.0-17.9) | 5.1 | 2.5 | 0.04 |
| Jamaica | National | 2000,2006,2010 | 12.5  (9.7-15.3) | 16.2  (11.4-21.1) | 3.7 | 1.8 | 0.06 |
| Mexico | Subnational | 2003,2005,2006,2008,2011 | 31.9  (30.6-33.2) | 20.3  (17.8-22.8) | -11.6 | -7.2 | <0.001 |
| Nicaragua | National | 2003,2014,2019 | 15.7  (13.8-17.7) | 10.6  (9.4-11.8) | -5.1 | -1.6 | <0.001 |
| Panama | National | 2002,2008,2012 | 11.3  (9.4-13.1) | 11.8  (10.0-13.6) | 0.5 | 0.2 | 0.36 |
| Peru | National | 2000,2002,2003,2007 | 12.8  (11.1-14.5) | 14.3  (13.2-15.4) | 1.5 | 1.1 | 0.90 |
| Saint Lucia | National | 2000,2007,2011,2017 | 11.8  (8.7-14.9) | 11.0  (8.7-13.2) | -0.8 | -0.2 | 0.99 |
| Saint Vincent and the Grenadines | National | 2000,2007,2011,2018 | 10.3  (8.1-12.5) | 7.9  (6.5-9.3) | -2.4 | -0.7 | 0.61 |
| Suriname | National | 2000,2004,2009 | 27.5  (22.3-32.6) | 42.9  (39.6-46.2) | 15.4 | 8.6 | <0.001 |
| Trinidad and Tobago | National | 2000,2007,2011 | 15.6  (13.3-17.8) | 9.8  (8.2-11.4) | -5.8 | -2.6 | <0.001 |
| Venezuela | National | 1999,2001,2003,2008,2010 | 6.9  (6.0-7.8) | 3.3  (2.4-4.1) | -3.6 | -1.6 | <0.001 |
| **Eastern Mediterranean** |  |  |  |  |  |  |  |
| Egypt | National | 2001,2005,2009,2014 | 28.7  (24.2-33.2) | 13.7  (8.5-18.9) | -15.0 | -5.8 | <0.001 |
| Gaza Strip | Subnational | 2000,2005,2013,2019 | 38.6  (35.2-42.0) | 16.0  (9.9-22.2) | -22.6 | -5.9 | <0.001 |
| Iraq | National | 2006,2008,2014 | 37.6  (32.2-42.9) | 13.4  (9.7-17.1) | -24.2 | -15.1 | <0.001 |
| Jordan | National | 1999,2003,2007,2009,2014 | 27.7  (24.8-30.6) | 19.4  (15.7-23.2) | -8.3 | -2.8 | <0.001 |
| Kuwait | National | 2001,2005,2016 | 28.0  (26.1-29.8) | 20.5  (18.2-22.7) | -7.5 | -2.5 | <0.001 |
| Lebanon | National | 2001,2005,2011 | 14.6  (12.5-16.6) | 17.2  (15.2-19.3) | 2.6 | 1.3 | 0.47 |
| Libyan Arab Jamahiriya | National | 2003,2007,2010 | 23.3  (20.7-25.8) | 19.7  (16.9-22.5) | -3.6 | -2.6 | 0.07 |
| Oman | National | 2002,2010,2016 | 29.3  (24.9-33.6) | 16.1  (12.7-19.5) | -13.2 | -4.7 | <0.001 |
| Pakistan | National | 2004,2008,2013 | 16.6  (12.9-20.3) | 39.2  (32.8-45.6) | 22.6 | 12.6 | <0.001 |
| Saudi Arabia | National | 2001,2007,2010 | 23.8  (21.5-26.1) | 25.2  (21.2-29.2) | 1.4 | 0.8 | 0.25 |
| Sudan | National | 2001,2005,2009 | 31.1  (27.9-34.3) | 19.5  (15.7-23.2) | -11.6 | -7.3 | <0.001 |
| Syrian Arab Republic | National | 2002,2007,2010 | 14.5  (11.9-17.2) | 18.5  (14.8-22.2) | 4.0 | 2.5 | 0.07 |
| Tunisia | National | 2001,2007,2010 | 24.2  (22.8-25.5) | 21.3  (18.1-24.5) | -2.9 | -1.6 | 0.79 |
| West BANK | regional | 2000,2005,2009,2016 | 40.0  (38.1-41.8) | 19.4  (14.6-24.2) | -20.6 | -6.4 | <0.001 |
| Yemen | National | 2003,2008,2014 | 29.7  (28.2-31.2) | 13.7  (10.4-17.0) | -16.0 | -7.3 | <0.001 |
| **Europe** |  |  |  |  |  |  |  |
| Bosnia and Herzegovina | National | 2003,2008,2013 | 16.1  (13.8-18.3) | 15.0  (13.7-16.3) | -1.1 | -0.6 | 0.45 |
| Croatia (Hrvatska) | National | 2003,2007,2011,2016 | 16.1  (13.6-18.6) | 21.1  (18.1-24.2) | 5.0 | 1.9 | 0.01 |
| Czech Republic | National | 2002,2007,2011 | 14.5  (12.7-16.4) | 13.0  (10.0-15.9) | -1.5 | -0.8 | 0.35 |
| Georgia | National | 2003,2008,2014,2017 | 31.2  (28.0-34.4) | 11.4  (8.9-13.9) | -19.8 | -7.1 | <0.001 |
| Italy | National | 2010,2014,2018 | 16.5  (14.0-19.0) | 14.0  (11.6-16.4) | -2.5 | -1.6 | 0.12 |
| Kazakhstan | National | 2004,2009,2014 | 16.2  (14.3-18.2) | 12.3  (7.6-17.0) | -3.9 | -1.9 | 0.05 |
| Latvia | National | 2002,2007,2011,2014 | 9.9  (8.3-11.4) | 6.9  (4.9-9.0) | -3.0 | -1.2 | 0.03 |
| Lithuania | National | 2001,2005,2009,2014,2018 | 11.2  (9.1-13.4) | 8.8  (7.5-10.1) | -2.4 | -0.7 | 0.02 |
| Montenegro | National | 2004,2008,2014,2018 | 12.4  (9.5-15.2) | 13.6  (11.9-15.3) | 1.2 | 0.4 | 0.24 |
| Poland | National | 1999,2003,2009,2016 | 7.9  (6.9-9.0) | 6.8  (5.3-8.3) | -1.1 | -0.3 | 0.22 |
| Romania | National | 2004,2009,2013,2017 | 27.3  (21.6-33.1) | 9.9  (8.7-11.0) | -17.4 | -6.7 | <0.001 |
| Russian Federation | Subnational | 1999,2002,2004 | 12.6  (11.0-14.1) | 11.7  (10.6-12.8) | -0.9 | -0.9 | 0.14 |
| San Marino | National | 2010,2014,2018 | 10.6  (8.2-13.0) | 13.7  (11.0-16.4) | 3.1 | 1.9 | 0.09 |
| Slovakia | National | 2003,2007,2011,2016 | 17.6  (15.9-19.3) | 9.8  (8.2-11.5) | -7.8 | -3.0 | <0.001 |
| Slovenia | National | 2003,2007,2011 | 12.2  (10.5-13.8) | 10.2  (7.5-12.9) | -2.0 | -1.2 | 0.16 |
| Tajikistan | National | 2004,2014,2019 | 25.0  (20.0-30.0) | 51.8  (44.3-59.3) | 26.8 | 8.9 | <0.001 |
| Turkey | National | 2003,2009,2012 | 23.2  (22.0-24.4) | 16.4  (14.2-18.6) | -6.8 | -3.8 | <0.001 |
| Ukraine | National | 1999,2005,2011 | 12.1  (10.7-13.6) | 8.9  (7.0-10.9) | -3.2 | -1.3 | <0.001 |
| **South-East Asia** |  |  |  |  |  |  |  |
| Bhutan | National | 2004,2006,2009 | 18.1  (16.1-20.1) | 24.5  (21.0-27.9) | 6.4 | 6.4 | 0.003 |
| India | National | 2000,2002,2003,2004,2006,2009 | 28.2  (26.6-29.7) | 25.5  (23.3-27.6) | -2.7 | -1.5 | <0.001 |
| Indonesia | National | 2000,2004,2005,2006,2009,2014 | 10.8  (8.6-13.0) | 6.0  (4.6-7.3) | -4.8 | -1.7 | <0.001 |
| Maldives | National | 2004,2007,2011 | 21.7  (17.8-25.5) | 14.9  (12.8-16.9) | -6.8 | -4.9 | 0.50 |
| Myanmar | National | 2001,2004,2007,2011 | 38.0  (35.3-40.7) | 37.5  (32.3-42.7) | -0.5 | -0.2 | 0.26 |
| Nepal | National | 2001,2004,2007,2011 | 28.5  (23.7-33.3) | 53.0  (49.6-56.4) | 24.5 | 12.2 | <0.001 |
| Sri Lanka | National | 1999,2003,2007,2011 | 50.7  (47.7-53.7) | 13.2  (11.6-14.8) | -37.5 | -15.6 | <0.001 |
| Thailand | National | 2005,2009,2015 | 13.3  (12.0-14.7) | 11.9  (9.2-14.7) | -1.4 | -0.7 | 0.58 |
| **Western Pacific** |  |  |  |  |  |  |  |
| Cambodia | National | 2003,2010,2016 | 2.4  (1.3-3.6) | 11.8  (9.1-14.4) | 9.4 | 3.6 | <0.001 |
| Cook Islands | National | 2003,2008,2016 | 19.7  (15.5-23.9) | 10.1  (7.4-12.9) | -9.6 | -3.7 | 0.02 |
| Fiji | National | 1999,2005,2009,2016 | 16.7  (12.6-20.7) | 19.4  (15.6-23.1) | 2.7 | 0.8 | 0.14 |
| Laos | National | 2003,2007,2011,2016 | 13.1  (11.5-14.6) | 26.4  (23.6-29.3) | 13.3 | 5.1 | <0.001 |
| Macau (China) | National | 2001,2005,2010,2015 | 14.2  (12.1-16.2) | 6.5  (5.3-7.8) | -7.7 | -2.7 | <0.001 |
| Mongolia | National | 2003,2007,2014,2019 | 50.7  (48.8-52.5) | 3.4  (2.6-4.3) | -47.3 | -14.8 | <0.001 |
| Micronesia | National | 2007,2013,2019 | 29.1  (24.7-33.6) | 27.7  (26.0-29.4) | -1.4 | -0.6 | 0.78 |
| Philippines | National | 2000,2004,2007,2011,2015,2019 | 14.8  (12.9-16.6) | 14.7  (13.0-16.4) | -0.1 | -0.0 | <0.001 |
